# Supplementary figures and images for: Adaptive β-lactam resistance from an inducible efflux pump that is post-translationally regulated by the DjlA co-chaperone
Source: PLoS Biol. 2023 Dec 5;21(12):e3002040. doi: 10.1371/journal.pbio.3002040 (PMC10754441; doi:10.1371/journal.pbio.3002040)

A

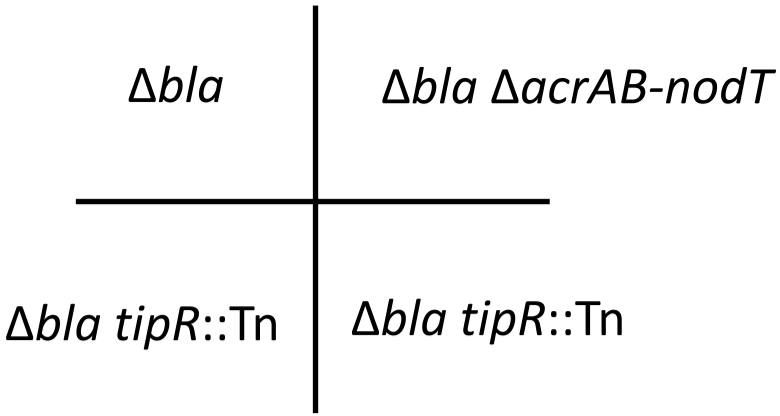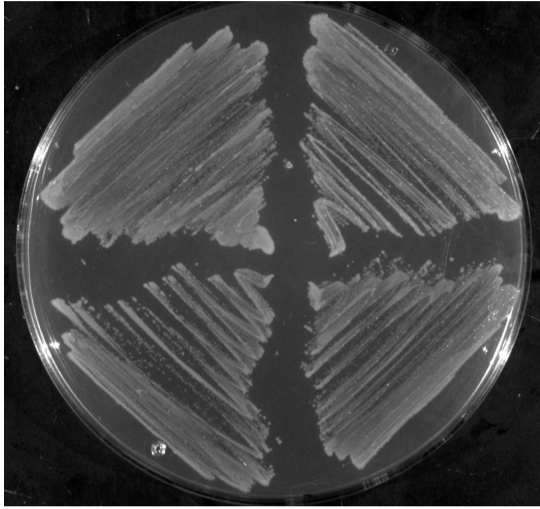

NAL<sup>10</sup>

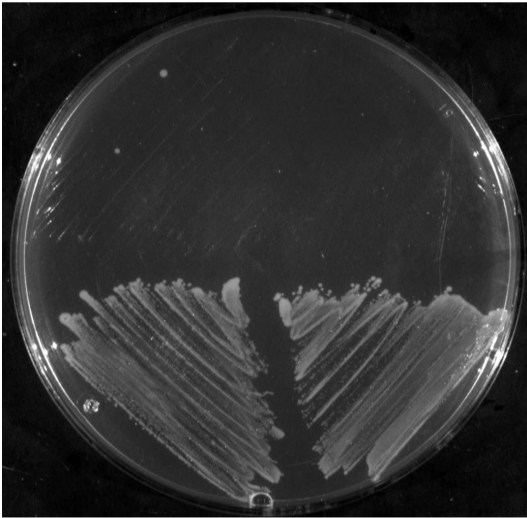

CEF<sup>10</sup>

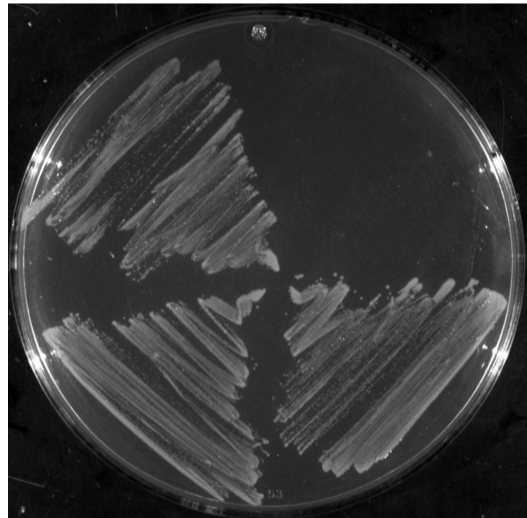

NAL<sup>10</sup>  
+  
CEF<sup>10</sup>

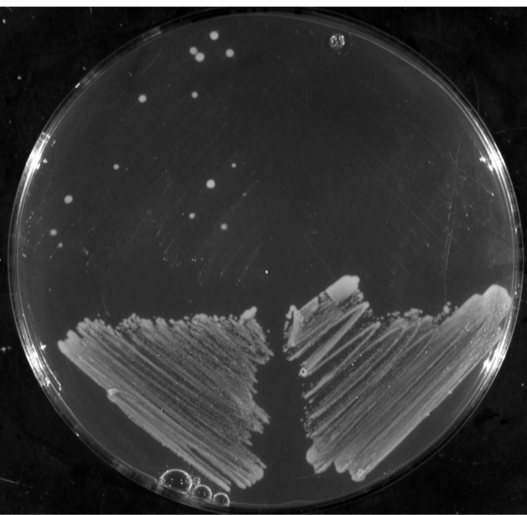

PIR<sup>40</sup>

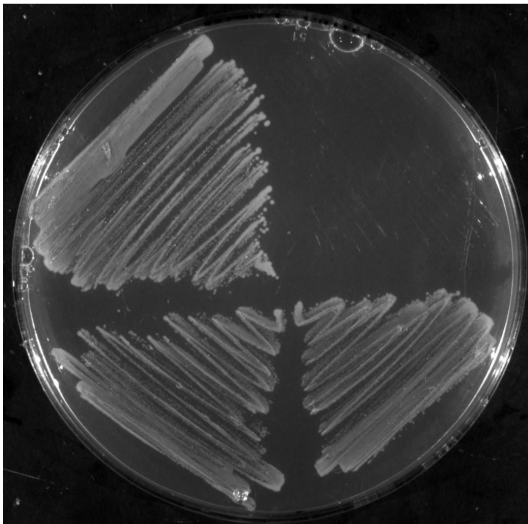

NAL<sup>10</sup>  
+  
PIR<sup>40</sup>

B

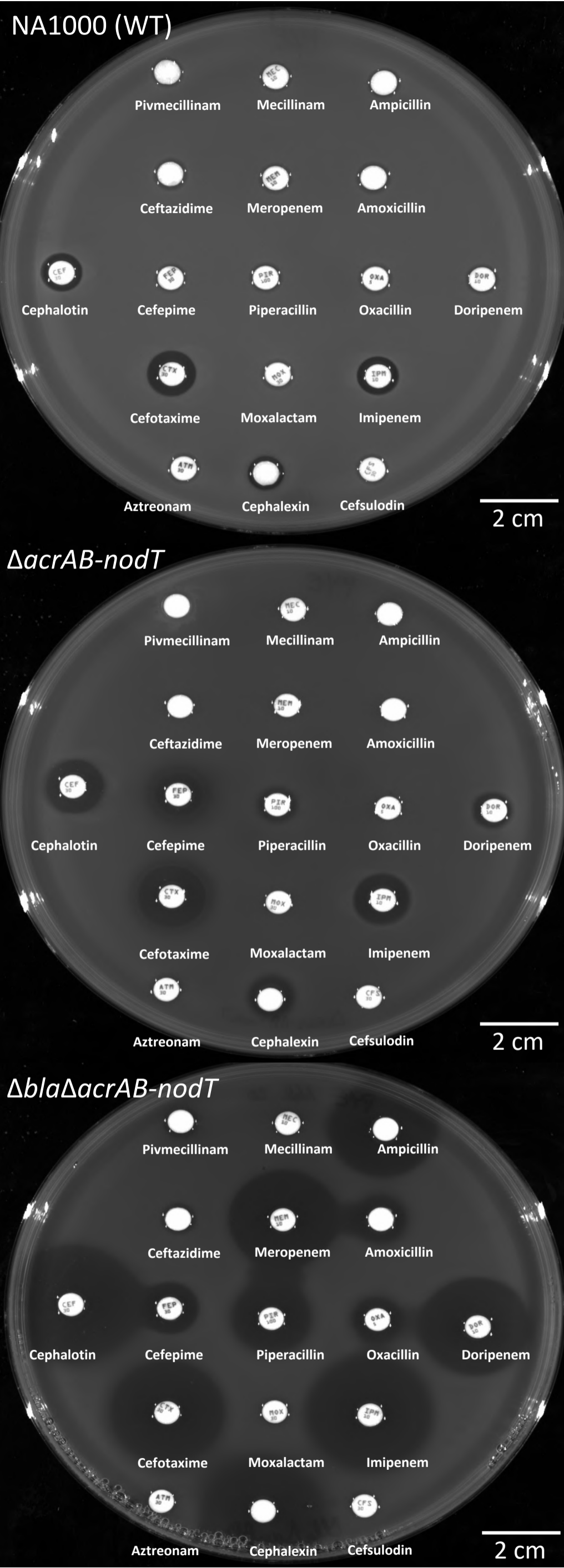

Supplement: S1 Fig — (A) Growth of C. crescentus mutant strains on plates containing NAL10 (nalidixic acid, 10 μg/mL), CEF10 (cephalothin, 10 μg/mL), and/or PIR40 (piperacillin, 40 μg/mL) for 3 days. (B) Antibiograms of C. crescentus strains on PYE. Antibiotic discs, from top left to bottom right: Pivmecillinam 20 μg, Mecillinam 10 μg, Ampicillin 100 μg, Ceftazidime 40 μg, Meropenem 10 μg, Amoxicillin 4 μg, Cephalothin 30 μg, Cefepime 30 μg, Piperacillin 100 μg, Oxacillin 5 μg, Doripenem 10 μg, Cefotaxime 30 μg, Moxalactam 30 μg, Imipenem 10 μg, Aztreonam 30 μg, Cephalexin 40 μg, Cefsulodin 30 μg. (PDF) [file pbio.3002040.s001.pdf]

**A**

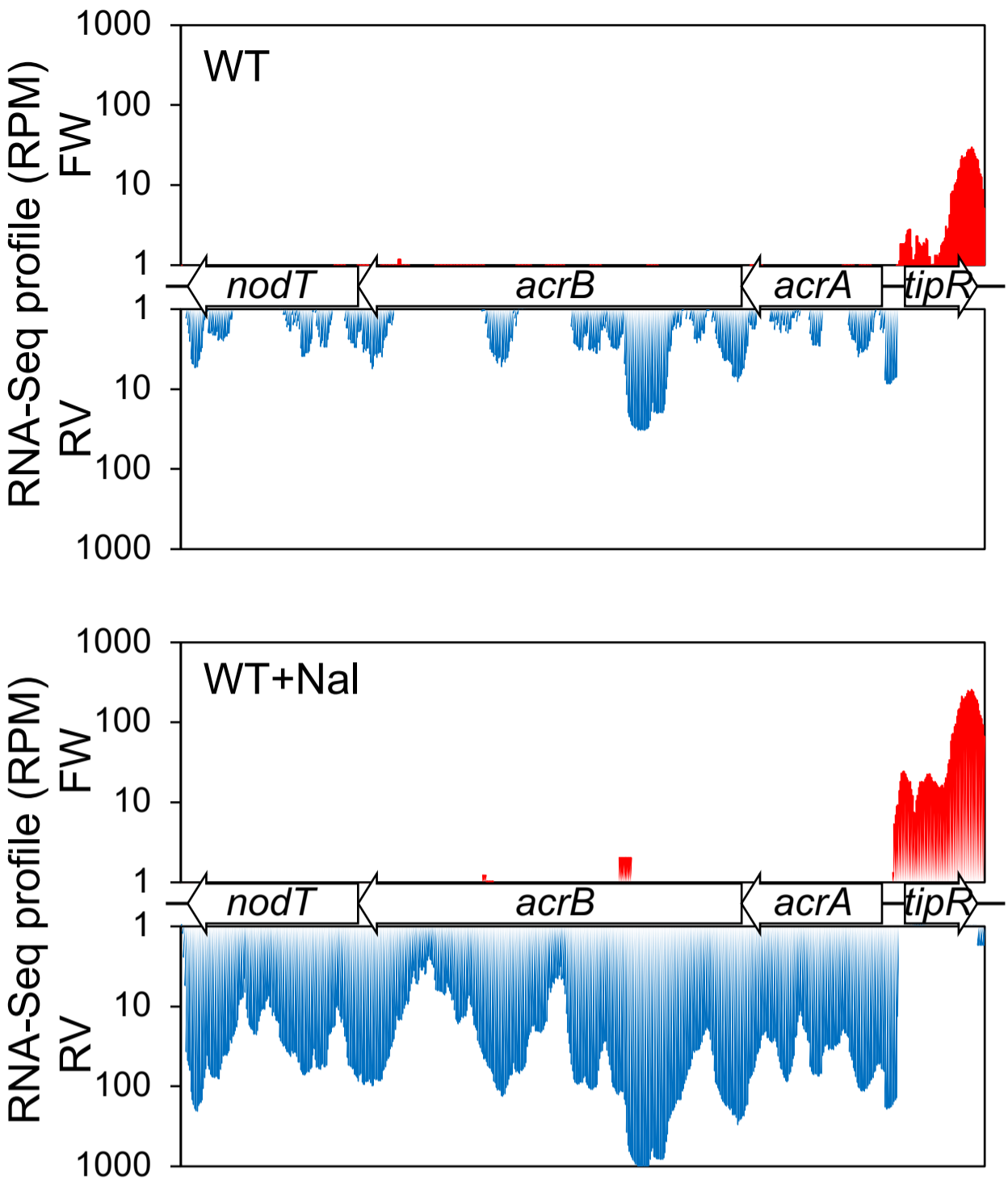

**B**

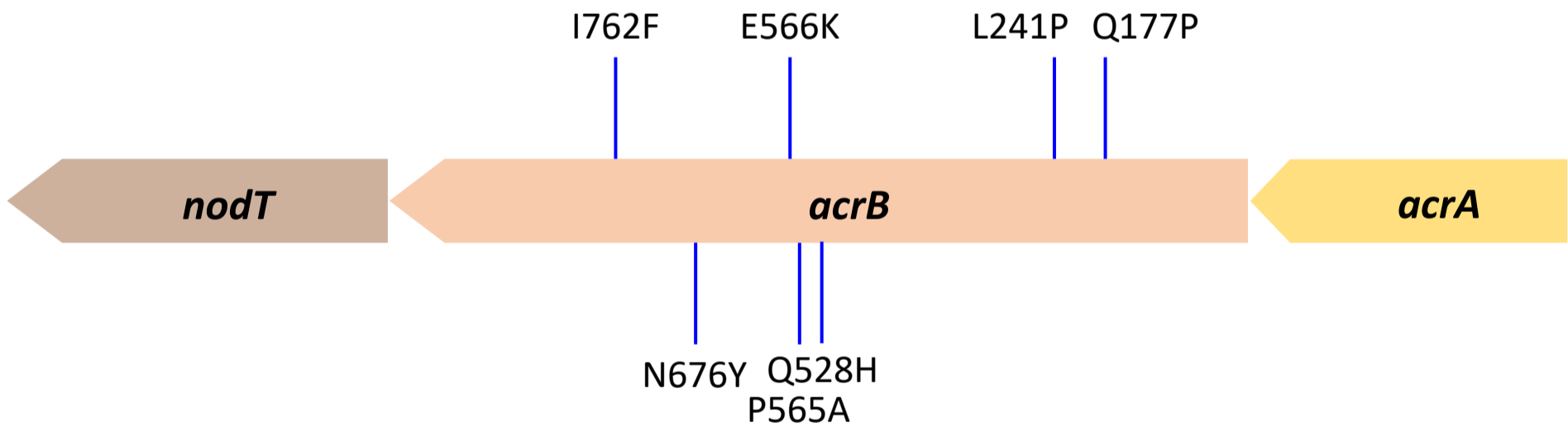

Supplement: S2 Fig — (A) Representation of the reads [represented as reads per million (RPM)] obtained from the RNA-Seq experiment covering the tipR and acrAB-nodT region in the NA1000 strain (WT). Induction was performed on exponentially grown cells in PYE for 30 minutes with nalidixic acid (Nal, 20μg/mL). Red curves represent the reads in forward orientation (FW); blue curves represent the reads in reverse orientation (RV). The data from the analysis are deposited in S1 Data. (B) Scheme showing the location of the AcrB mutations conferring high level of cephalothin resistance (CEF40) to Δbla;tipR::Tn cells. (PDF) [file pbio.3002040.s002.pdf]

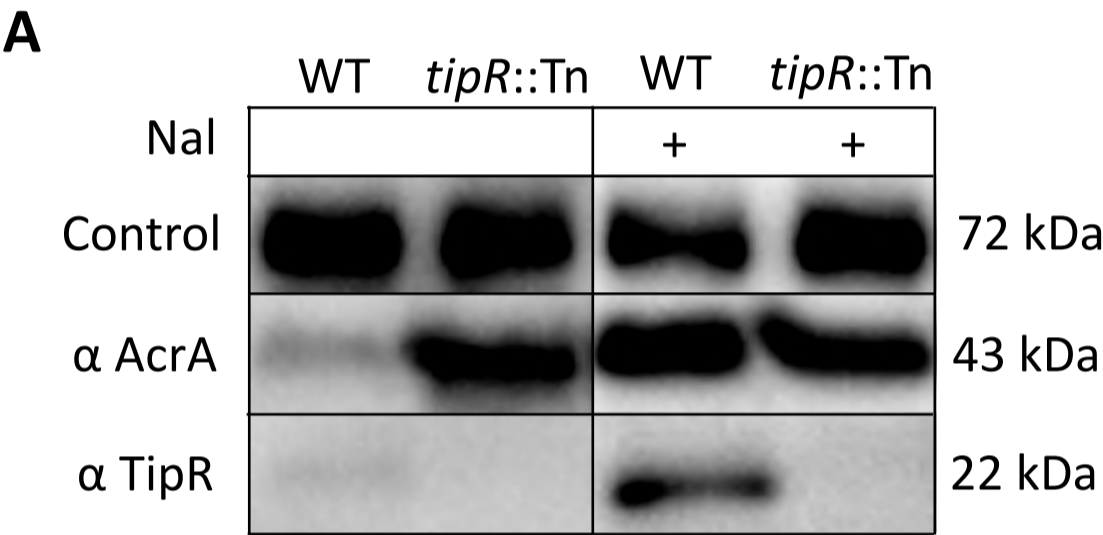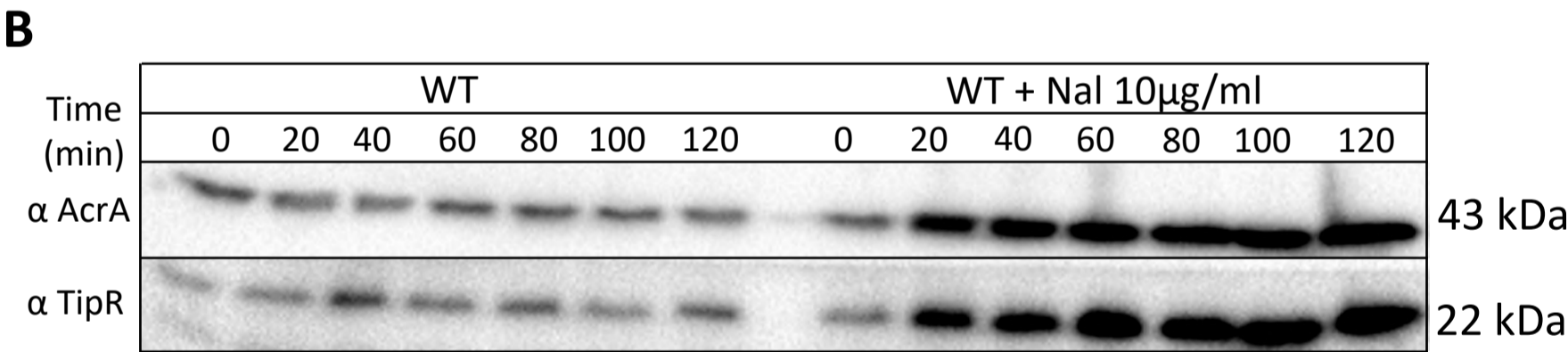

Supplement: S3 Fig — (A) Immunoblot with polyclonal antibodies to AcrA and to TipR to probe extracts from NA1000 (WT) and tipR mutant (tipR::Tn) cells exponentially grown in PYE before and after 2 hours of induction of Nal (20 μg/mL). Loading control represents CCNA_00163 revealed with antibodies to CCNA_00163. (B) Immunoblot polyclonal antibodies to AcrA and to TipR to probe extracts of cells harvested during a time course experiment on NA1000 (WT) for 2 hours in the presence and absence of nalidixic acid (Nal, 10 μg/mL) in PYE. (PDF) [file pbio.3002040.s003.pdf]

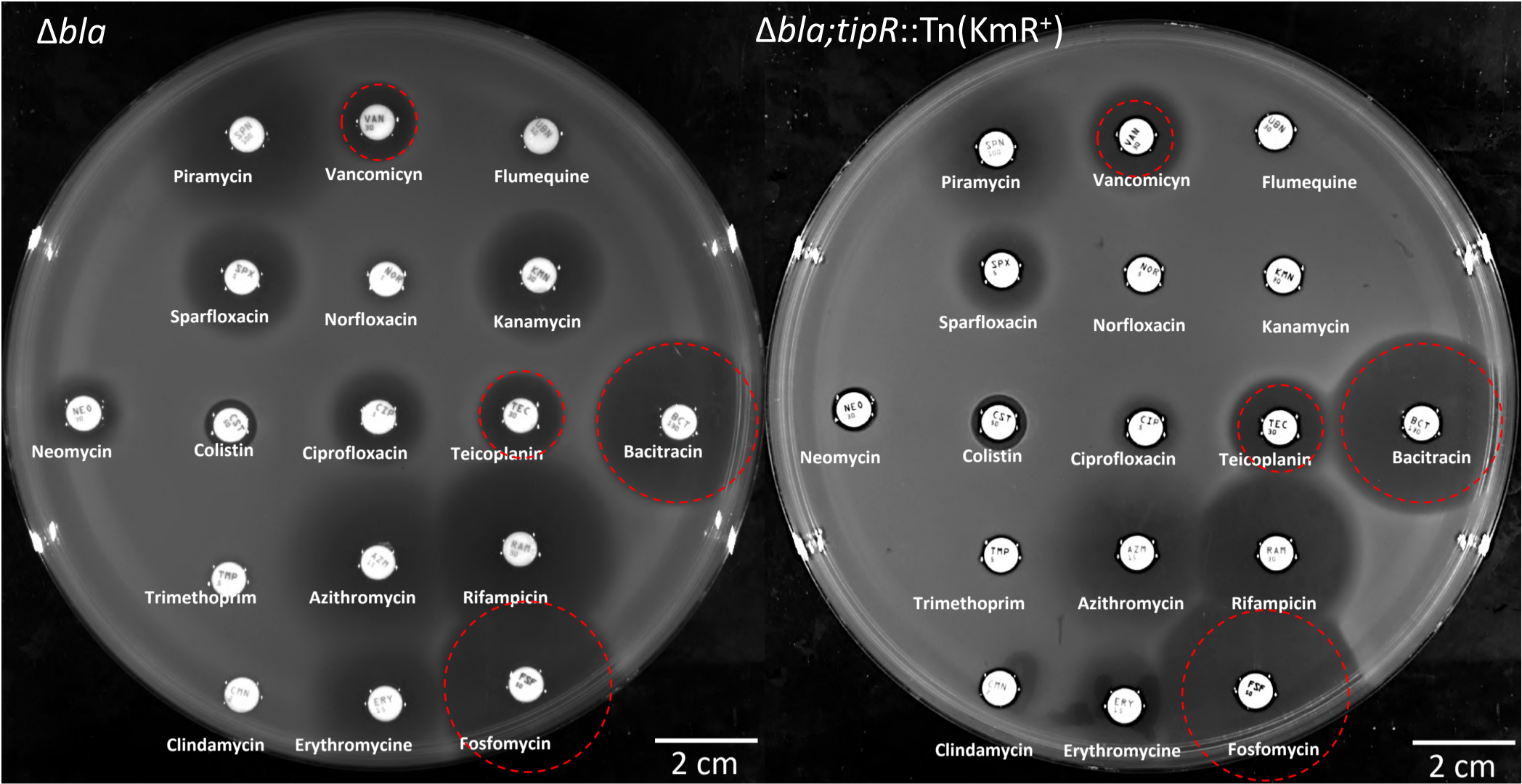

Supplement: S4 Fig — Kirby–Bauer-based disc diffusion assays to establish antibiograms of C. crescentus strains on PYE. Antibiotic discs, from top left to bottom right: Piramycin 100 μg, Vancomycin 30 μg, Flumequine 30 μg, Sparfloxacin 5 μg, Norfloxacin 5 μg, Kanamycin 20 μg, Neomycin 30 μg, Colistin 50 μg, Ciprofloxacin 5 μg, Teicoplanin 30 μg, Bacitracin 130 μg, Trimethoprim 5 μg, Azithromycin 15 μg, Rifampicin 30 μg, Clindamycin 2 μg, Erythromycin 15 μg, Fosfomycin 50 μg. Note: Kanamycin and neomycin resistance is conferred by the nptII gene located the transposon inserted in the tipR gene. Red circles demarcate the growth boundary for the reference strain. (PDF) [file pbio.3002040.s004.pdf]

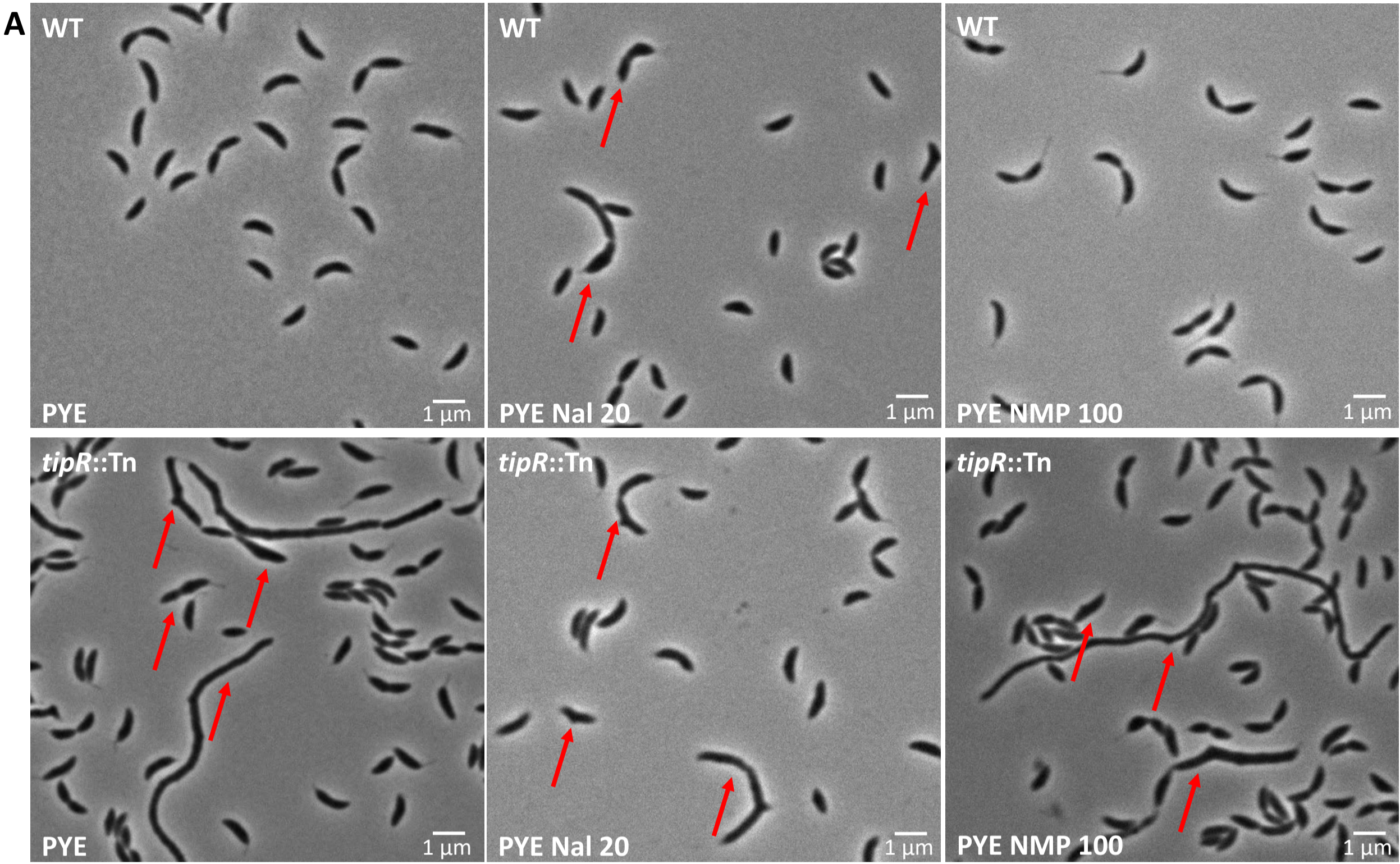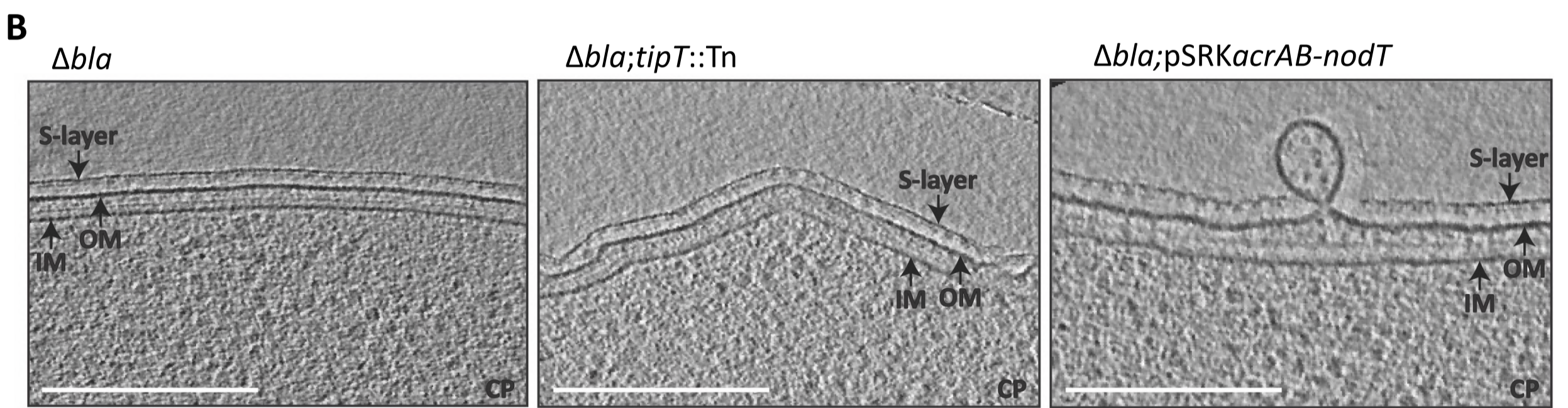

Supplement: S5 Fig — (A) Light microscopy (phase contrast) images of NA1000 (WT) and tipR::Tn cells during exponential growth in PYE in the presence or absence of Nalidixic acid (Nal, 10 μg/mL), 1-(1-Naphtylmethyl)-piperazine (NMP quantities are indicated. (B) Cryo-ET images C. crescentus Δbla cells (left), Δbla; tipR::Tn (center), and Δbla; pSRK-acrAB-nodT (right). Scale bar, 250 nm. CP, cytoplasm; IM, inner membrane; OM, outer membrane. (PDF) [file pbio.3002040.s005.pdf]

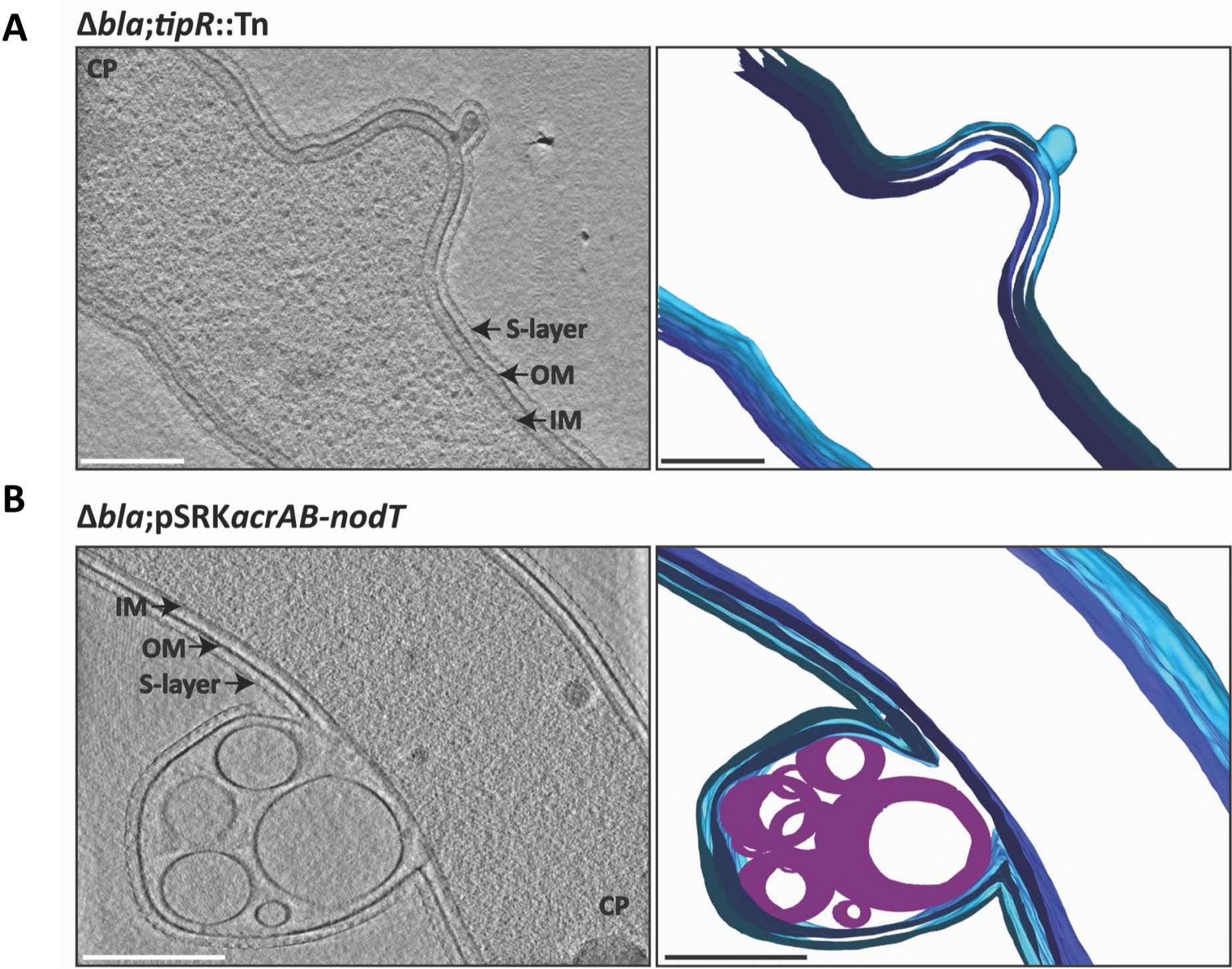

Supplement: S6 Fig — Representative cryo-tomographic slices (left) and 3D renderings (right) of C. crescentus Δbla;tipR::Tn (A) and Δbla;pSRK-acrAB-nodT (B) cells. Scale bar, 250 nm. CP, cytoplasm; IM (dark blue), inner membrane; OM (blue), outer membrane; S-Layer (cyan); vesicles (purple). (PDF) [file pbio.3002040.s006.pdf]

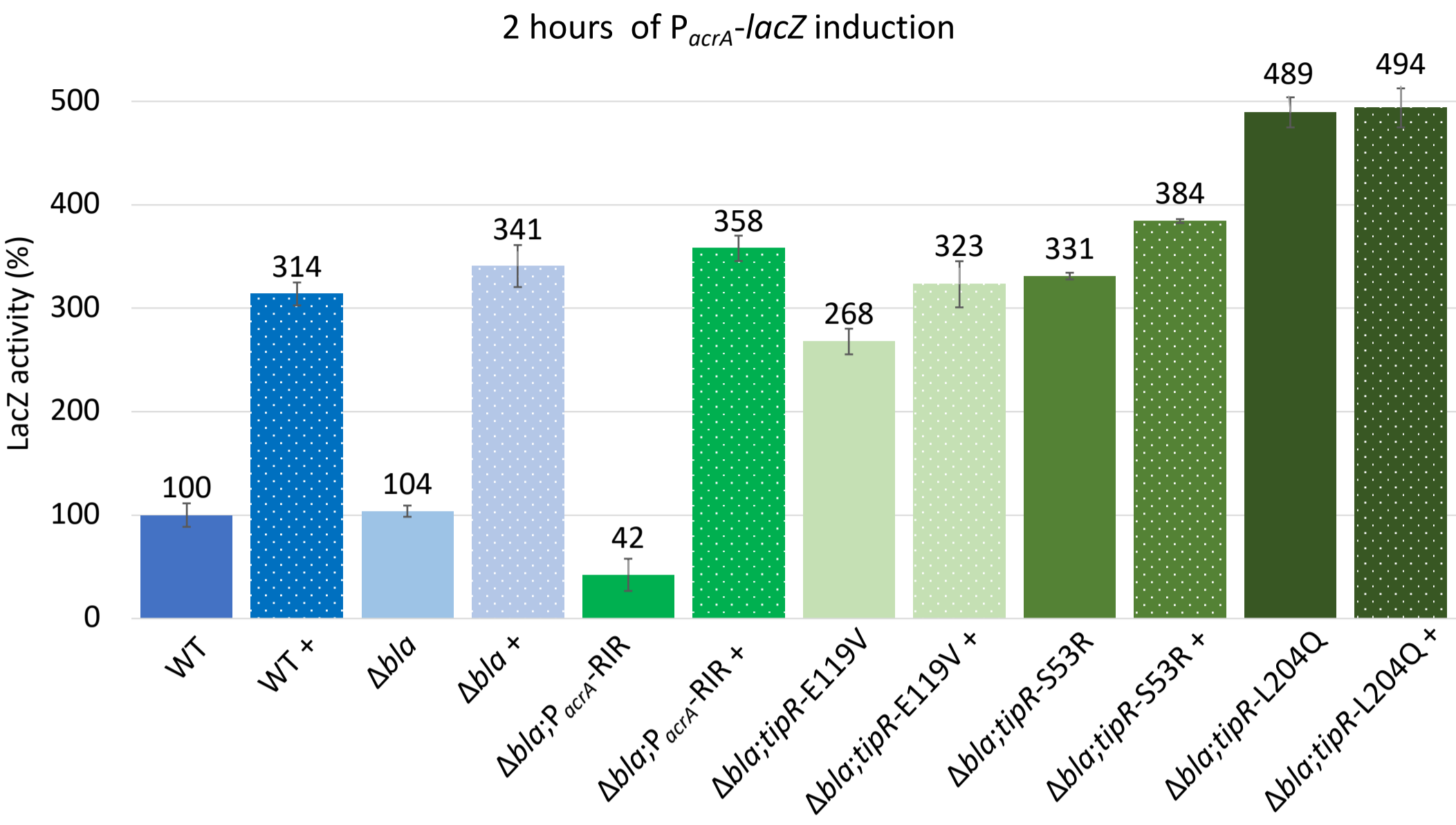

Supplement: S7 Fig — β-galactosidase activity expressed from PacrA-lacZ in various mutants. Induction (+) was for 2 hours with nalidixic acid (Nal, 10 μg/mL). All levels are indicated as percentage of expression regarding the basal level of the WT (NA1000) without induction. The data from the analysis are deposited in S2 Data. All strains carry additionally the pPacrA-lacZ promoter probe plasmid. (PDF) [file pbio.3002040.s007.pdf]

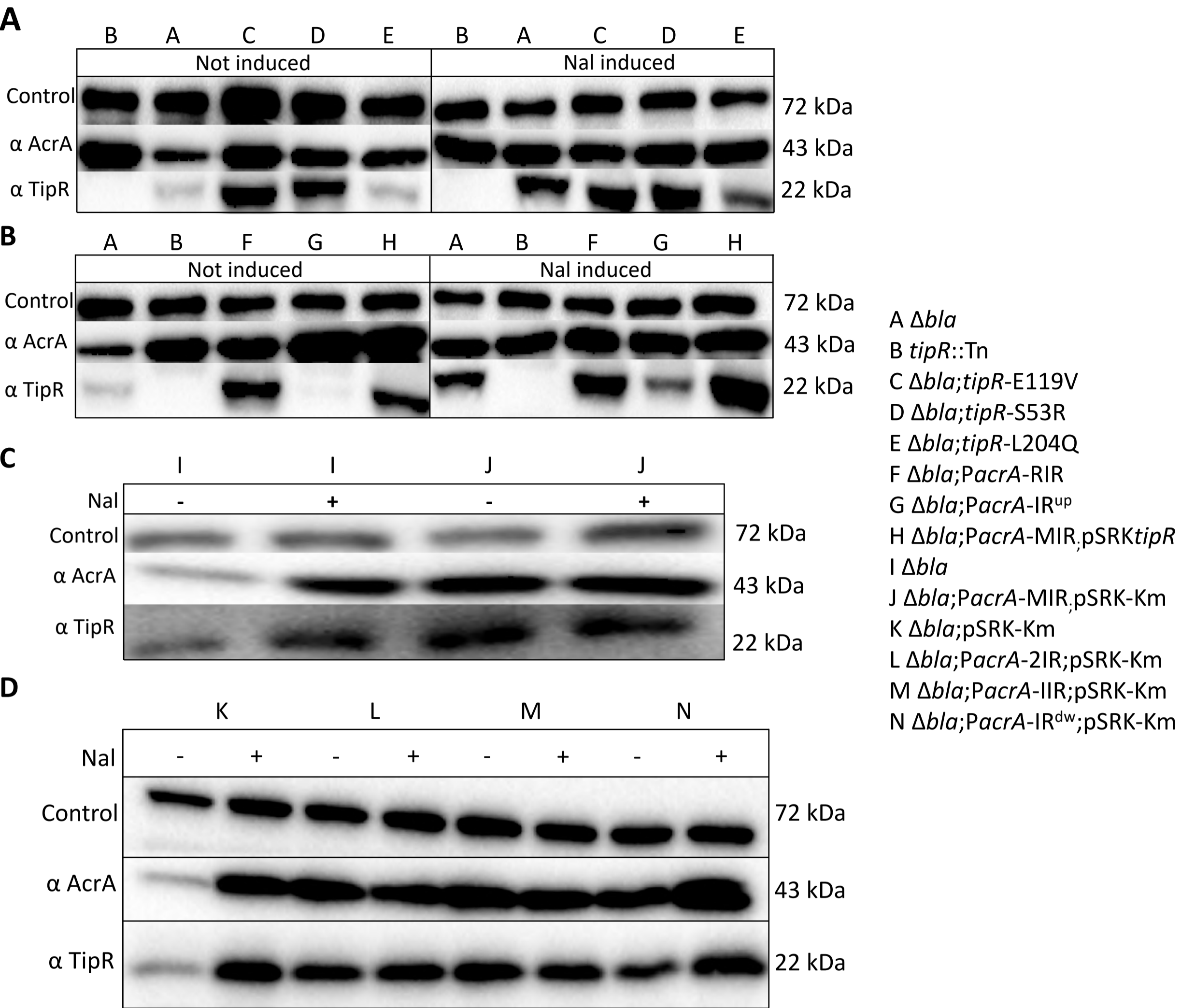

Supplement: S8 Fig — Immunoblots probed with polyclonal antibodies to AcrA and to TipR in extracts of different PacrA (B, C, and D) or tipR (A) mutants. All inductions (+) were performed after 2 hours of treatment with 10 μg/mL of Nal on exponentially grown cells in PYE. Blots were also probed with antibodies to CCNA_00163 as loading control. (PDF) [file pbio.3002040.s008.pdf]

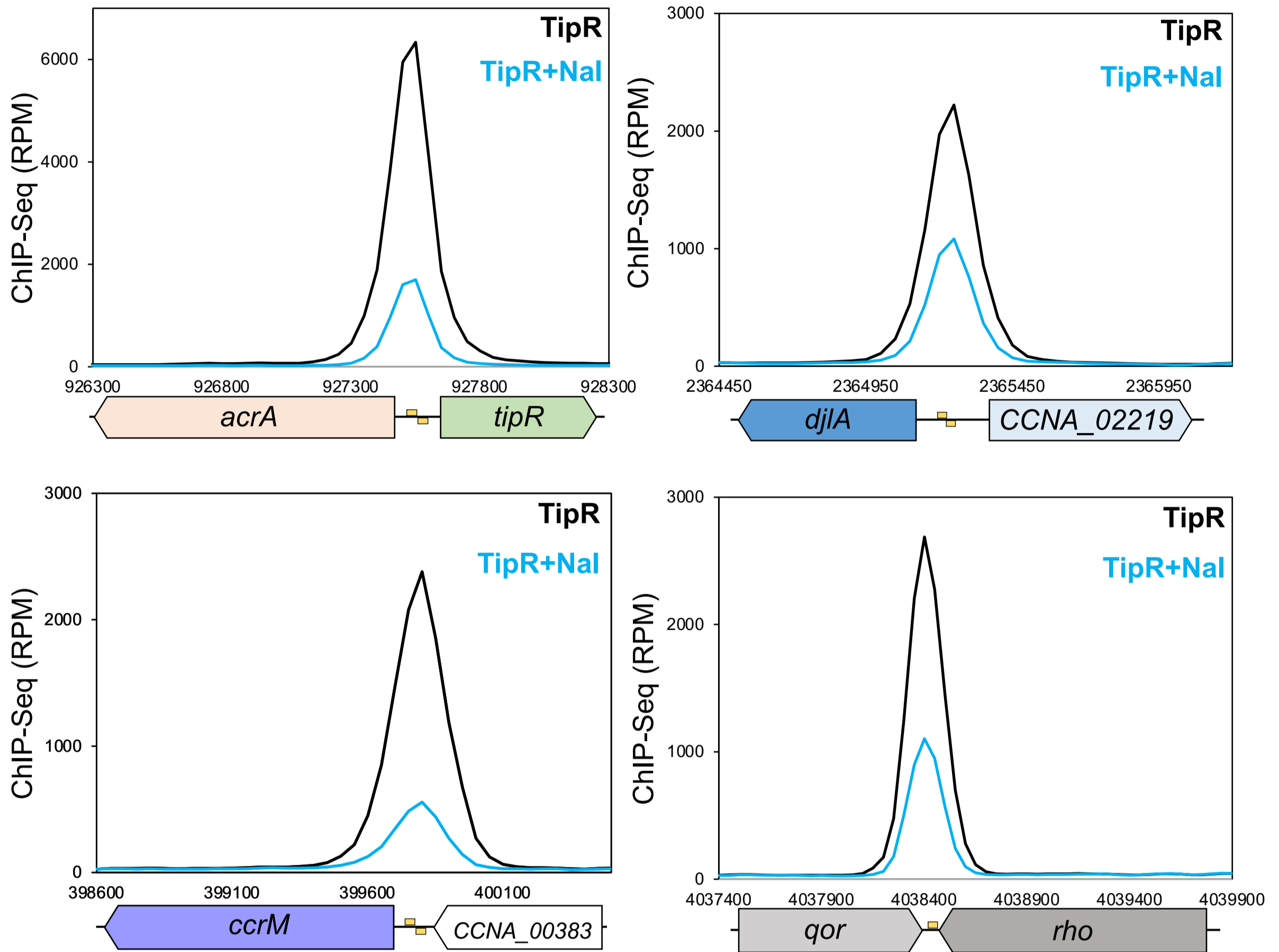

Supplement: S10 Fig — Representation of the reads [in reads per million (RPM)] obtained from the ChIP-Seq analyses covering the TipR binding regions. Positions are indicated under the graphic. Induction was performed for 30 minutes with Nal (20 μg/mL) in PYE (blue line) compared with the not induced condition (black line). The yellow boxes indicate the positions of the putative TipR binding site. The data from the analysis are deposited in S3 Data. (PDF) [file pbio.3002040.s010.pdf]

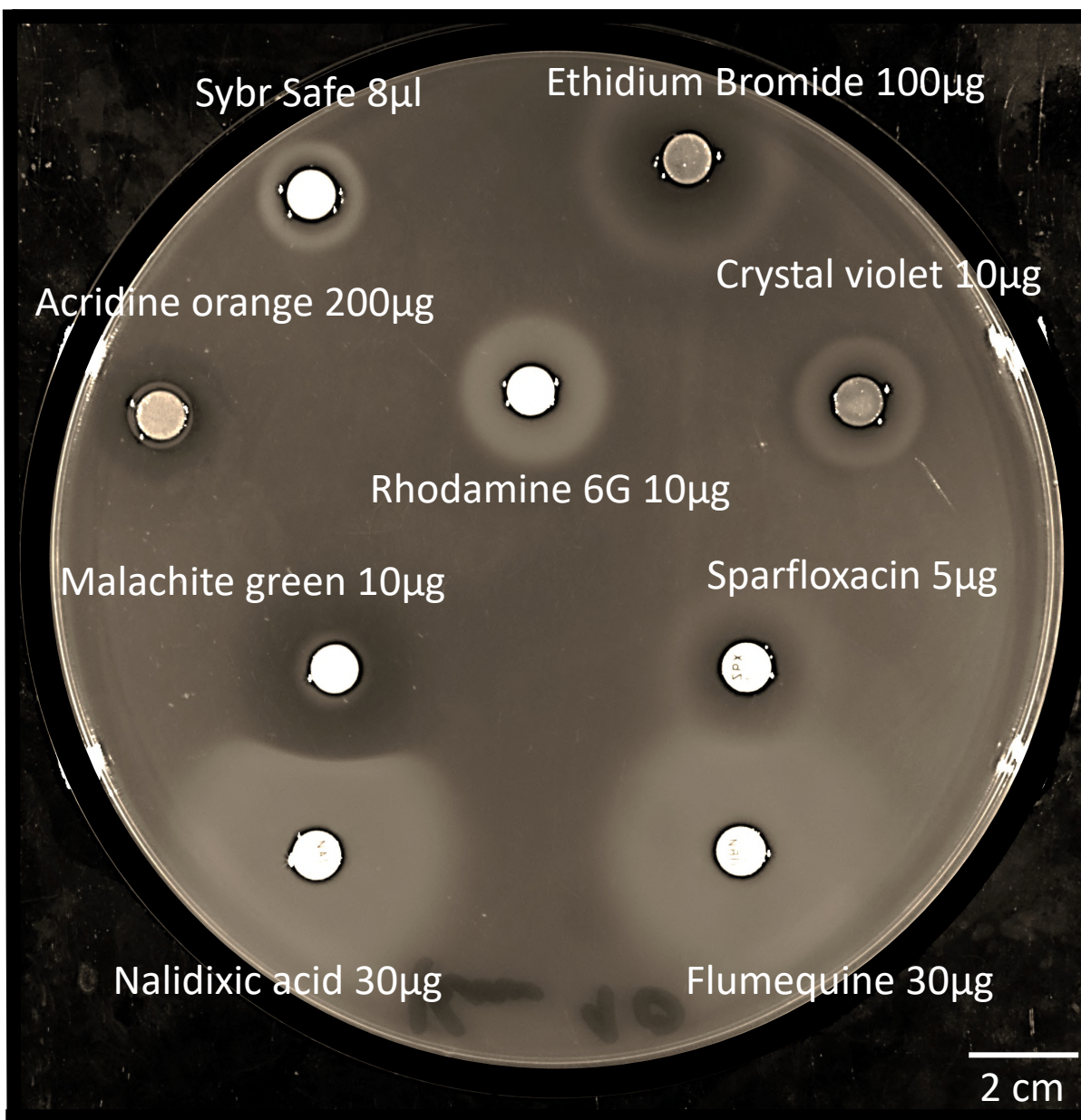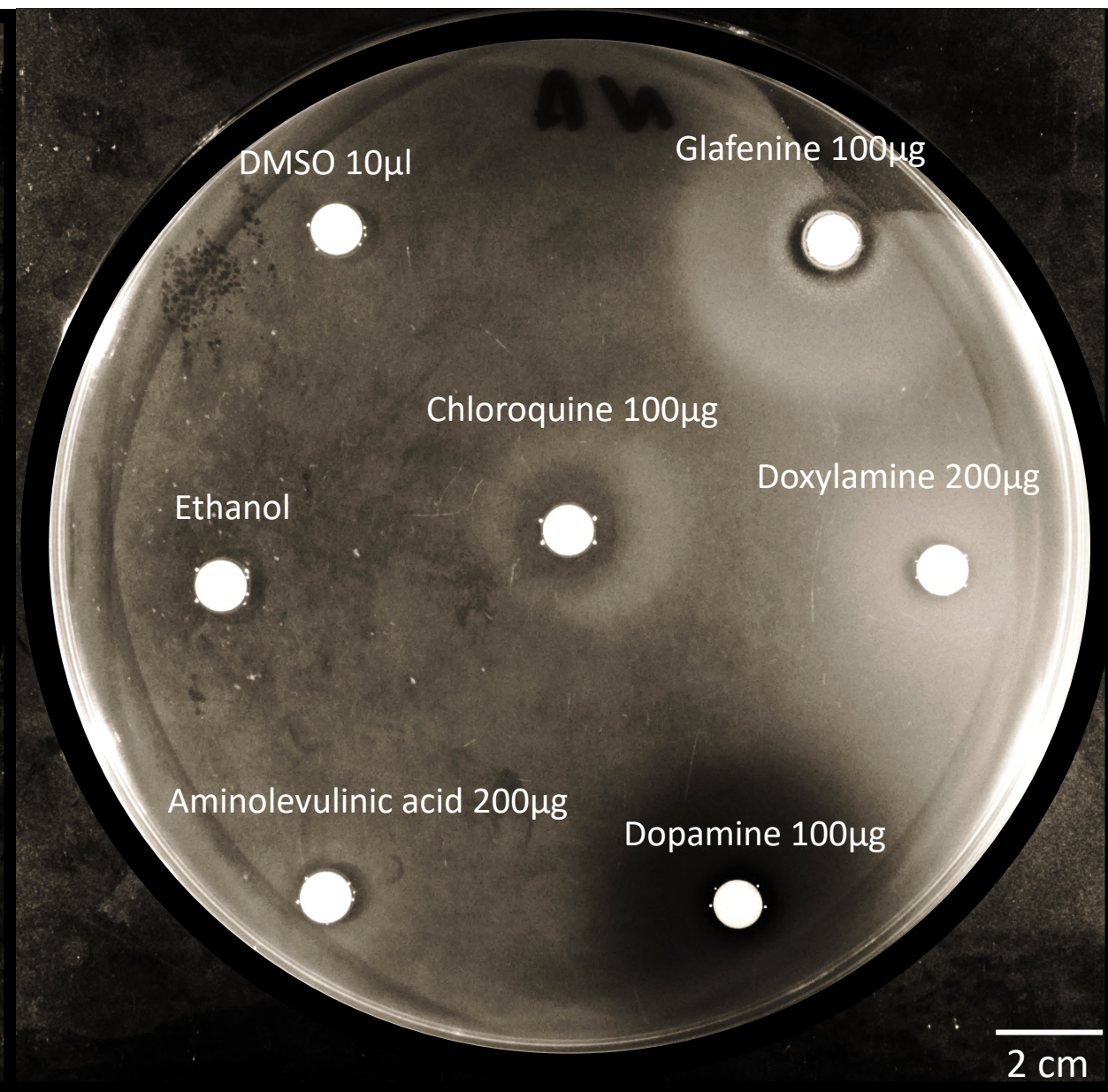

Supplement: S11 Fig — Reporter assay of PacrA activity fused with the nptII gene (PacrA-nptII conferring kanamycin resistance) cloned on plasmid plac290 (pPacrA-nptII). To identify inducers, chemicals were spotted on WT cells carrying the pPacrA-nptII reporter plasmid embedded on soft agar on PYE plates both containing with kanamycin (10 μg/mL). Plates were incubated for 2 days at 30°C. (PDF) [file pbio.3002040.s011.pdf]

Figure S12

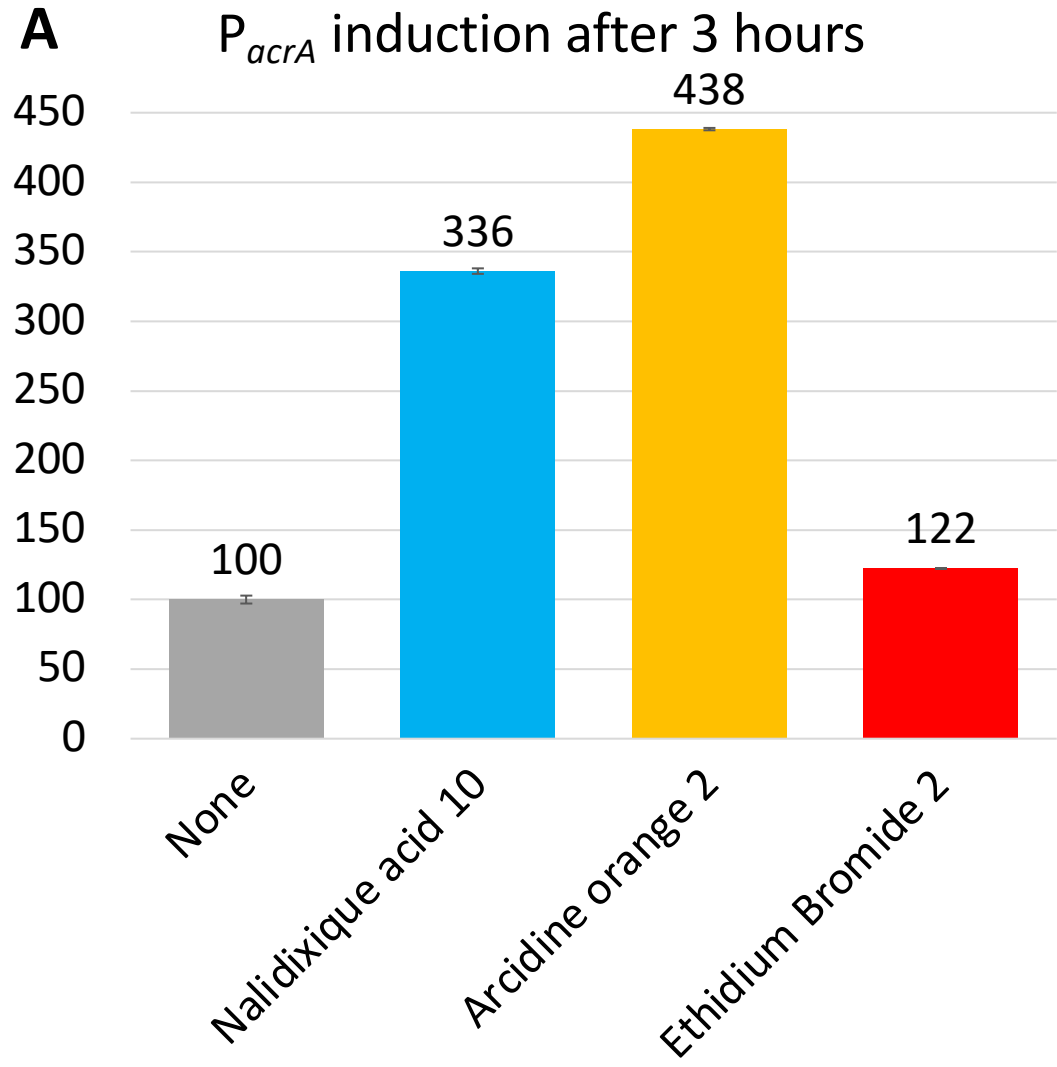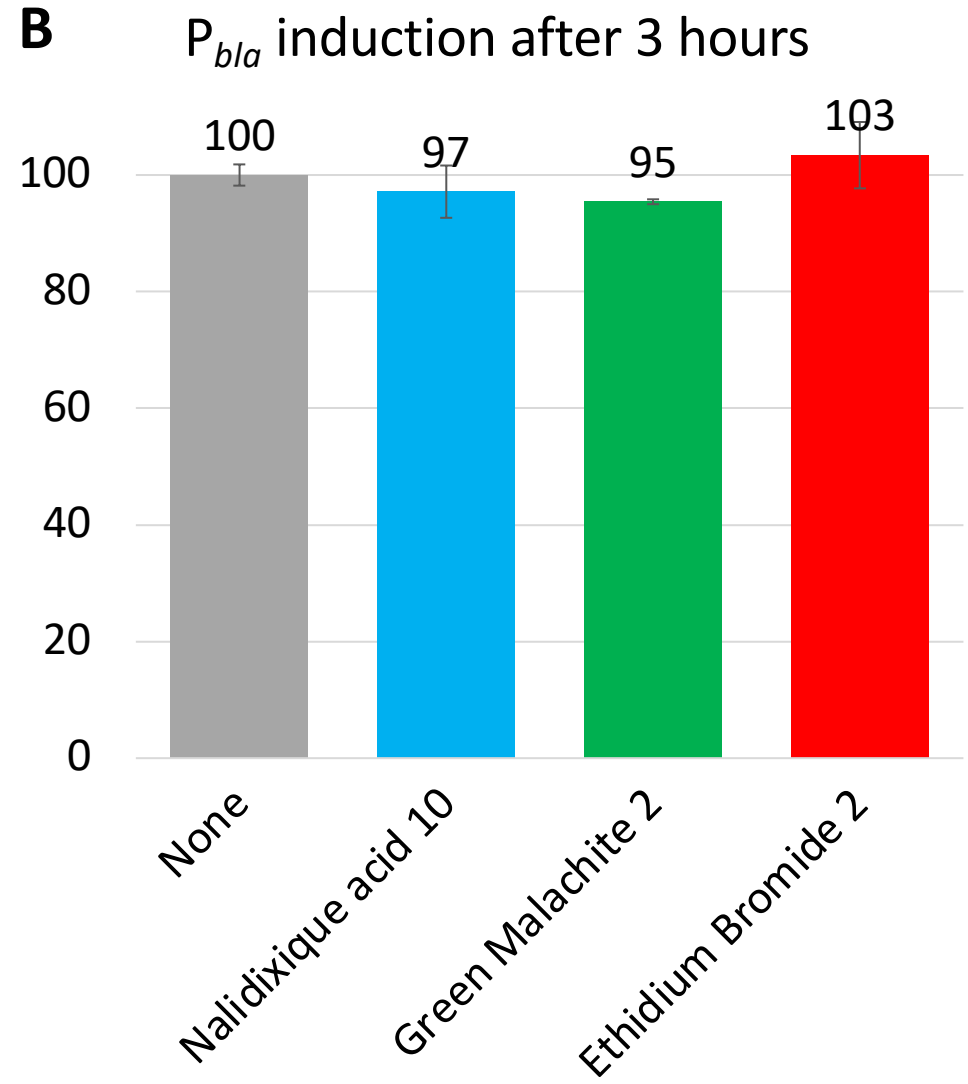

Supplement: S12 Fig — (A) β-galactosidase activity using the PacrA-lacZ in the ΔacrAB-nodT cells (A) or Pbla-lacZ in WT cells. (B) Inductions were performed for 3 hours. All levels are indicated in percentage of expression regarding the basal level of the uninduced state. The data from the analysis are deposited in S2 Data. (PDF) [file pbio.3002040.s012.pdf]

**A**

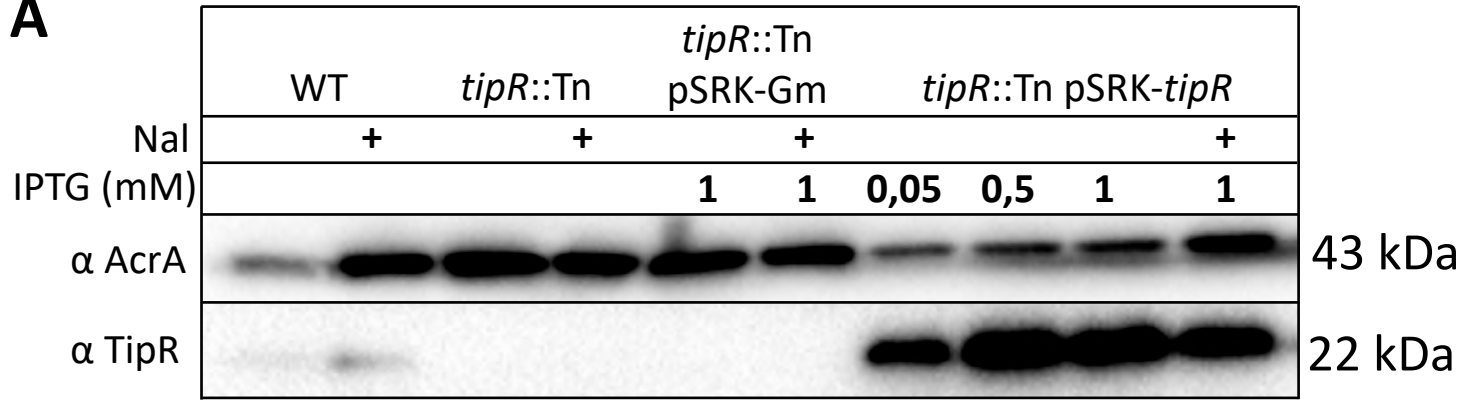

**B**

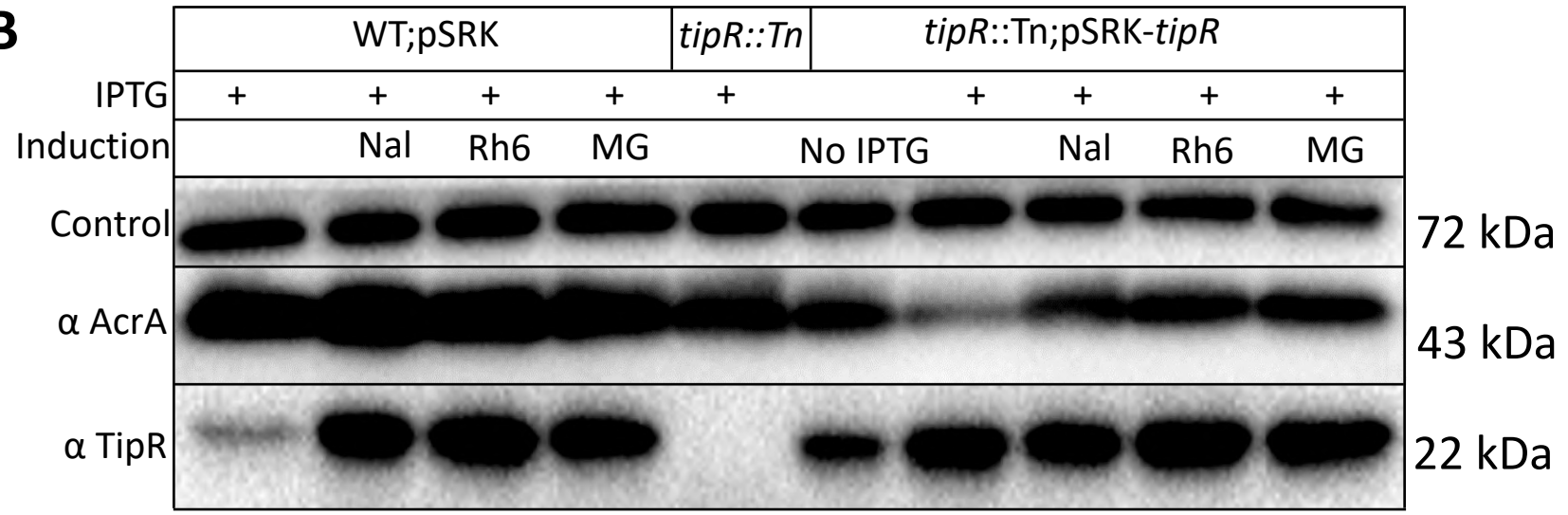

Supplement: S13 Fig — (A) Immunoblots probed with polyclonal antibodies to AcrA and to TipR to detect AcrA and TipR in tipR::Tn cells complemented with the pSRK-tipR plasmid, induced with the indicated amount of IPTG (in mM) and Nalidixic acid (Nal, 10 μg/mL) for 2 hours. (B) Immunoblot using polyclonal antibodies to AcrA and to TipR to probe extracts of multiple mutant strains grown in PYE with IPTG 0.5 mM, with and without 2 hours induction of Nal (10 μg/mL), rhodamine 6G (Rh6, 2 μg/mL), and malachite green (MG, 2 μg/mL). (PDF) [file pbio.3002040.s013.pdf]

Figure S14

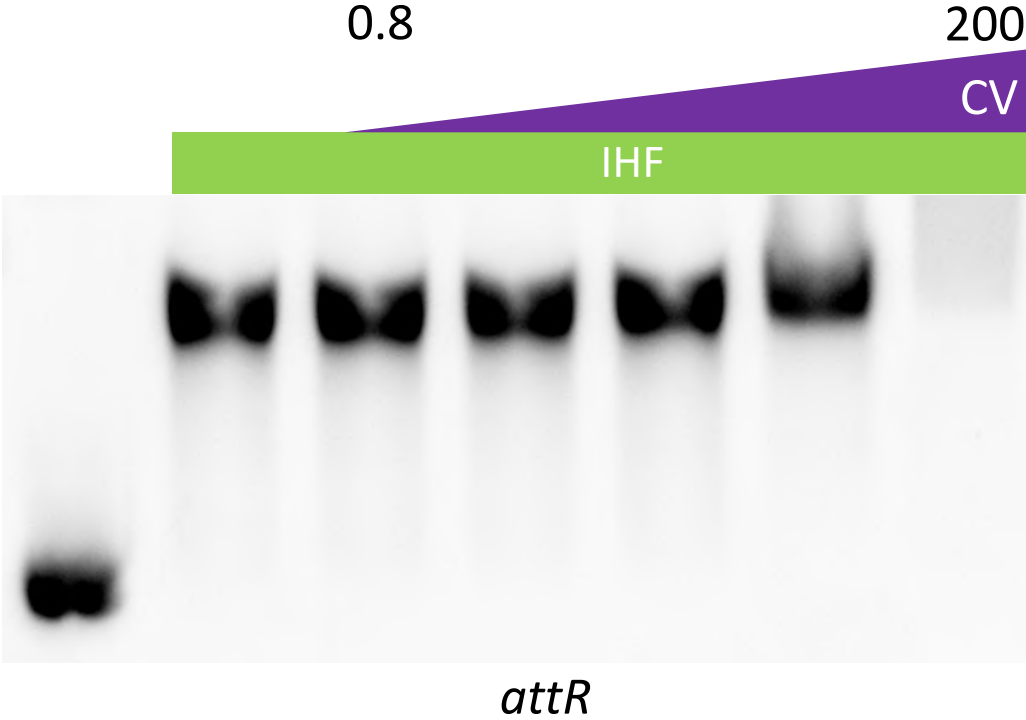

Supplement: S14 Fig — EMSA with 4 μM of IHF protein and 200 ng of Cy5-labelled attR DNA as probe. All quantities of crystal violet (CV) indicated are in μg/mL. (PDF) [file pbio.3002040.s014.pdf]

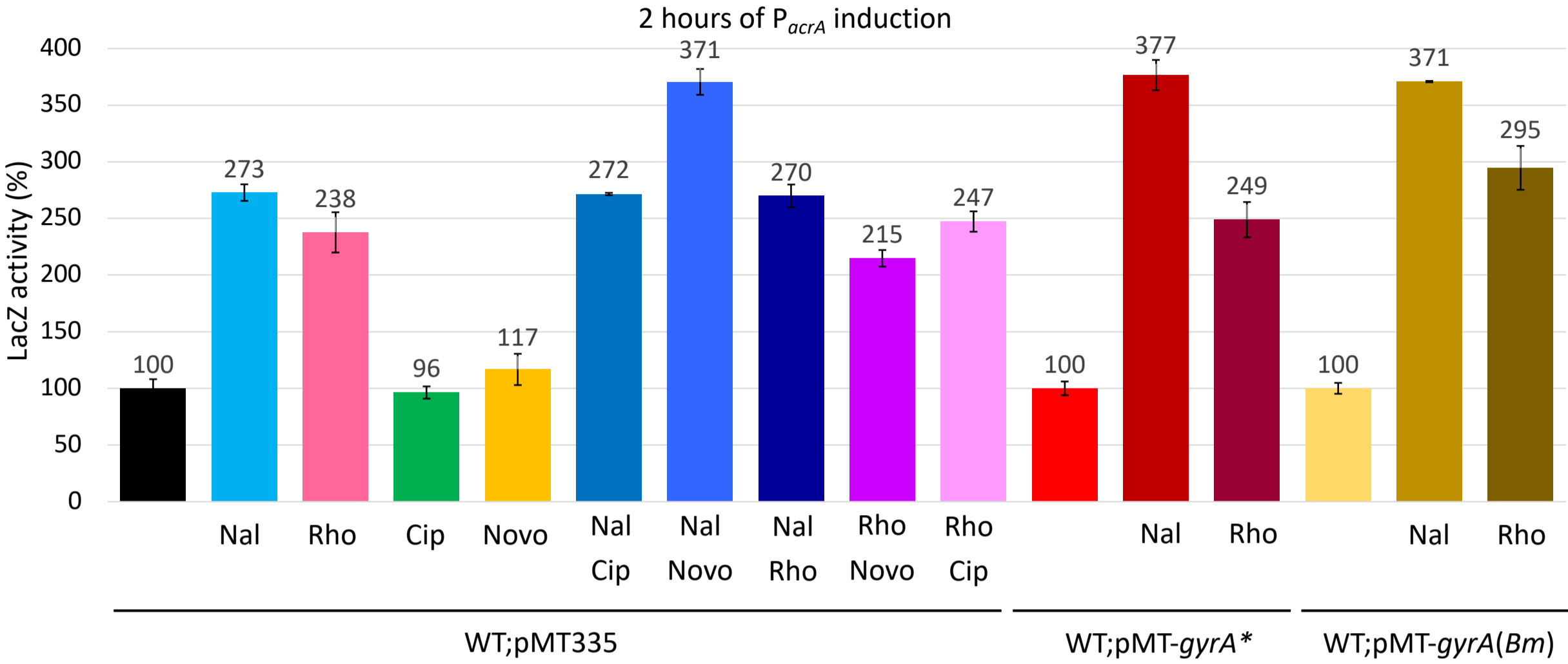

Supplement: S15 Fig — β-galactosidase activity measurements from PacrA-lacZ in NA1000 (WT) carrying the pMT335 or a derivative to express a gyrAF96N (GyrA*) or gyrA from Brucella melitensis (Bm) grown in PYE Van 50 μM. Antibiotics were used at the following concentrations: nalidixic acid (Nal, 10 μg/mL), ciprofloxacin (Cip, 2 μg/mL), rhodamine 6G (Rho, 2 μg/mL), novobiocin (Novo, 10 μg/mL). All levels are indicated in percentage of expression regarding the basal level of WT before induction. The data from the analysis can be found in S2 Data. (PDF) [file pbio.3002040.s015.pdf]

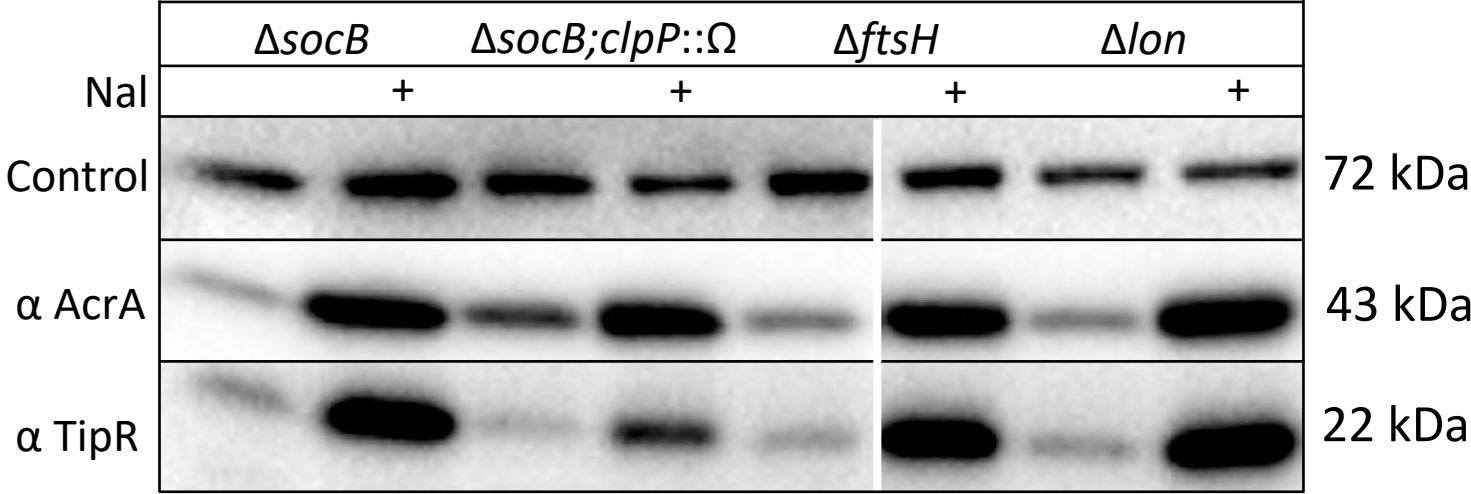

Supplement: S16 Fig — Immunoblot analysis using polyclonal antibodies to AcrA and to TipR to determine the steady-state levels of AcrA and TipR in various protease mutants of C. crescentus, before and after induction with nalidixic acid (Nal, +, 10 μg/mL) for 2 hours in PYE. Loading control is anti-CCNA_00164. All samples were loaded on the same immunoblot. (PDF) [file pbio.3002040.s016.pdf]

Figure S17

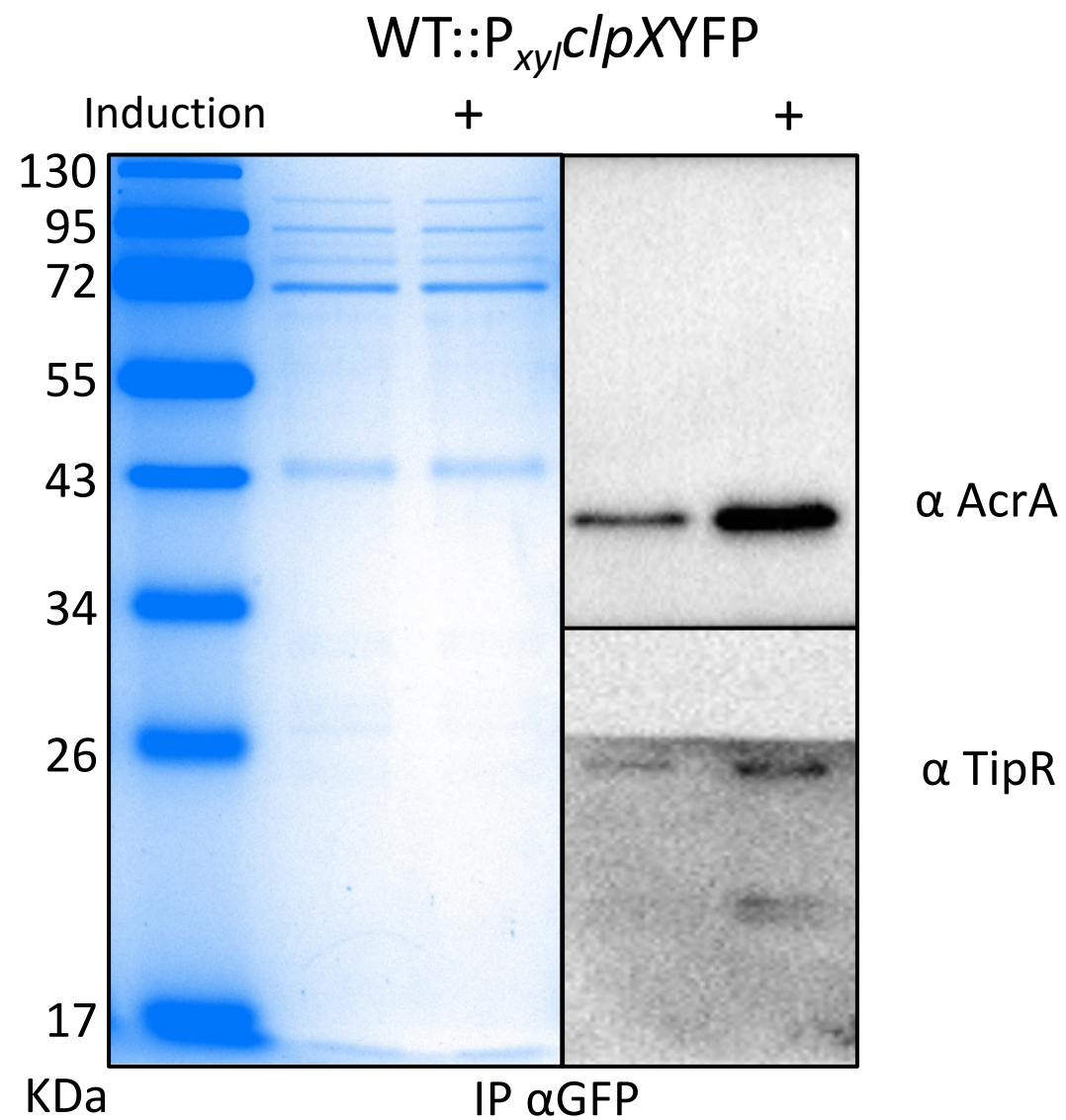

Supplement: S17 Fig — Coomassie Blue–stained PAGE (12% gel) (left) and immunoblot (right) of a ClpX-YFP co-immunoprecipitation (GFP Trap Matrix) with polyclonal antibodies to TipR and to AcrA. Induction with (+) or without (−) Nal (10 μg/mL). (PDF) [file pbio.3002040.s017.pdf]

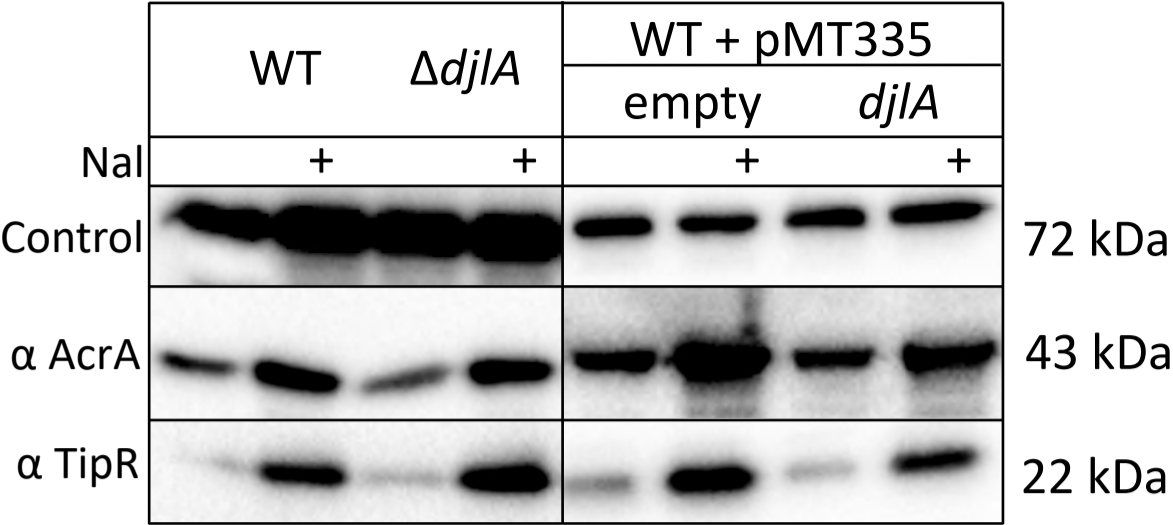

Supplement: S18 Fig — Immunoblot analysis of extracts from WT and co-chaperone mutants using polyclonal antibodies to TipR and to AcrA. All inductions (+) were performed for 2 hours with 10 μg/mL of nalidixic acid (Nal). Strains carrying the pMT335 are induced with vanillate 100 μM (Van). Immunoblots performed with antibodies to CCNA_00163 serve as loading controls. (PDF) [file pbio.3002040.s018.pdf]

**A**

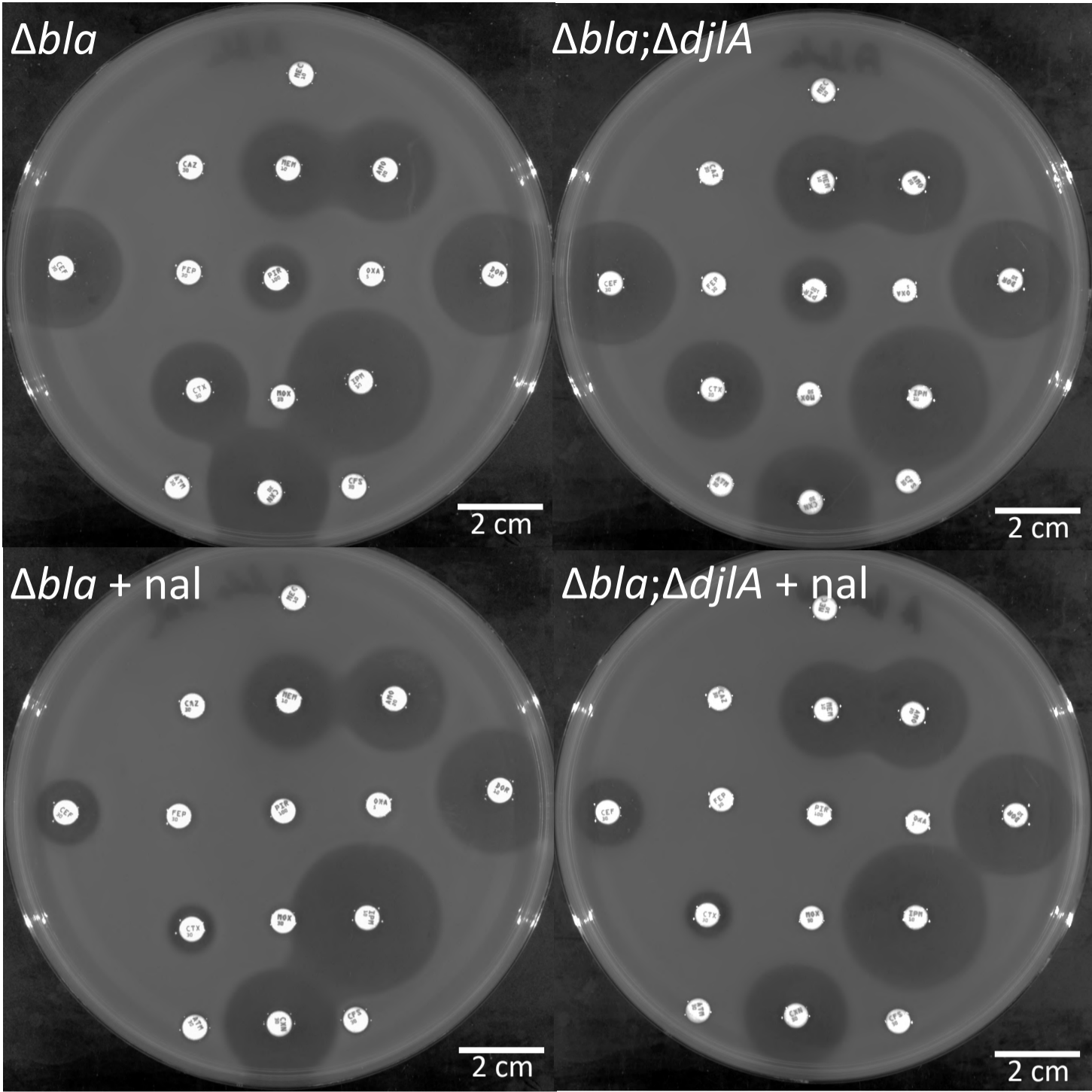

**B**

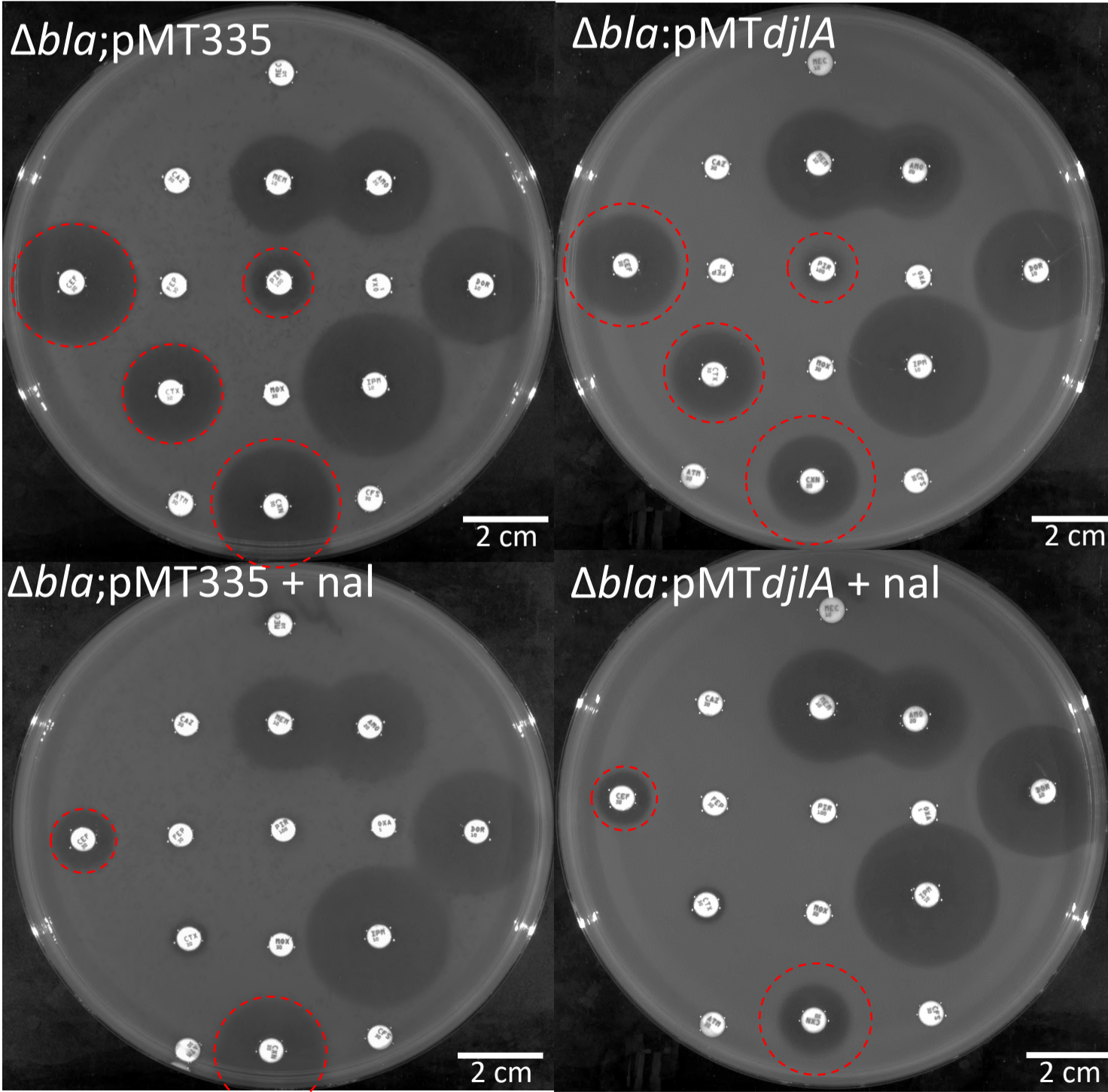

Supplement: S19 Fig — Antibiograms of C. crescentus strains using antibiotic discs, from top left to bottom right, Mecillinam 10 μg, Ceftazidime 40 μg, Meropenem 10 μg, Amoxicillin 20 μg, Cephalothin 30 μg, Cefepime 30 μg, Piperacillin 100 μg, Oxacillin 5 μg, Doripenem 10 μg, Cefotaxime 30 μg, Moxalactam 30 μg, Imipenem 10 μg, Aztreonam 30 μg, Cephalexin 40 μg, Cefsulodin 30 μg. Nal induction performed at 10 μg/mL. All plates with strains carrying a pMT335 or pMT335-djlA are supplemented with vanillate 100 μM. (PDF) [file pbio.3002040.s019.pdf]

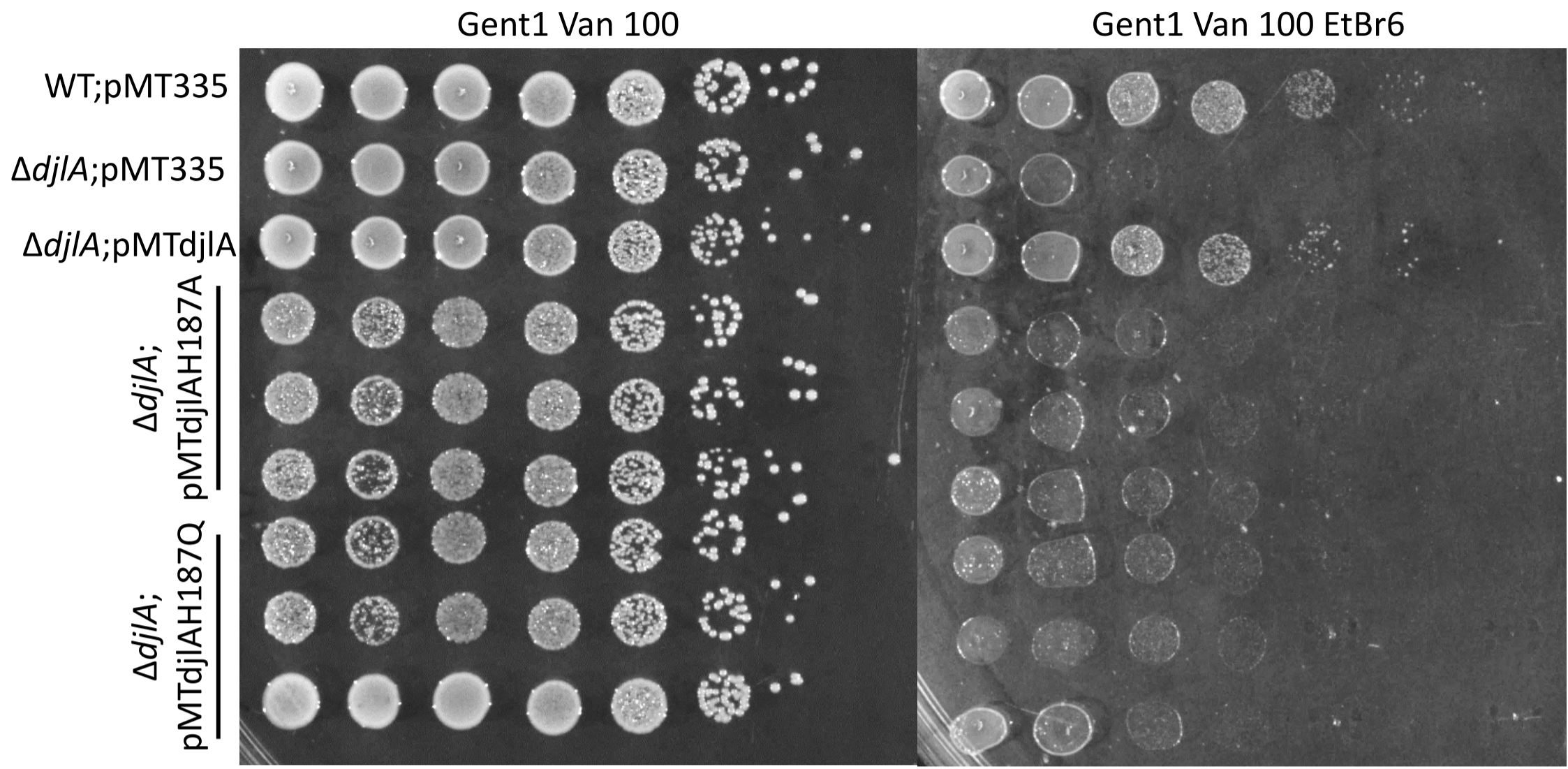

Supplement: S20 Fig — Efficiency of plating (EOP) assay determined by 10-fold serial dilutions of ΔdjlA mutant on plates containing ethidium bromide (EtBr, 6 μg/mL) to probe for efflux pump activity. All strains contain pMT335 plasmid or a derivative (pMT335-djlA, pMT335-djlAH187A, and pMT335-djlAH187Q) expressing DjlA, grown on vanillate (Van) at 100 μM and gentamicin (Gent) at 1 μg/mL. (PDF) [file pbio.3002040.s020.pdf]

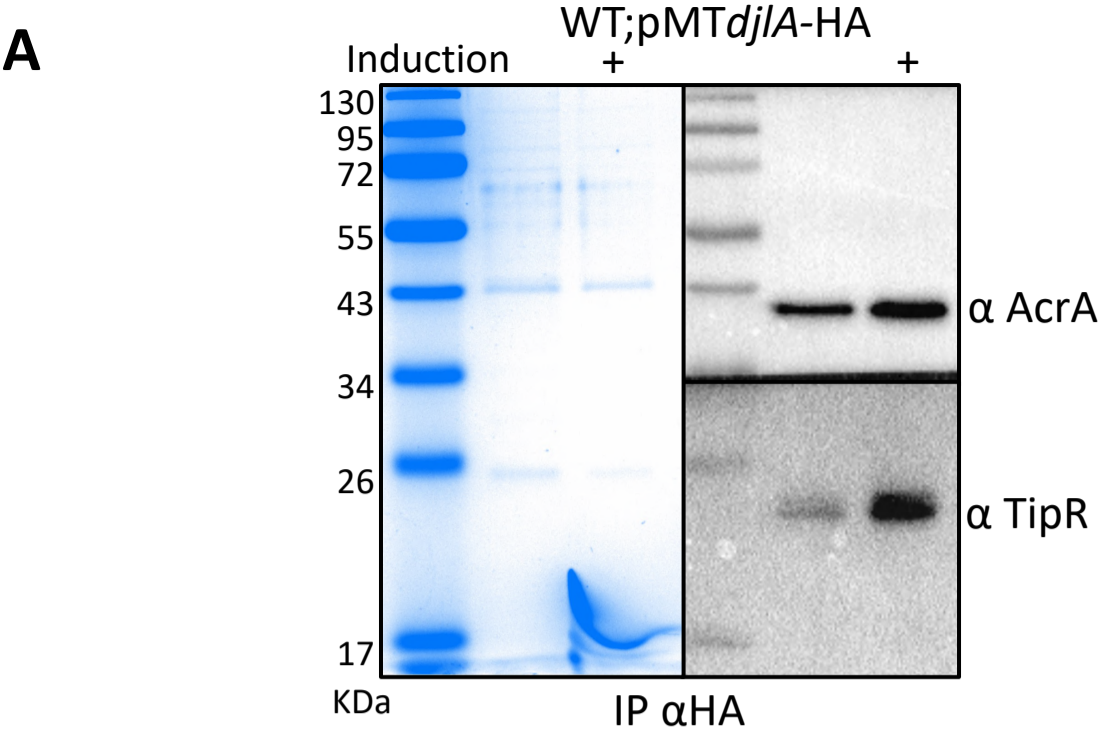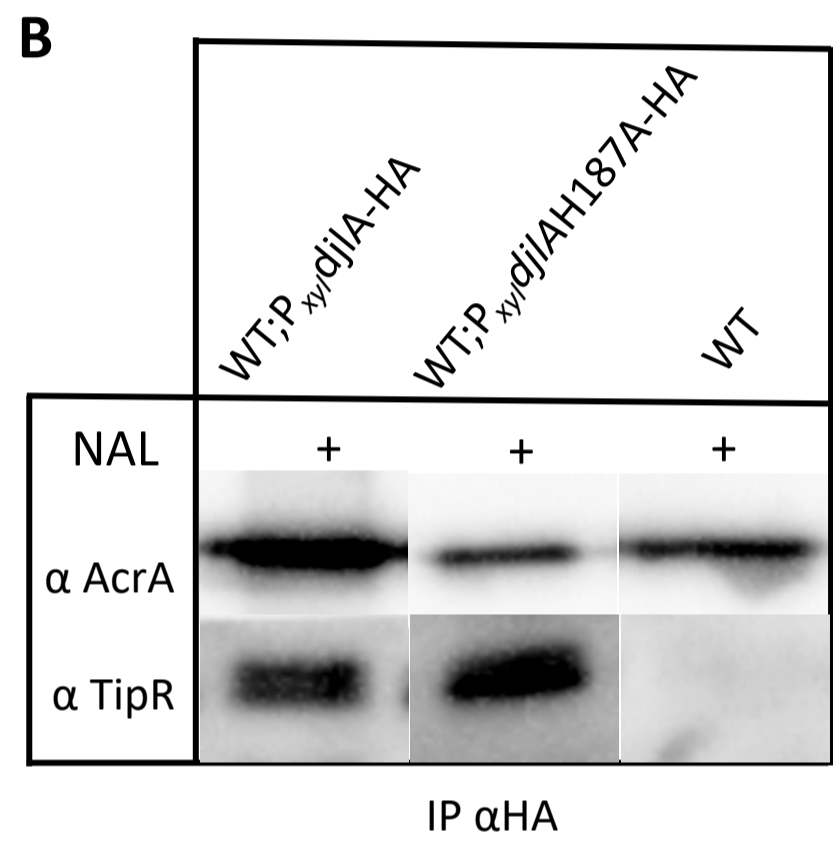

Supplement: S21 Fig — (A) Coomassie staining after 12% PAGE (left) and immunoblotting (right) of a DjlA-HA co-immunoprecipitation eluate (from anti HA affinity matrix) probed with polyclonal antibodies to AcrA and to TipR. Induction was with Nal (+) at 10 μg/mL for 2 hours. (B) Immunoblots probed with polyclonal antibodies to TipR and to AcrA after pull-down of HA-tagged protein from extracts of cells expressing DjlA-HA, DjlAH187A-HA, DjlAH187Q-HA, DnaJ1-HA, and DnaJ2-HA using the anti HA affinity matrix. Induction was with Nal (+) at 10 μg/mL for 2 hours. (PDF) [file pbio.3002040.s021.pdf]

A

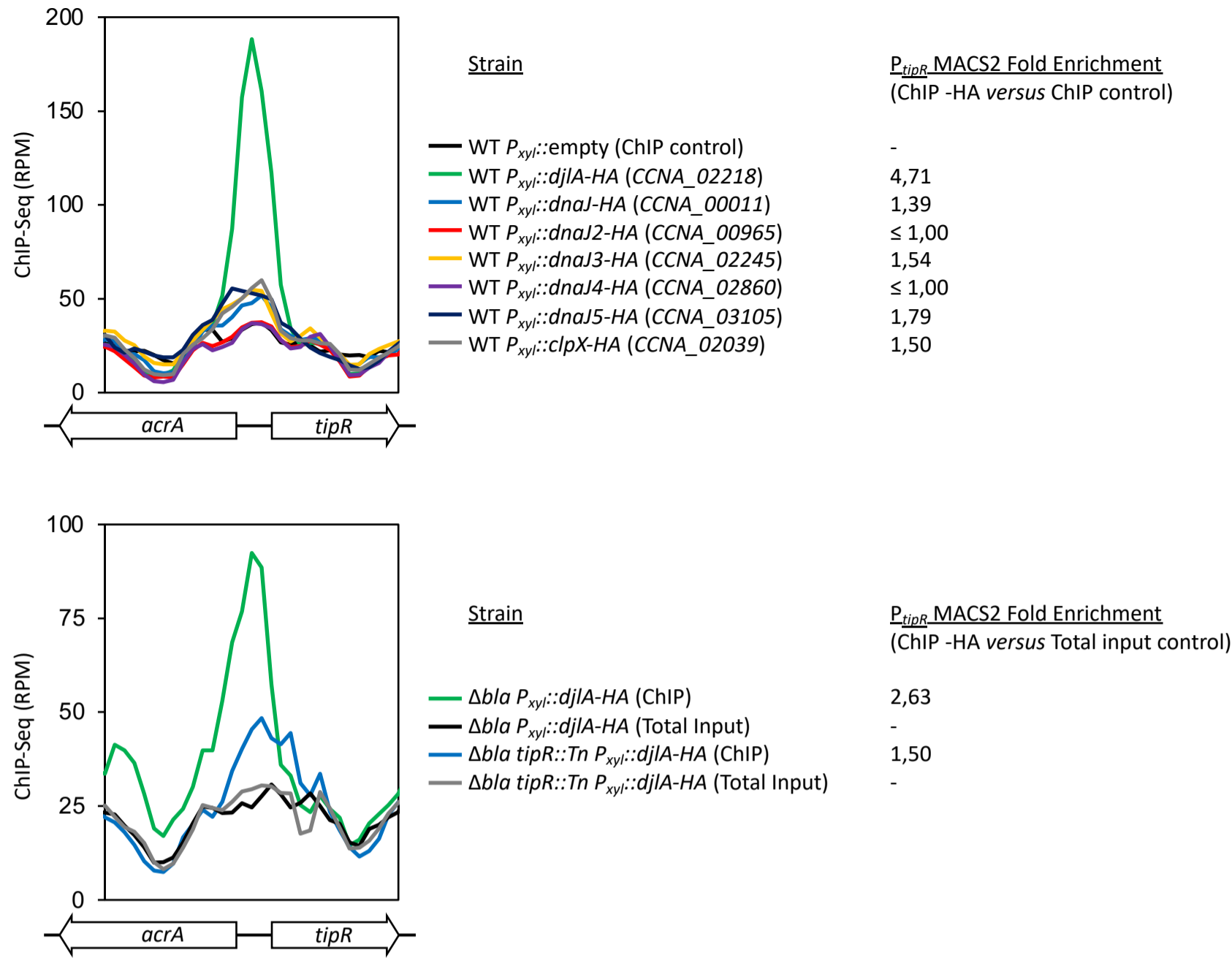

B

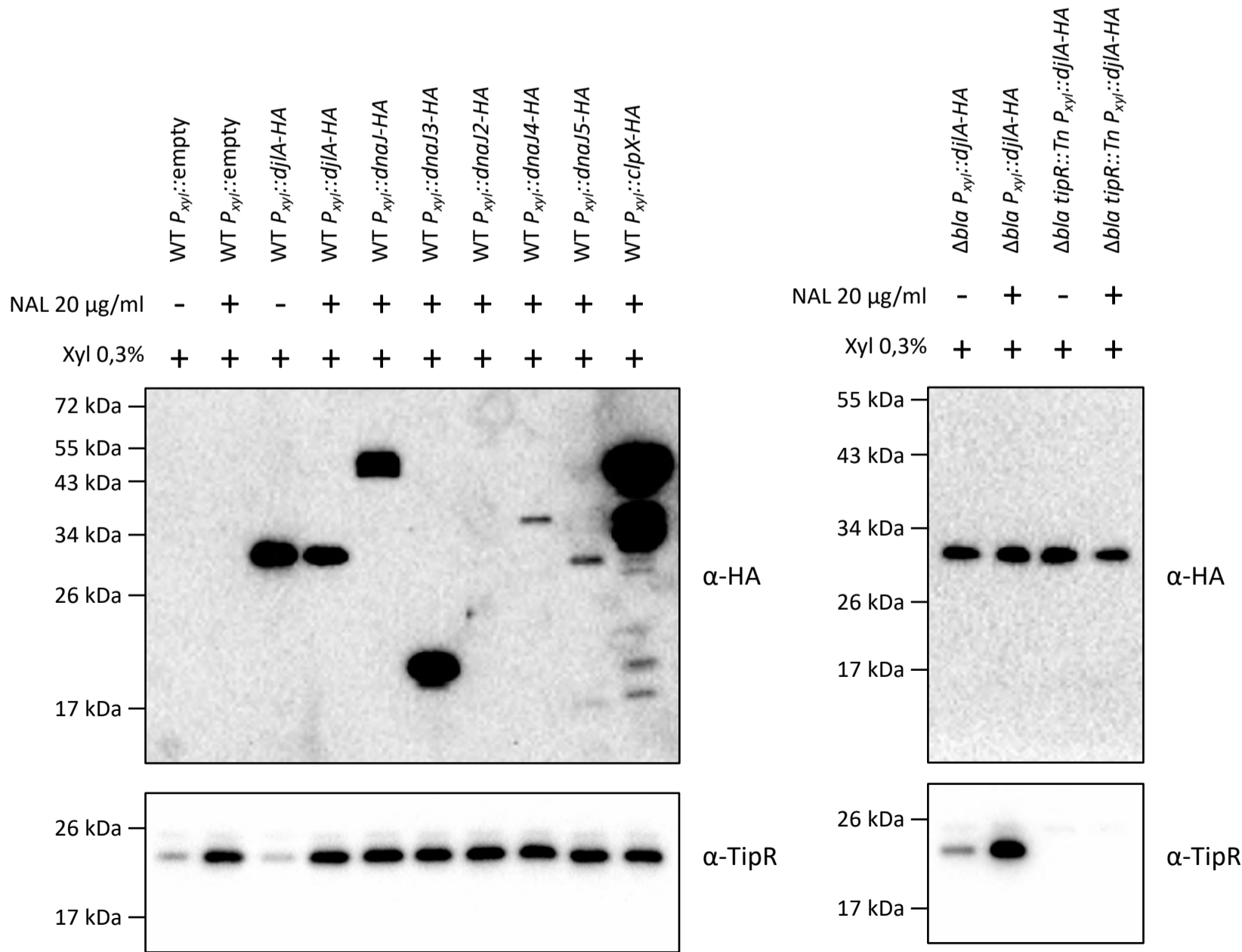

Supplement: S22 Fig — (A) Representation of the reads (in reads per million (RPM)) obtained from the ChIP-Seq analyses covering the TipR binding regions. Positions are indicated under the graphic. Induction was performed for 30 minutes with Nalidixic acid (Nal) 20 μg/mL in PYE (blue line) compared with the not induced condition (black line). The data from the analysis are deposited in S23 Fig. (B) Immunoblotting of extracts from cells expressing DjlA-HA, DnaJ1 to 5-HA and ClpX-HA used in the ChIP-Seq experiment. The blots were probed with polyclonal antibodies to TipR and monoclonal antibodies to the HA tag. (PDF) [file pbio.3002040.s022.pdf]

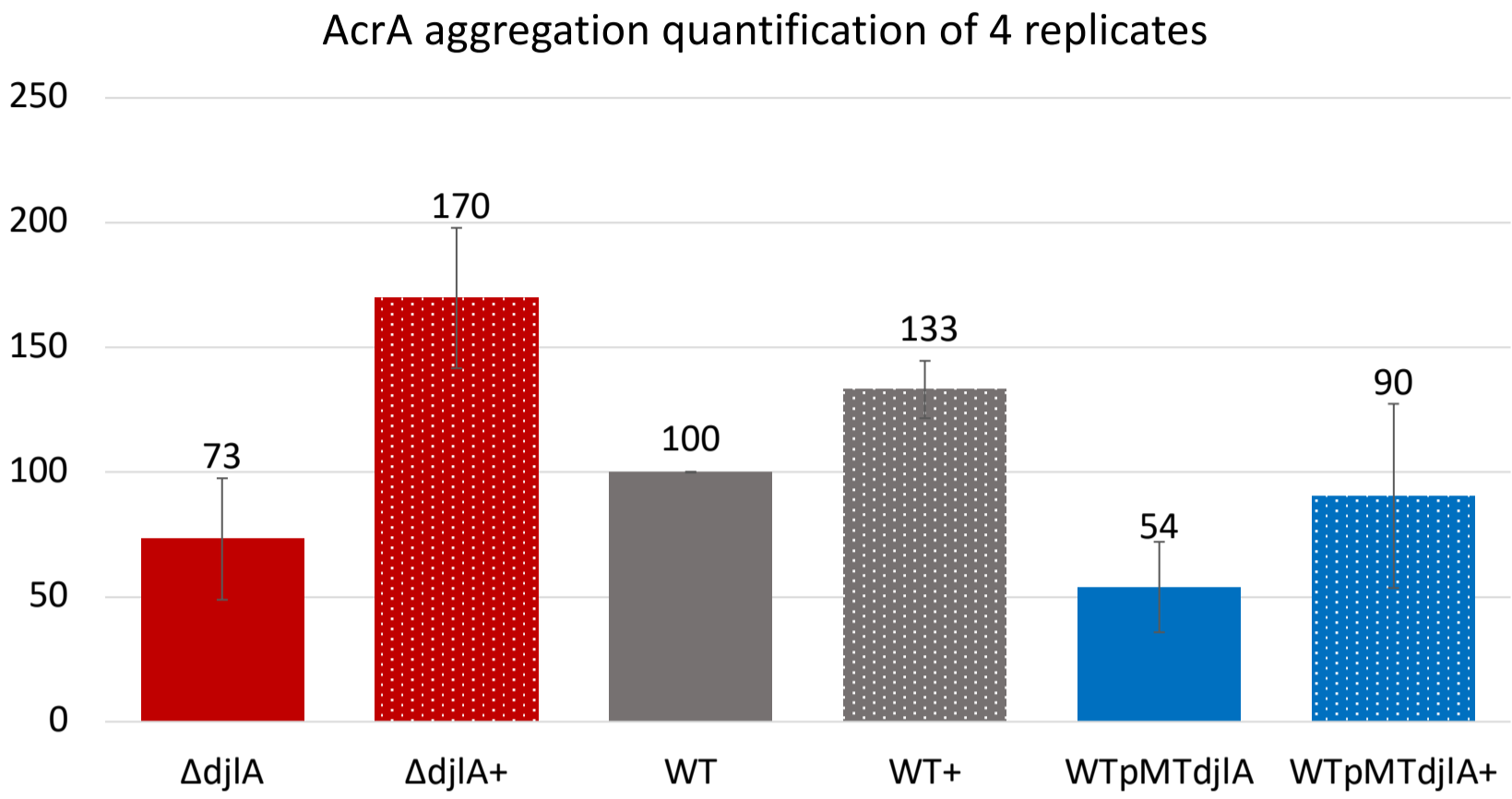

Supplement: S23 Fig — Graphical representation of 4 replicates of aggregation of assay quantified with ImageJ from immunoblotting of AcrA in soluble versus insoluble cell lysates as determined by immunoblotting using polyclonal antibodies to AcrA. All inductions (+) were performed for 2 hours with 10 μg/mL of Nal. Strains carrying the pMT335 are induced with vanillate (Van, 100 μM). All samples are normalized regarding the quantification of uninduced WT set at 100%. The data from the analysis are deposited in S5 Data. (PDF) [file pbio.3002040.s023.pdf]

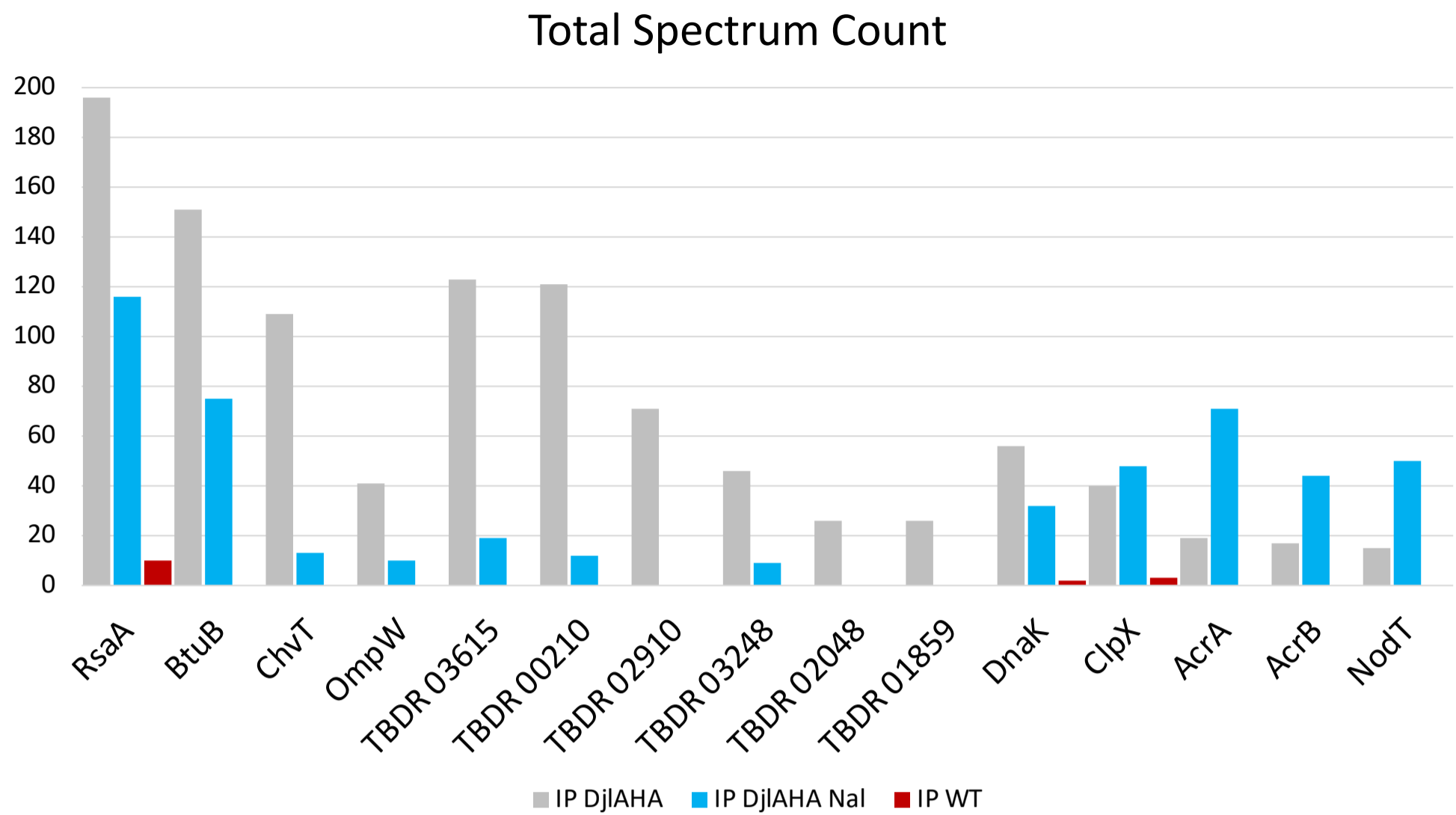

Supplement: S24 Fig — Graphical representation of the total spectrum count of various proteins detected by LC-MS/MS upon immunoprecipitation of DjlA-HA from soluble cell lysates using anti-HA affinity matrix. The lysates used were from WT expressing DjlA-HA plus (blue) and minus (grey) NAL, and a WT control without DjlA-HA (red). All the TonB-dependant receptors (TBDRs) are annotated with their respective CCNA_#. The data from the LC-MS/MS analysis are deposited in S5 Data. (PDF) [file pbio.3002040.s024.pdf]
