# Supplementary material for: Adaptive β-lactam resistance from an inducible efflux pump that is post-translationally regulated by the DjlA co-chaperone
Source: PLoS Biol. 2023 Dec 5;21(12):e3002040. doi: 10.1371/journal.pbio.3002040 (PMC10754441; doi:10.1371/journal.pbio.3002040)
Supplement: S9 Fig — An inverted repeat (IR) sequences was detected at each of the 3 TipR-binding positions on the chromosome, plus a half-site at the fourth target site. Consensus sequence of the IRs based on the sequences detected at position 399827, 927547, and 2365267 on the chromosome. This consensus has been identified using MEME software (mean P value: 4.48 × 10^−6^) and drawn by WebLogo (crooks). (PDF) [file pbio.3002040.s009.pdf]

Figure S9

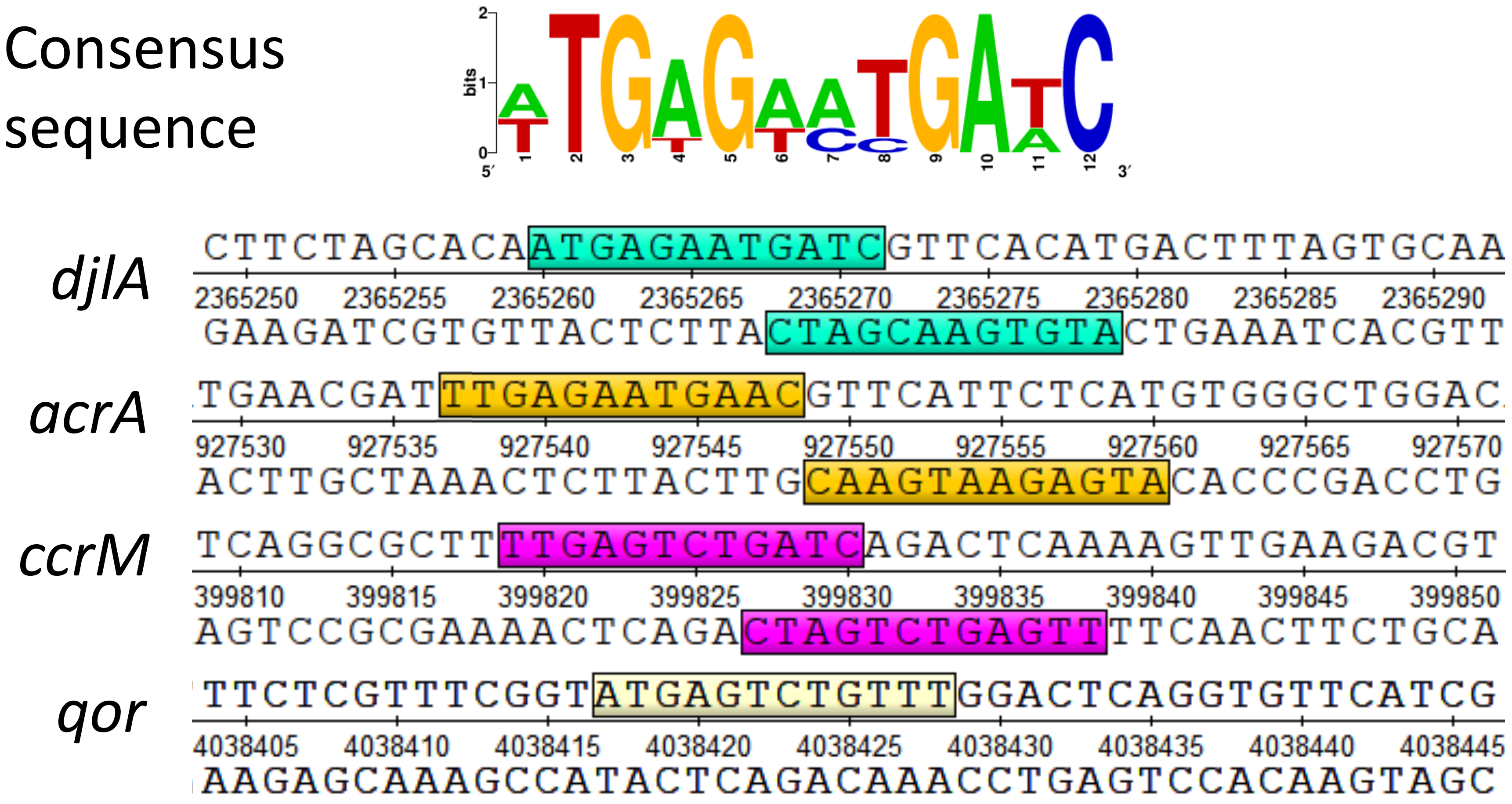
Consensus sequence
djlA
CTTCTAGCACAATGAGAATGATCGTTCACATGACTTTAGTGCAA
2365250 2365255 2365260 2365265 2365270 2365275 2365280 2365285 2365290
GAAGATCGTGTTACTCTTACTAGCAAGTGTACTGAAATCACGTT
acrA
TGAACGATTTGAGAATGAACGTTCATTCTCATGTGGGCTGGAC
927530 927535 927540 927545 927550 927555 927560 927565 927570
ACTTGCTAAACTCTTACTTGCAAGTAAGAGTACACCCGACCTG
ccrM
TCAGGCGCTTTTGAGTCTGATCAGACTCAAAAGTTGAAGACGT
399810 399815 399820 399825 399830 399835 399840 399845 399850
AGTCCGCGAAAACTCAGACTAGTCTGAGTTTTCAACTTCTGCA
qor
TTCTCGTTTCGGTATGAGTCTGTTTGGACTCAGGTGTTCATCG
4038405 4038410 4038415 4038420 4038425 4038430 4038435 4038440 4038445
AAGAGCAAAGCCATACTCAGACAAACCTGAGTCCACAAGTAGC
