# Supplementary material for: Adaptive β-lactam resistance from an inducible efflux pump that is post-translationally regulated by the DjlA co-chaperone
Source: PLoS Biol. 2023 Dec 5;21(12):e3002040. doi: 10.1371/journal.pbio.3002040 (PMC10754441; doi:10.1371/journal.pbio.3002040)
Supplement: S6 Data — (DOCX) [file pbio.3002040.s030.docx]

**S6 Data. Strains, plasmids, primers and synthetic genes used in this study:**

| **Name** | **Description** | **Origin** |
| --- | --- | --- |
| *Caulobacter crescentus* | | |
| NA1000 | Synchronizable variant of strain CB15 | Evinger & Agabian 1977 |
| Δ*bla* | Full deletion of the metallo-β-lactamase (CCNA_02223) | West & Stephens 2002 |
| *tipR*::Tn | Himar transposon inserted in *tipR* (from pHPV414) obtained on piperacillin 40µg/ml in a Δ*bla* and transduced in WT | This work |
| Δ*acrAB-nodT* | Complete deletion of the *acrAB-nodT* operon | Kirkpatrick et al. 2014 |
| Δ*tipR* Δ*acrAB-nodT* | Complete deletion of the *acrAB-nodT* operon and *tipR* | Kirkpatrick et al. 2014 |
| Δ*bla* P*_acrA_*-R*IR* | Suppressor strain obtained on cephalotin 10µg/ml with mutation in inverted repeat of the *acrA* promoting region | This work |
| Δ*bla* P*_acrA_*-IR^up^ | Suppressor strain obtained on cephalotin 10µg/ml with mutation upstream of inverted repeat of the *acrA* promoting region | This work |
| Δ*bla* P*acrA*-MIR | Suppressor strain obtained on cephalotin 10µg/ml with mutation in inverted repeat of the *acrA* promoting region | This work |
| Δ*bla* P*acrA*-2IR | Suppressor strain obtained on cephalotin 10µg/ml with two mutations in inverted repeat of the *acrA* promoting region | This work |
| Δ*bla* P*acrA*-IIR | Suppressor strain obtained on cephalotin 10µg/ml with an insertion in the inverted repeat of the *acrA* promoting region | This work |
| Δ*bla* P*_acrA_*-IR^dw^ | Suppressor strain obtained on cephalotin 10µg/ml with mutation downstream of the inverted repeat of the *acrA* promoting region | This work |
| Δ*bla;tipR*-S53R, | Suppressor strain obtained on cephalotin 10µg/ml with mutation in *tipR* | This work |
| Δ*bla;tipR*-E119V | Suppressor strain obtained on cephalotin 10µg/ml with mutation in *tipR* | This work |
| Δ*bla;tipR*-S53R | Suppressor strain obtained on cephalotin 10µg/ml with mutation in *tipR* | This work |
| Δ*socB* | Deletion of the toxin *socB* | Sanselicio et al. 2015 |
| Δ*socB*;*clpP*::Ω | Insertion of an Omega cassette (Spc^R^) inside the *clpP* gene in a strain lacking the toxin socB | Sanselicio et al. 2015 |
| Δ*socB*;*clpX*::Ω | Insertion of an Omega cassette (Spc^R^) inside the *clpX* gene in a strain lacking the toxin socB | Sanselicio et al. 2015 |
| Δ*clpA* | Deletion of *clpA* | Jonas et al. 2013 |
| P*_xylX_*::P*_xyl_clpP** | Insertion of the pMO88 inside NA1000 | Sanselicio et al. 2015 |
| Δ*djlA* Δ*acrAB*-*nodT* | Deletion of *djlA* in Δ*acrAB-nodT* strain | This work |
| Δ*djlA*::pNPTS | Complete deletion of *djlA*, deleted in the Δ*acrAB-nodT* strain, re-tagged using the same pNPTS used for the deletion and then transduced in NA1000 | This work |
| Δ*ftsH* | Full deletion of *ftsH* | Jonas et al. 2013 |
| Δ*lon* | Full deletion of *lon* | Jonas et al. 2013 |
| Δ*bla;* P*_xylX_*::P*_xyl_djlA-HA* | HA tagged DjlA under the control of the xylose inducible promoter (UG14074) | This work |
| P*_xylX_*::P*_xyl_djlA-HA* | HA tagged DjlA under the control of the xylose inducible promoter (UG22103) | This work |
| P*_xylX_*::P*_xyl_dnaJ1-HA* | HA tagged DnaJ1 under the control of the xylose inducible promoter (UG22105) | This work |
| P*_xylX_*::P*_xyl_dnaJ2-HA* | HA tagged DnaJ2 under the control of the xylose inducible promoter (UG22109) | This work |
| P*_xylX_*::P*_xyl_dnaJ3-HA* | HA tagged DnaJ3 under the control of the xylose inducible promoter (UG22107) | This work |
| P*_xylX_*::P*_xyl_dnaJ4-HA* | HA tagged DnaJ4 under the control of the xylose inducible promoter (UG22111) | This work |
| P*_xylX_*::P*_xyl_dnaJ5-HA* | HA tagged DnaJ5 under the control of the xylose inducible promoter (UG22113) | This work |
| P*_xylX_*::P*_xyl_clpX-HA* | HA tagged ClpX under the control of the xylose inducible promoter (UG22115) | This work |
| Δ*bla* *tipR::Tn P_xyl_::djlA-HA* | HA tagged DjlA under the control of the xylose inducible promoter (UG22388) in a *tipR* transposon mutant | This work |
| *Escherichia coli* | | |
| EC100D | *F- mcrA Δ(mrr-hsdRMS-mcrBC) Φ80dlacZΔM15 ΔlacX74 recA1 endA1 araD139 Δ(ara, leu)7697 galU galK λ- rpsL (StrR) nupG* | Epicentre |
| BL21(DE3) | *F–ompT hsdSB (rB–, mB–) gal dcm (DE3)* | NEB |
| Plasmids | | |
| pLac290-P_acrA_ | P*_acrA_* cloned using PacrAShotFor/Rev to express *lacZ* | This work |
| pLac290-P_djlA_ | P*_djlA_* cloned using PromDjlAFor/Rev to express *lacZ* | This work |
| pLac290-P_ccrM_ | P*_ccrM_* controlling the expression of *lacZ* |  |
| pSRK-*tipR* | The *tipR* cloned in pSRK-Gm using TipRndeI/sacI, inducible by IPTG | This work |
| pLac290-P_acrA_*nptII* | pLac290P_acrA_ with the *nptII* gene cloned in-frame at the beginning of *lacZ* | This work |
| pSRK-*acrAB-nodT* | Full *acrABnodT* operon under the control of IPTG | This work |
| pMT335-*djlA* | *djlA* clone in the Van inducible plasmid pMT335 using DjlAFor/Rev | This work |
| pLac290-P_bla_ | *lacZ* under the control of the promoter of the metallo-β-lactamase (CCNA_02223) | This work |
| pMT-*gyrA’* | pMT335 carrying *gyrA*F96 Nal sensitive | Kirkpatrick et al. 2014 |
| pMT-*gyrA(BM)* | pMT335 carrying *gyrA* from *Brucella melitensis* | Kirkpatrick et al. 2014 |
| pET21-*tipR* | The *tipR* cloned in pET21 using TipRndeI/sacI, under the control of the T7 promoter | This work |

| Primer name | Sequence | Used |
| --- | --- | --- |
| PacrAShotFor | GGCGGCGAGTCCCACGGTCGT | β-galactosidase or *nptII* fusion and EMSA probe |
| PacrAShotRev | GAAGTCCTGTGCGGCATTACATTT |  |
| TipRndeI | AAAACATATGCCGCACAGGACTTCCATAA | To clone *tipR* in pSRK for expression |
| TipRsacI | AAAAGAGCTCTAGCTCAGCAGGTGCCGCACCGT |  |
| DjlAFor | AAAAACATATGTCCTTCTGGCGCAACAT | Protein expression |
| DjlARev | AAAAAGAATTCGATTGTCTCGCCTACCCT |  |
| PromDjlAFor | ATGTTGCGCCAGAAGGACAT | β-galactosidase fusion and EMSA probe |
| PromDjlARev | TAGGAAGAGGGCGTTCGCCAT |  |
| PromCcrMFor | TACCACGCGAAGGTCCAT | EMSA probe |
| PromCcrMRev | GCGTTCATCTGCTCGATG |  |
| MutTipRlow | ACGCCGAGGCGGTGTGGGACAA | Mut Seq |
| MutTipRup | CATGGGGAGCTTGCCTTAGC |  |
| MutacrB1 | AAGGTGCGTCCCGGCGCGCCGATCAA |  |
| MutacrB1b | AAGCTGCGGTTGAACCAGCCGAAGAA |  |
| MutacrB2a | AAGGCCGGTCACCACGAGGAGAA |  |
| MutacrB2 | AGGCGACCAGGATCAGGGTGAA |  |
| MutNodT | GCTGCGTAACTTCACCCTGA |  |
| MutNodTb | GGAGGCCTATCGCAGCTT |  |

| Synthetic gene | Strain | Sequence |
| --- | --- | --- |

P*_xylX_*::P*_xyl_djlA-HA* in pUCIDT (Kan) *(strain* UG14074) :

AGATCTGCTAGCccagccacaggcccgtgccgggatcgaaggcgaagcggcagccgatcaggcggaactgggcgcgggccatggtctcgaacagggccgtcaggtcgcgggcggcgtccaggtcgtcgtggtccagcaccaggcagcggatctgcagccagccgtggtcgggcagcaggtagaaggcgccctcgtcctgatcctcgcccgaaacctccagcccccggtcgatggcttcgacgacatagccggccgcgcggcaggtgtcggtgagcgcggccagcagggcggcttcctggtcaggggtcaggtcggtcatgggcaagaggtccaggtcgtggtttgtcggcggcttctagcatggaccgcccgcgcccgtgaggccgaggatttcgcgctggtcagacaacctacttgccgtccccacatgttagcgctaccaagtgccgacgaacgcgcgccgccgacggtgtcggcgcttcagacgctcgagttttggggagacgacgccatATGTCATTCTGGCGCAATATTGCAAGCATTGCCGCTCGCCGTTTAGATTTGGCGGACTGTACTGAGTGTCCAGGAGGTTTGCCGGGAGAGGAcCCCGCATTTTCGACCGCTGTAACGGCACTTGGAGCTAAACTTGCGAAGGTGGACGGGCGCGCAGACGGTGGTGAGTTCGCGGCCTTCACAGAGGTGTTCCAACCAGATCCGGCCTCAGAGCCCAATATTCACCGTCTTTATGACTTGGCTCGCCAAACTACTCATGGGTTTGAGTCATAcGCAAAGCGCTTGGCGAAGCGCTATTCATCTTGTCCCCAGTTATTAGAGGACGTTGTTGACGGTTTATTCCATATTGCGAAATCCGATGGAATGGTGTCTCAGGACGAATTAGACTACCTGGAACGCGTGTCCAACTTGTTTGGTATGAGTCCTCTTGCCTTCCGTCGCCTTCGCGCGACACATTTAGGGGTAGACGCCAACGATCCTTATGCCATCTTAGAAGTCCCTCCGGACGCTGACGACGCTACTGTACGCTCGGCTTGGAAAGCAGCCCTGAGTTCCGCTCATCCTGATCGCGCTCGCGCCCGTGGTTTACCAACGGAgTTCATCGAAGTAGCCGAAGCGAAGGCTGCTTCTATTAACGCTGCTTTCAGTACCGTGATGCGCGAGCGTCGTGAGCTTGGATTAGCTGCCGCCGGTtacccatacgacgtcccagactacgctTGAattctagactcgagagctcgcggccgca

P*_xylX_*::P*_xyl_djlA-HA* in pUCIDT (Kan) *(strain* UG22103) :

caattgactagtcccagccacaggcccgtgccgggatcgaaggcgaagcggcagccgatcaggcggaactgggcgcgggccatggtctcgaacagggccgtcaggtcgcgggcggcgtccaggtcgtcgtggtccagcaccaggcagcggatctgcagccagccgtggtcgggcagcaggtagaaggcgccctcgtcctgatcctcgcccgaaacctccagcccccggtcgatggcttcgacgacatagccggccgcgcggcaggtgtcggtgagcgcggccagcagggcggcttcctggtcaggggtcaggtcggtcatgggcaagaggtccaggtcgtggtttgtcggcggcttctagcatggaccgcccgcgcccgtgaggccgaggatttcgcgctggtcagacaacctacttgccgtccccacatgttagcgctaccaagtgccgacgaacgcgcgccgccgacggtgtcggcgcttcagacgctcgagttttggggagacgacgccatATGTCTTTTTGGCGCAACATCGCCAGTATCGCCGCTCGTCGCTTAGACCTGGCGGACTGCACAGAGTGTCCTGGAGGGCTTCCTGGCGAAGATCCTGCTTTTTCGACCGCCGTGACCGCACTGGGGGCGAAACTGGCTAAGGTAGACGGTCGCGCTGACGGAGGCGAATTTGCAGCCTTTACCGAAGTGTTTCAGCCCGACCCTGCTTCGGAACCCAACATTCATCGTTTGTATGATTTGGCCCGCCAAACAACCCACGGATTTGAAAGCTACGCTAAGCGTTTAGCAAAGCGTTATTCAAGCTGCCCCCAGTTACTGGAGGACGTTGTTGACGGCTTATTCCACATCGCAAAATCGGACGGCATGGTGTCTCAGGATGAATTAGACTACCTGGAGCGCGTAAGTAACTTATTCGGAATGTCCCCCCTTGCTTTCCGTCGCCTGCGCGCAACACACTTGGGAGTGGACGCGAACGATCCCTATGCCATTTTGGAAGTACCGCCAGATGCAGACGATGCAACGGTCCGCTCTGCCTGGAAGGCTGCTCTGTCCTCCGCCCACCCTGACCGTGCGCGCGCACGTGGGTTGCCAACGGAGTTCATCGAGGTGGCAGAAGCTAAAGCCGCTTCTATCAATGCTGCCTTCTCGACCGTGATGCGTGAGCGTCGCGAATTAGGTTTGGCTGCCGCGGGAgcatatccttacgatgtacccgactacgcatatccgtatgacgtccctgactatgcaTGAATTCTAGAAGCGGCCAAGCTTGAAGCTAGCTACGTAGGCCTGCGGCCGCG

P*_xylX_*::P*_xyl_dnaJ1-HA* in pUCIDT (Kan) *(strain* UG22105) :

caattgactagtcccagccacaggcccgtgccgggatcgaaggcgaagcggcagccgatcaggcggaactgggcgcgggccatggtctcgaacagggccgtcaggtcgcgggcggcgtccaggtcgtcgtggtccagcaccaggcagcggatctgcagccagccgtggtcgggcagcaggtagaaggcgccctcgtcctgatcctcgcccgaaacctccagcccccggtcgatggcttcgacgacatagccggccgcgcggcaggtgtcggtgagcgcggccagcagggcggcttcctggtcaggggtcaggtcggtcatgggcaagaggtccaggtcgtggtttgtcggcggcttctagcatggaccgcccgcgcccgtgaggccgaggatttcgcgctggtcagacaacctacttgccgtccccacatgttagcgctaccaagtgccgacgaacgcgcgccgccgacggtgtcggcgcttcagacgctcgagttttggggagacgacgccatATGCGTGATTACTACGAAATTTTAGGAGTCACTCGCACCATCGATGAAGCGGGACTGAAATCAGCGTTTCGTAAACTGGCGATGGAGCATCACCCCGATCGTAATGGGGGGTGCGAAAACGCGGCGGGACGCTTCAAAGAGATTAACGAAGCATATTCCGTATTGTCTGATCCTCAGAAGCGCGCAGCCTACGACCGTTTCGGTCACGCCGGAGTAAATGGACCTCAGGGAGGGCCGGGTGGTTTTGGAGGGCAGGGATTCGACGCGAGCGATATTTTTAACGATGTTTTCGGGGATGTATTTGGTGAAATGTTTGGGGGAGGTCGTCGCCAGTCAAACGCGCCTCAGCGCGGTCAGGATTTGCGCTATGACCTTGAAATCACGTTAGAGCAAGCATACGCCGGGGCGGAGGTAGAGATCACCGTCCCCGCAGCTATGACGTGTGAGGTGTGCGAAGGGAGCGGCGCCAAACCGGGGACCAGCCCGTCCGTATGTGGAACTTGTGGTGGTGCCGGCCGTGTCCGCGCGACCCAAGGCTTCTTCGCTGTTGAACGCGGCTGTCCACGCTGCGGGGGAAGCGGACGTCTTGTACTGGACCCGTGCTCAAATTGTCACGGCCACGGTCAAGTGCGCCGTGAACGTATTCTGTCTGTCCGCATCCCCGCTGGAGTAGATGACGGCGCCCGTATTCGCCTGGCTGGGGAGGGTGATGCAGGAGCCCGTGGTGGGCCGCGCGGTGACCTGTATATCTTTTTAAGTGTGACCCCTCACGAGTTATTTGAACGCGATGGACTTGATCTTCTGTGCACAGTCCCGGTACCGATGACTACCGCAGCTCTTGGCGGCGAGATTGATGCTCCATGTCTTCTGGGAGGGGAATCATGCGATGGGGAATGCAAAGTCAAAGTCCACGTACCGGAAGGAGCGCAAACGGGCAAGACGGTGCGTTTAAAGGGTAAAGGAATGCCTAGCCTGCGTTCTCGTCAACGTGGAGACTTGGTAGTTGAGTTATTTGTCGAAACTCCGACGCATCTTTCAGCGCGCCAGAAAGAGCTTATGCGCGAGTTAGCGGGATTATGCGGTGAGAAGCAGAACCCTAAGTCCGCTAACTTCGTGGGTAAAGCCAAGCGTTTTTGGGAGGAGGTCACCGGATCTgcatatccttacgatgtacccgactacgcatatccgtatgacgtccctgactatgcaTGAATTCTAGAAGCGGCCAAGCTTGAAGCTAGCTACGTAGGCCTGCGGCCGCG

P*_xylX_*::P*_xyl_dnaJ2-HA* in pUCIDT (Kan) *(strain* UG22109) :

caattgactagtcccagccacaggcccgtgccgggatcgaaggcgaagcggcagccgatcaggcggaactgggcgcgggccatggtctcgaacagggccgtcaggtcgcgggcggcgtccaggtcgtcgtggtccagcaccaggcagcggatctgcagccagccgtggtcgggcagcaggtagaaggcgccctcgtcctgatcctcgcccgaaacctccagcccccggtcgatggcttcgacgacatagccggccgcgcggcaggtgtcggtgagcgcggccagcagggcggcttcctggtcaggggtcaggtcggtcatgggcaagaggtccaggtcgtggtttgtcggcggcttctagcatggaccgcccgcgcccgtgaggccgaggatttcgcgctggtcagacaacctacttgccgtccccacatgttagcgctaccaagtgccgacgaacgcgcgccgccgacggtgtcggcgcttcagacgctcgagttttggggagacgacgccatATGACCGCCCGTCAGTCCGCGTTAACTCTGTCTGCAGCTCGTGCATTGTTAGGTGTGGCTGCTGACGCCGATGAACGCGAACTTCGTAAGGCATACCGTGAAGCAGCCAAGCGCGCCCACCCTGACCGTCCCACGGGAGACGCAGCCTTGTTTCGTGATGTTCTTGCCGCTTACCGCCTGTTGCAGGATACTCCTGTGGTACGTCATCATTTTCCCCCGGCCGTTACCCCGCCTCCGATCGCCGACCGTGTGTTCCTTGAAATTGATATTGCCACTGCTCTGTCCGGTGGTGCGGAGGAATTGGCTATTGATGGACGCCGCCTTCGCTTGAAATTGCCCGCTGGGCTGCGTGAAGGCGATAAGGTTCGCGTAGAGGGAGTATGCTTCGAGGTACGTCTTCGTGCTCAGGATGGTGCAATGGTTCGCGGTGACGACCTTTGGCTGACGGGGAAGGTTGATCCGCGCGTGTTAGCCGAGGGGGGTCGCGTTGATGCTGATACACCCCTTGGTCCCCGCCCTGCTTGGATCAGCACTAAAGCTGCTGCTCGTGGACTGGTTCGTTTGCCCGGACAGGGGCTTCCAGCTCGCGCTAACCACAAGGCGGGCGATCTTTTTTTGCGTCTGGAAGCCTCGGAGGGTCGTGGCGAGAGTCCCGCTCGCAGTCTGCTTAAACGCTTCGCCGCAGCGTGGGCTGCAgcatatccttacgatgtacccgactacgcatatccgtatgacgtccctgactatgcaTGAATTCTAGAAGCGGCCAAGCTTGAAGCTAGCTACGTAGGCCTGCGGCCGCG

P*_xylX_*::P*_xyl_dnaJ3-HA* in pUCIDT (Kan) *(strain* UG22107) :

caattgactagtcccagccacaggcccgtgccgggatcgaaggcgaagcggcagccgatcaggcggaactgggcgcgggccatggtctcgaacagggccgtcaggtcgcgggcggcgtccaggtcgtcgtggtccagcaccaggcagcggatctgcagccagccgtggtcgggcagcaggtagaaggcgccctcgtcctgatcctcgcccgaaacctccagcccccggtcgatggcttcgacgacatagccggccgcgcggcaggtgtcggtgagcgcggccagcagggcggcttcctggtcaggggtcaggtcggtcatgggcaagaggtccaggtcgtggtttgtcggcggcttctagcatggaccgcccgcgcccgtgaggccgaggatttcgcgctggtcagacaacctacttgccgtccccacatgttagcgctaccaagtgccgacgaacgcgcgccgccgacggtgtcggcgcttcagacgctcgagttttggggagacgacgccatATGATCTACTTACTTCTTGGAGCAGTGATCATCACTTTCCTTTTGTGGCCCCGCGGCCGTGCTCTTCTTAAGGGTGATGGATGGCGTGTCGGAGCTGGGGCTGCTGCTATCGCCGCATTTGCAGTGGCGGCCTACGCAACCATTCGCGGTGCCTGGGGTACGGGTATTGTATTGGGCGTCATTGGCCTTTGGTCCGTAACTGAGGCGCGTCGTCGTCCAATCGTACGTCGTGAGGTGGTACATCCTCCGAAACCTGAGTTAAGTTTGTCAGAGGCTCGCGCTATTTTGGGTGTCGGTCCAGAGGCGTCGTTAGCAGAGGTGAAAGCCGCATACAACCGTCTGATCCAAATGGCACATCCCGATAAAGGTGGAACCGAGGGTTTGGCTGCCCAGCTGAACGCCGCGCGTGATCGTCTTATCAAGCCGCGCGGGTCTGCCCGCGTAGAACCGgcatatccttacgatgtacccgactacgcatatccgtatgacgtccctgactatgcaTGAATTCTAGAAGCGGCCAAGCTTGAAGCTAGCTACGTAGGCCTGCGGCCGCG

P*_xylX_*::P*_xyl_dnaJ4-HA* in pUCIDT (Kan) *(strain* UG22111) :

caattgactagtcccagccacaggcccgtgccgggatcgaaggcgaagcggcagccgatcaggcggaactgggcgcgggccatggtctcgaacagggccgtcaggtcgcgggcggcgtccaggtcgtcgtggtccagcaccaggcagcggatctgcagccagccgtggtcgggcagcaggtagaaggcgccctcgtcctgatcctcgcccgaaacctccagcccccggtcgatggcttcgacgacatagccggccgcgcggcaggtgtcggtgagcgcggccagcagggcggcttcctggtcaggggtcaggtcggtcatgggcaagaggtccaggtcgtggtttgtcggcggcttctagcatggaccgcccgcgcccgtgaggccgaggatttcgcgctggtcagacaacctacttgccgtccccacatgttagcgctaccaagtgccgacgaacgcgcgccgccgacggtgtcggcgcttcagacgctcgagttttggggagacgacgccatATGGCCCGTGATCCTTACCAGGAACTGGGTGTCACTCGCACCGCTTCAGCAGATGAGATTCGCAAGGCGTTTCGTAAGTTGGCGAAACAGTATCATCCAGATGCAAACCCTGGCGACAAAAAAGCTGAGGAACGTTTCAAACAGGTAAGTGCGGCTTTCGACATTGTCGGCGACGCAGAGAAACGCAAGAAGTTTGACCTTGGCCAAATCGATGCCGACGGGCGTGAGACAATGCGCGGCTTTGGTGGCCAACCGGGAAACGGGCCCTTTAATGCTGGAGGGTTCGGACAAGGGGGTTTCCACCGCAGTAATGAGGGGCCGGAAATCGACTTGTCAGATTTGTTTGGGGGTATGTTCGGAGGAGGCGGTCCTGGCGGCGCAGGGCGTGGTCCCTTTTCGGGAGGCGCGGGAGGCGGGTTTTCGGCTAAAGGTGCAGACGTAAAAGCTCGTTTAGACATTGACTTAGAGGACGCGATCAAGGGCGGTAAAAAGCGTGTCGCGTTCTCAGACGGCCGCACGATCGATGTCACCATCCCCACGGGCGCCCAGGAAGGGCAGACATTACGCCTGAAAGGTCAGGGTTCCCCCGGACGCGGAGGGCAAGGTGACGCTTTGATTGAATTGGCAATCAAGCCGCATCCTATTTACCGCCGCGAGGGTGAGGCATTAGTTATGGACCTTCCCGTCTCGATTCCAGATGCTGTATTGGGAGGCAAAGTCGAAGCCCCAACGCCTGATGGCAATGTAATGTTGGCAGTACCGAAAGGGAGCAACAGCGGGCAGACGCTTCGCTTGAAAGGACGCGGTATGCCGGATGGAAAAGGTAAACGCGGGGACTTGTTGGCGCGCTTAGTCGTCACTCTGCCAGAAACTGTTGATCAAGATCTTGAAAAATTCGCGGAgGCTTGGCGCGCGCAGAAGCCATACACACCCAAACGCAAAgcatatccttacgatgtacccgactacgcatatccgtatgacgtccctgactatgcaTGAATTCTAGAAGCGGCCAAGCTTGAAGCTAGCTACGTAGGCCTGCGGCCGCG

P*_xylX_*::P*_xyl_dnaJ5-HA* in pUCIDT (Kan) *(strain* UG22113) :

caattgactagtcccagccacaggcccgtgccgggatcgaaggcgaagcggcagccgatcaggcggaactgggcgcgggccatggtctcgaacagggccgtcaggtcgcgggcggcgtccaggtcgtcgtggtccagcaccaggcagcggatctgcagccagccgtggtcgggcagcaggtagaaggcgccctcgtcctgatcctcgcccgaaacctccagcccccggtcgatggcttcgacgacatagccggccgcgcggcaggtgtcggtgagcgcggccagcagggcggcttcctggtcaggggtcaggtcggtcatgggcaagaggtccaggtcgtggtttgtcggcggcttctagcatggaccgcccgcgcccgtgaggccgaggatttcgcgctggtcagacaacctacttgccgtccccacatgttagcgctaccaagtgccgacgaacgcgcgccgccgacggtgtcggcgcttcagacgctcgagttttggggagacgacgccatATGAATCGCCCCTTCGAATACCGTCCCAAATTCTATGACATCCGCGTCCGTCCCCCGAAGGAAGGTGAAGAGGATCCAGCACATGATGTATTGGGCCTTAAACCTGGGGAGAAACGCTGCGACCATCCAGACTGTCGCCTTGCAGGGAGCGCCAAAGCCCCGAAATCCCGCGATATGCCGGGAGATCATTACTGGTTCTGCCAACGCCATGCTGCTGAGTATAATAAGAATTGGAACTTCTACGCTGGAATGAGTGAAGCTCAAATCCGCGCTGAACAGGAGAGTGAGCGTATGACCGGGGGCCGTCCTACTTGGTCGTTCAAAGCAGATGCCAACTCGCGCGAAGCAGCAGCTATGGCGGCTCGTGATGCGCGCCATTTCGCTGACCCCTTTGGGGTATTTCGTGCGCAACAACGCCGCGCAGAAGCGGAGCGCAGTGCGGCTGAGCGTCGCCTGGGAAAACTTGAACGTCAGGCCCTTGCCGACCTTGACCTGGAAGCCACCGCAGATTCCGCTGCTATCCGCGCACGTTATAAAGAGCTGCTTAAACGTTGTCATCCGGACGCGAACGGCGGTGATCGCTCTGCTGAGCATAAGTTGCAGCGTGTTATTAAGGCGTATAAGCAATTACAAAAGAGCGGGATGGTCgcatatccttacgatgtacccgactacgcatatccgtatgacgtccctgactatgcaTGAATTCTAGAAGCGGCCAAGCTTGAAGCTAGCTACGTAGGCCTGCGGCCGCG

P*_xylX_*::P*_xyl_clpX-HA* in pUCIDT (Kan) *(strain* UG22115) :

caattgactagtcccagccacaggcccgtgccgggatcgaaggcgaagcggcagccgatcaggcggaactgggcgcgggccatggtctcgaacagggccgtcaggtcgcgggcggcgtccaggtcgtcgtggtccagcaccaggcagcggatctgcagccagccgtggtcgggcagcaggtagaaggcgccctcgtcctgatcctcgcccgaaacctccagcccccggtcgatggcttcgacgacatagccggccgcgcggcaggtgtcggtgagcgcggccagcagggcggcttcctggtcaggggtcaggtcggtcatgggcaagaggtccaggtcgtggtttgtcggcggcttctagcatggaccgcccgcgcccgtgaggccgaggatttcgcgctggtcagacaacctacttgccgtccccacatgttagcgctaccaagtgccgacgaacgcgcgccgccgacggtgtcggcgcttcagacgctcgagttttggggagacgacgccatATGACAAAGGCAGCGAGCGGTGATACAAAATCCACATTATACTGCTCATTTTGTGGGAAGAGTCAGCACGAAGTGCGCAAACTTATTGCCGGGCCCACGGTCTTCATTTGCGATGAGTGCGTAGAGTTATGCATGGACATCATTCGCGAAGAACATAAGATTGCATTCGTGAAAAGTAAGGACGGAGTGCCAACGCCGCGTGAGATCTGTGAAGTACTGGACGACTATGTGATCGGTCAGGGACACGCAAAAAAAGTACTGGCGGTAGCGGTTCACAATCATTATAAACGTCTTAATCACGCGAGCAAGAACAATGATGTAGAATTGGCAAAGAGTAACATTCTTCTGGTAGGGCCTACGGGGACGGGAAAAACTCTTCTGGCTCAAACGTTAGCGCGTATCATTGACGTACCGTTTACCATGGCTGACGCGACTACATTGACGGAAGCGGGTTACGTCGGTGAGGACGTTGAAAATATTGTGCTGAAACTGCTGCAAGCAGCCGATTATAACGTAGAGCGCGCCCAGCGTGGAATCGTCTATATCGATGAGATTGACAAAATCTCTCGTAAAAGTGATAACCCGAGCATCACGCGCGATGTGAGCGGTGAAGGGGTCCAACAGGCTCTTTTGAAGATTATGGAGGGTACTGTAGCTTCGGTGCCGCCACAAGGCGGACGTAAGCATCCTCAGCAGGAGTTCTTGCAGGTTGATACTACTAACATTTTGTTTATCTGCGGCGGGGCGTTCGCCGGATTGGAGAAGATTATCTCTGCTCGCGGGGCTGCCAAGTCGATTGGTTTCGGAGCTAAGGTGACCGATCCCGAAGAACGTCGTACAGGTGAGATCTTACGTAATGTAGAGCCCGACGATTTGCAGCGCTTCGGTCTTATTCCAGAATTTATCGGTCGCCTTCCGGTTGTGGCTACATTGGAAGACCTGGATGAAGCGGCTCTTGTCAAAATCTTGACCGAACCTAAGAACGCATTCGTTAAACAATATCAACGTTTATTTGAGATGGAAAATATCGGCCTTACGTTCACGGAAGATGCGCTTCATCAGGTGGCCAAAAAAGCGATCGCCCGCAAAACCGGAGCCCGCGGATTACGCTCCATCATGGAAGGCATCCTTTTGGAAACAATGTTTGAGTTACCTACGTACGAGGGTGTTGAGGAAGTGGTGGTGAATGCCGAGGTTGTCGAAGGCCGTGCACAACCTCTTCTGATTTATGCAGAAAAGAAAGGCGGCGCAGCGTCCGCCgcatatccttacgatgtacccgactacgcatatccgtatgacgtccctgactatgcaTGAATTCTAGAAGCGGCCAAGCTTGAAGCTAGCTACGTAGGCCTGCGGCCGCG
