# Supplementary material for: Adaptive β-lactam resistance from an inducible efflux pump that is post-translationally regulated by the DjlA co-chaperone
Source: PLoS Biol. 2023 Dec 5;21(12):e3002040. doi: 10.1371/journal.pbio.3002040 (PMC10754441; doi:10.1371/journal.pbio.3002040)
Supplement: S1 Raw images — (PDF) [file pbio.3002040.s031.pdf]

Figure 1

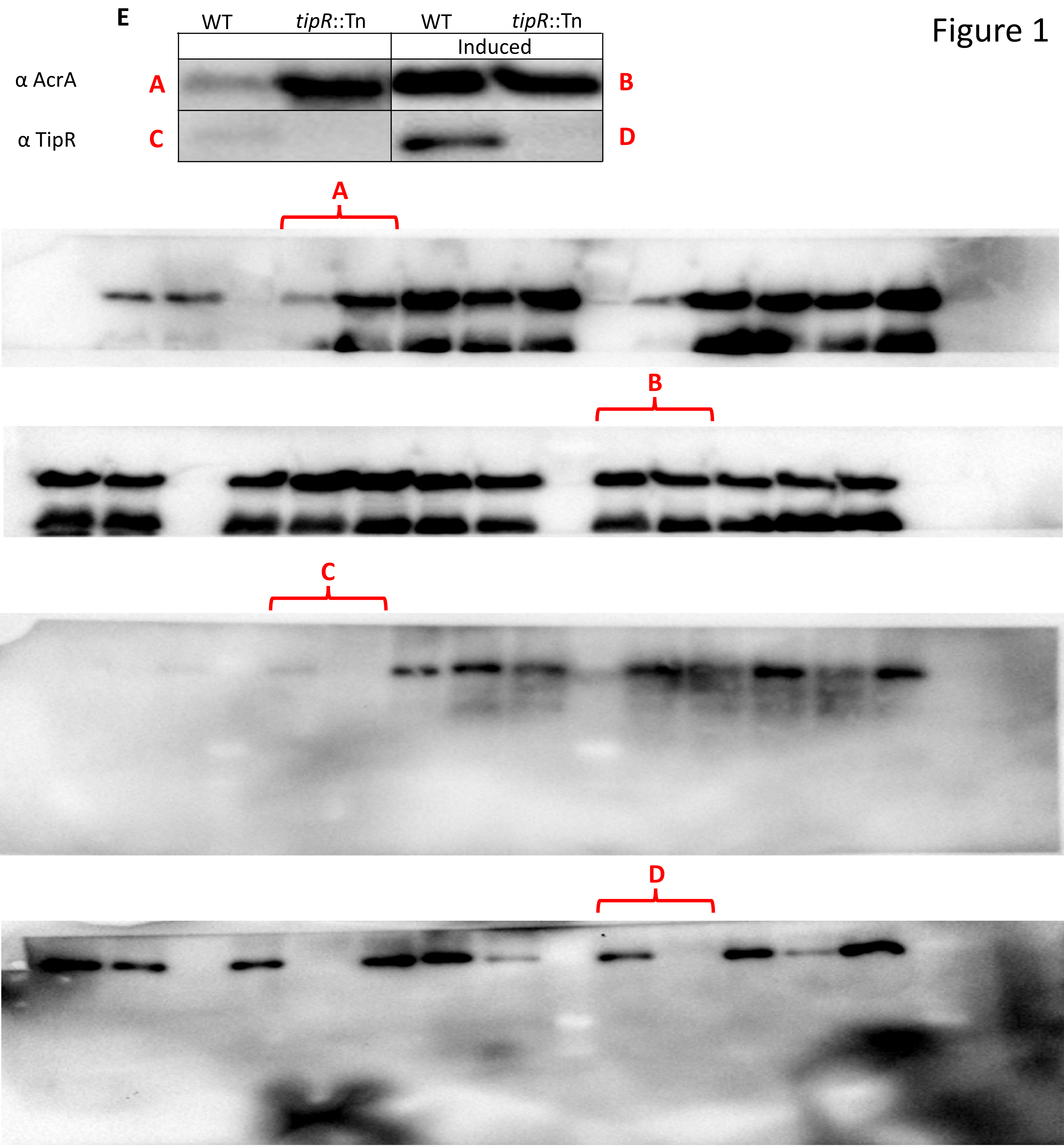

Figure 3

A

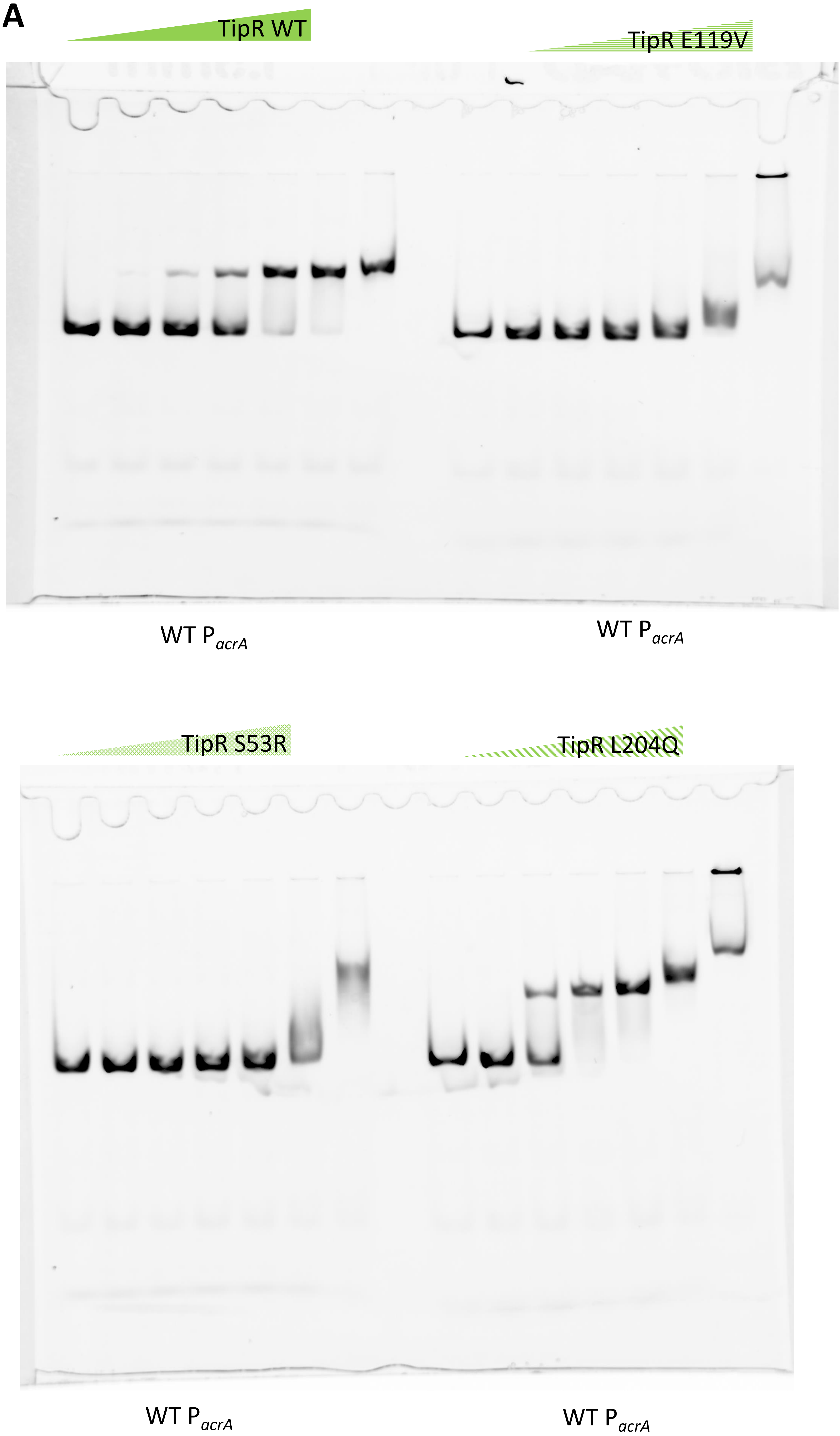

TipR WT
TipR E119V
WT P_{acrA}
WT P_{acrA}
TipR S53R
TipR L204Q
WT P_{acrA}
WT P_{acrA}

Figure 3

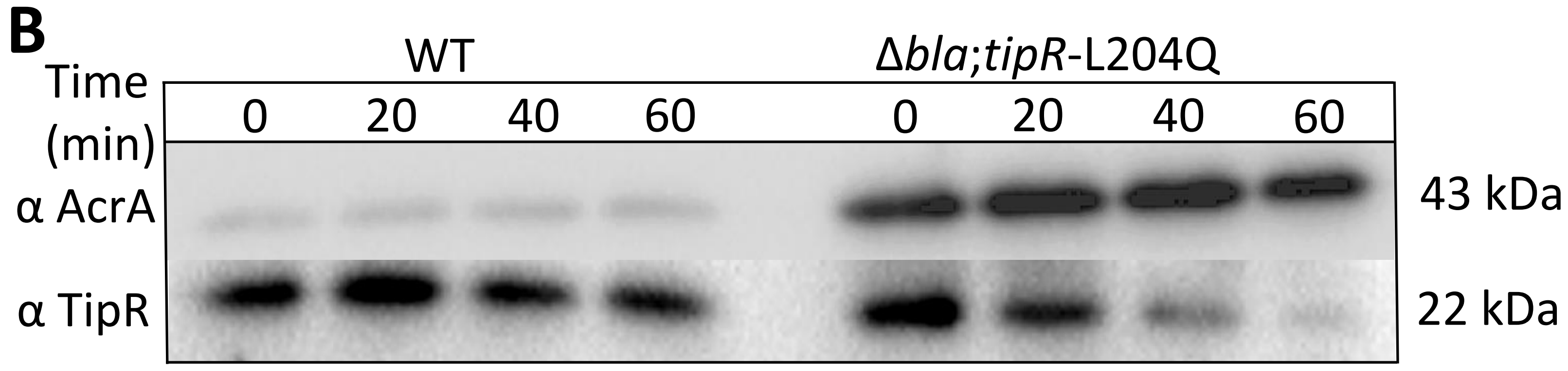
B
WT
Δ*bla*;*tipR*-L204Q
Time (min)
0
20
40
60
0
20
40
60
α AcrA
α TipR
43 kDa
22 kDa

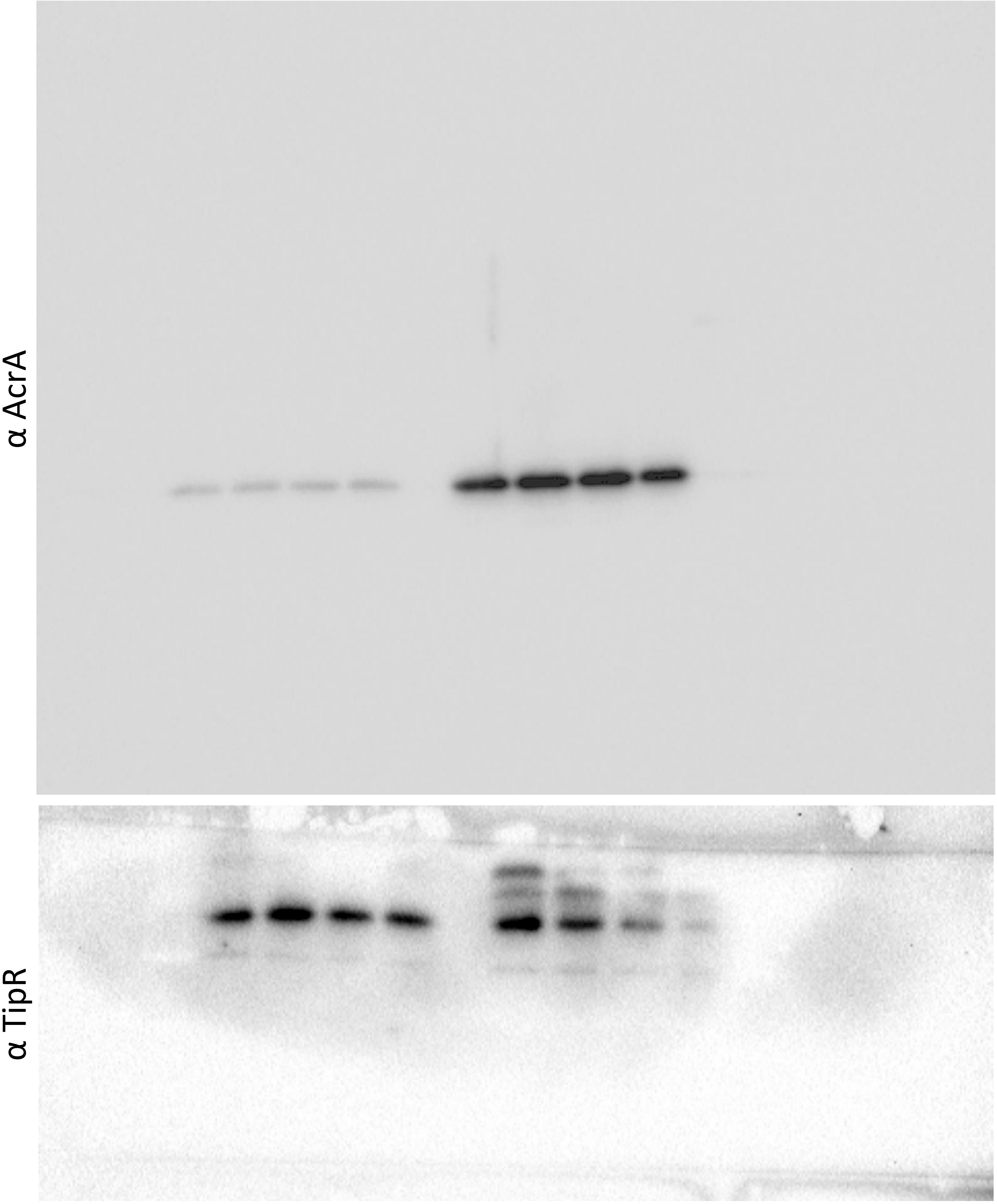
α AcrA
α TipR

Figure 3

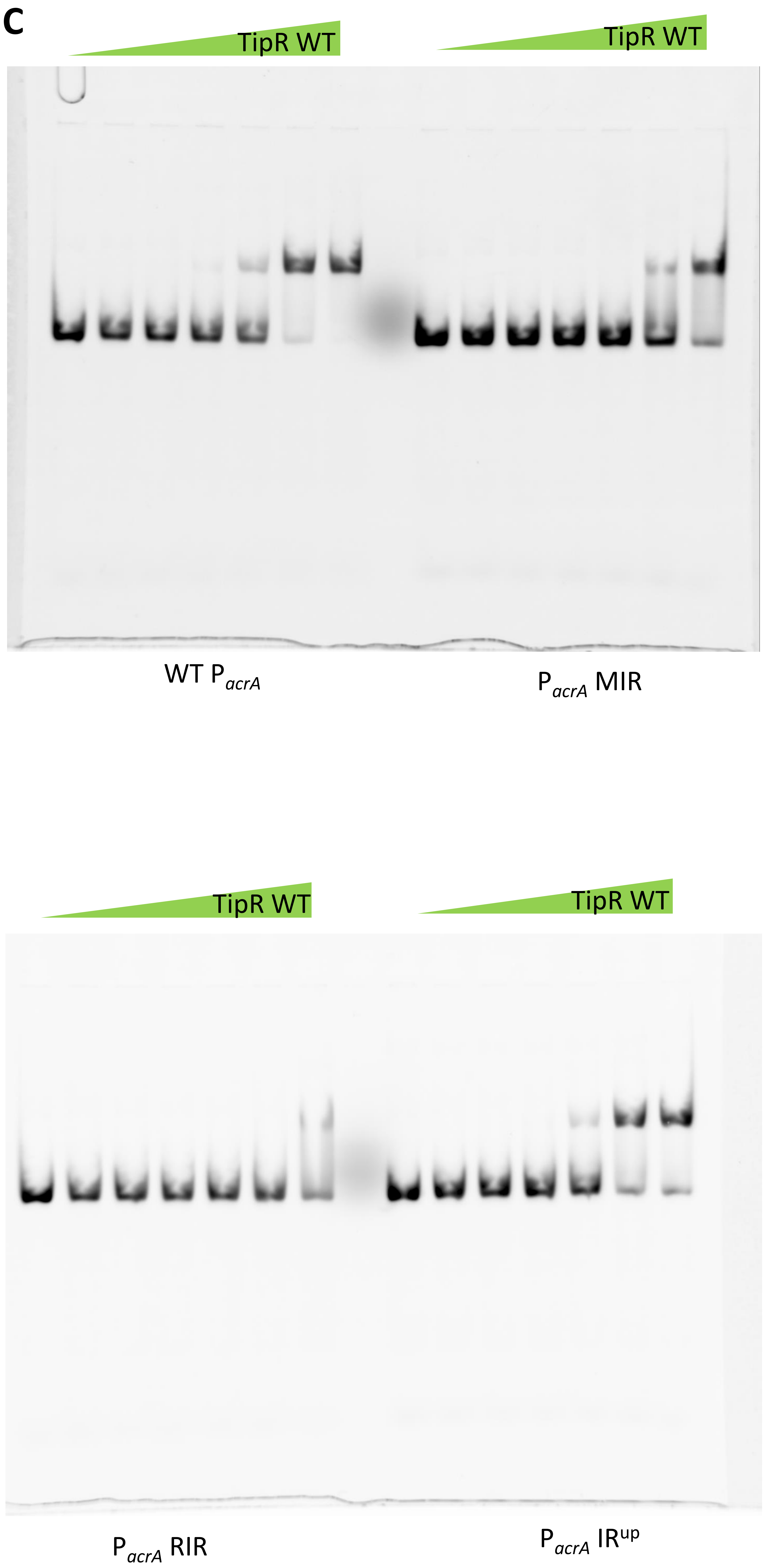
C
TipR WT
TipR WT
WT P_acrA
P_acrA MIR
TipR WT
TipR WT
P_acrA RIR
P_acrA IR^up

D

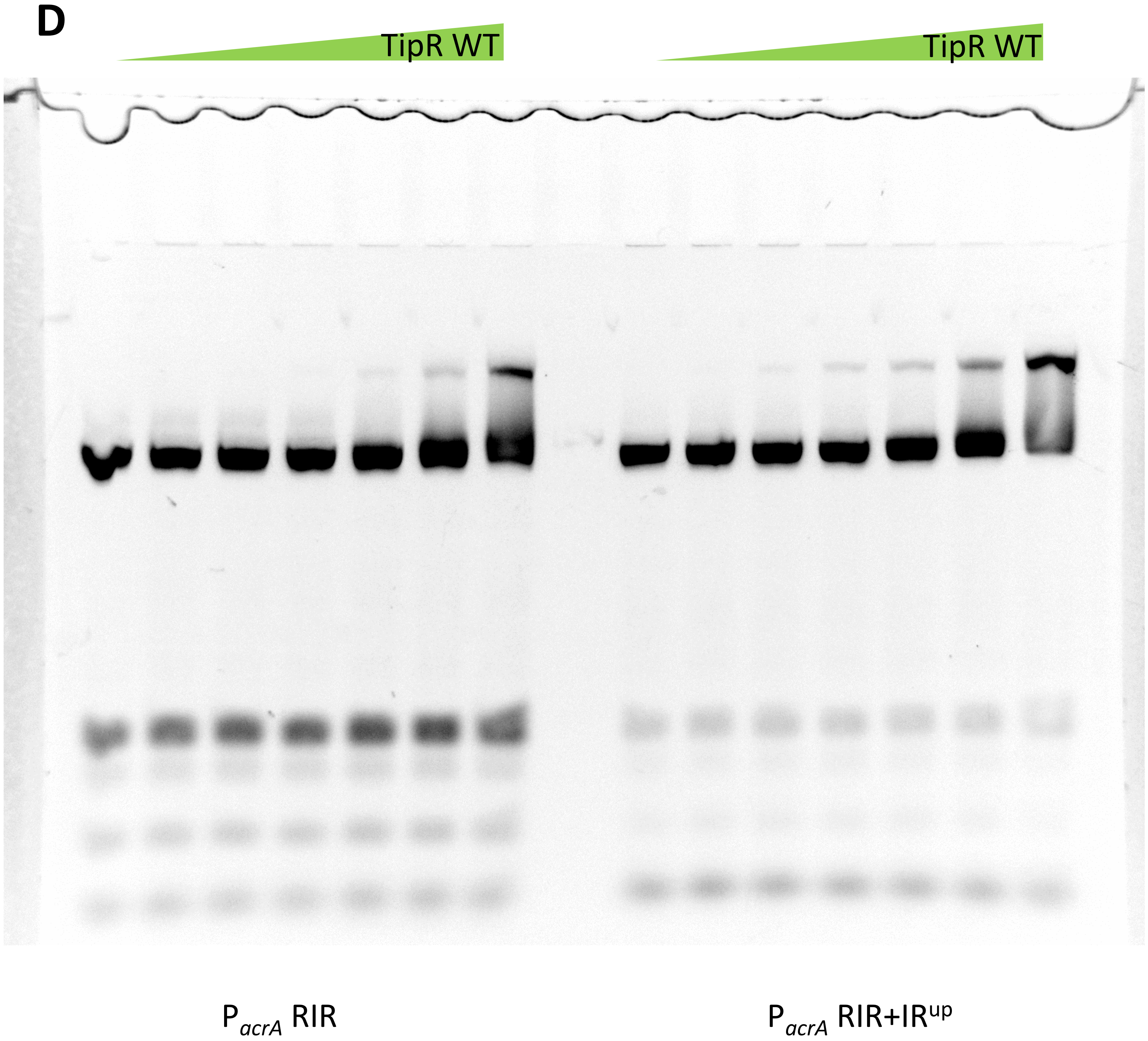

Figure 3

E

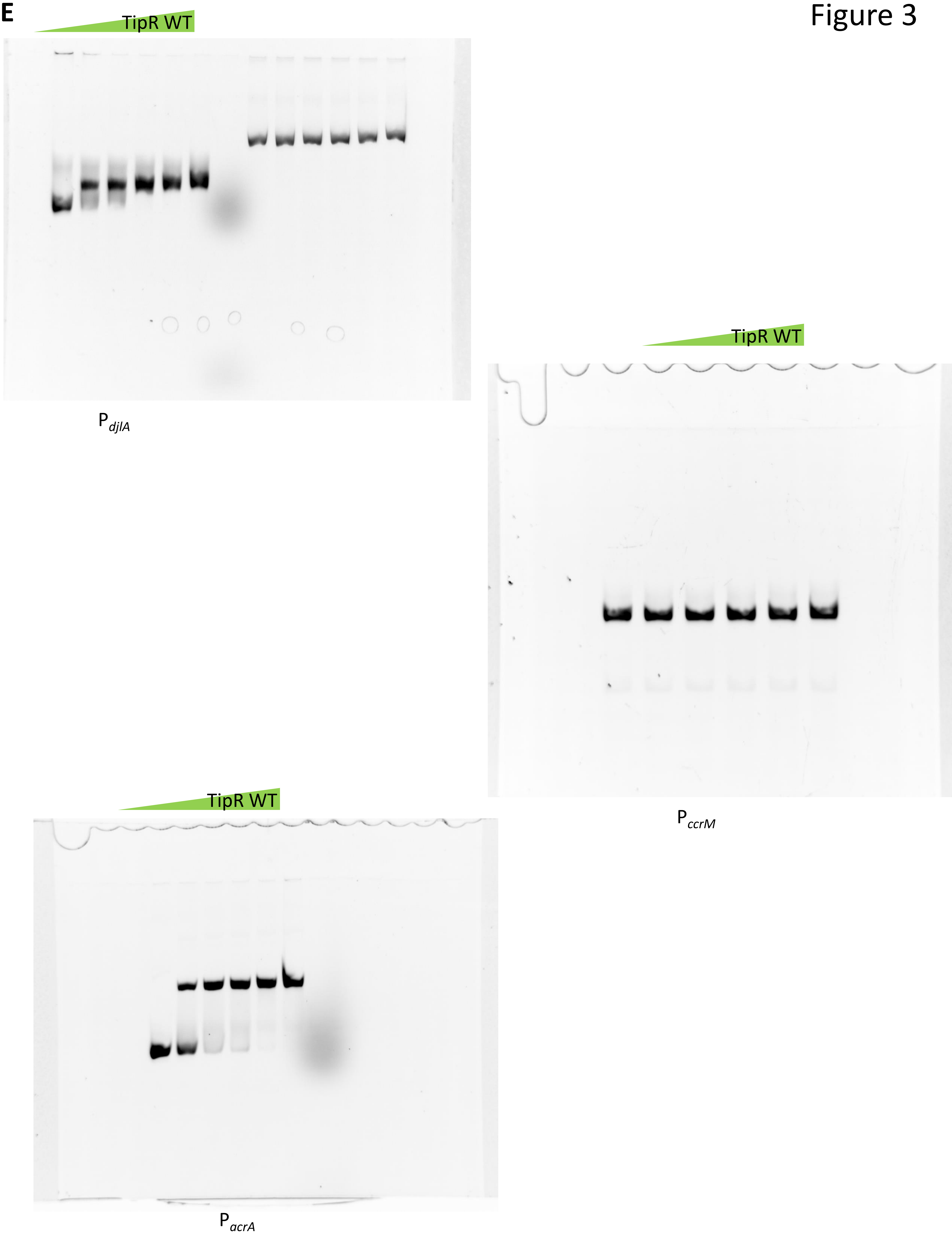

Figure 4

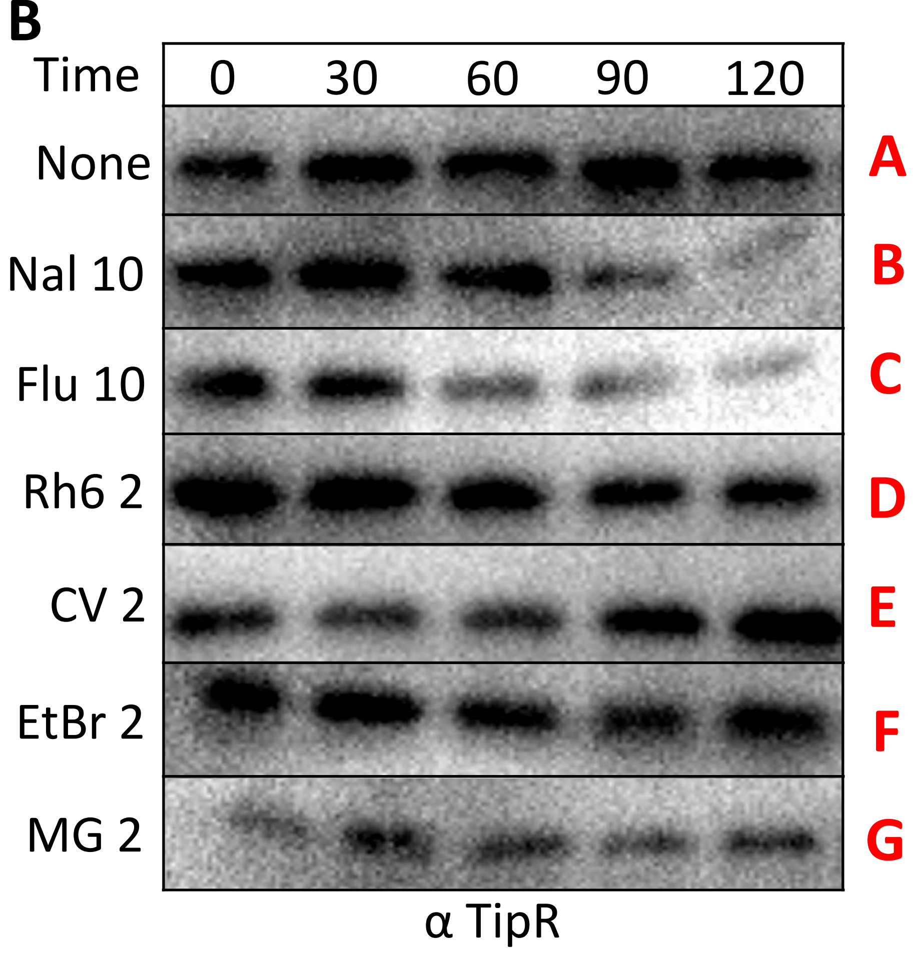

B
Time
0
30
60
90
120
None
Nal 10
Flu 10
Rh6 2
CV 2
EtBr 2
MG 2
A
B
C
D
E
F
G
α TipR

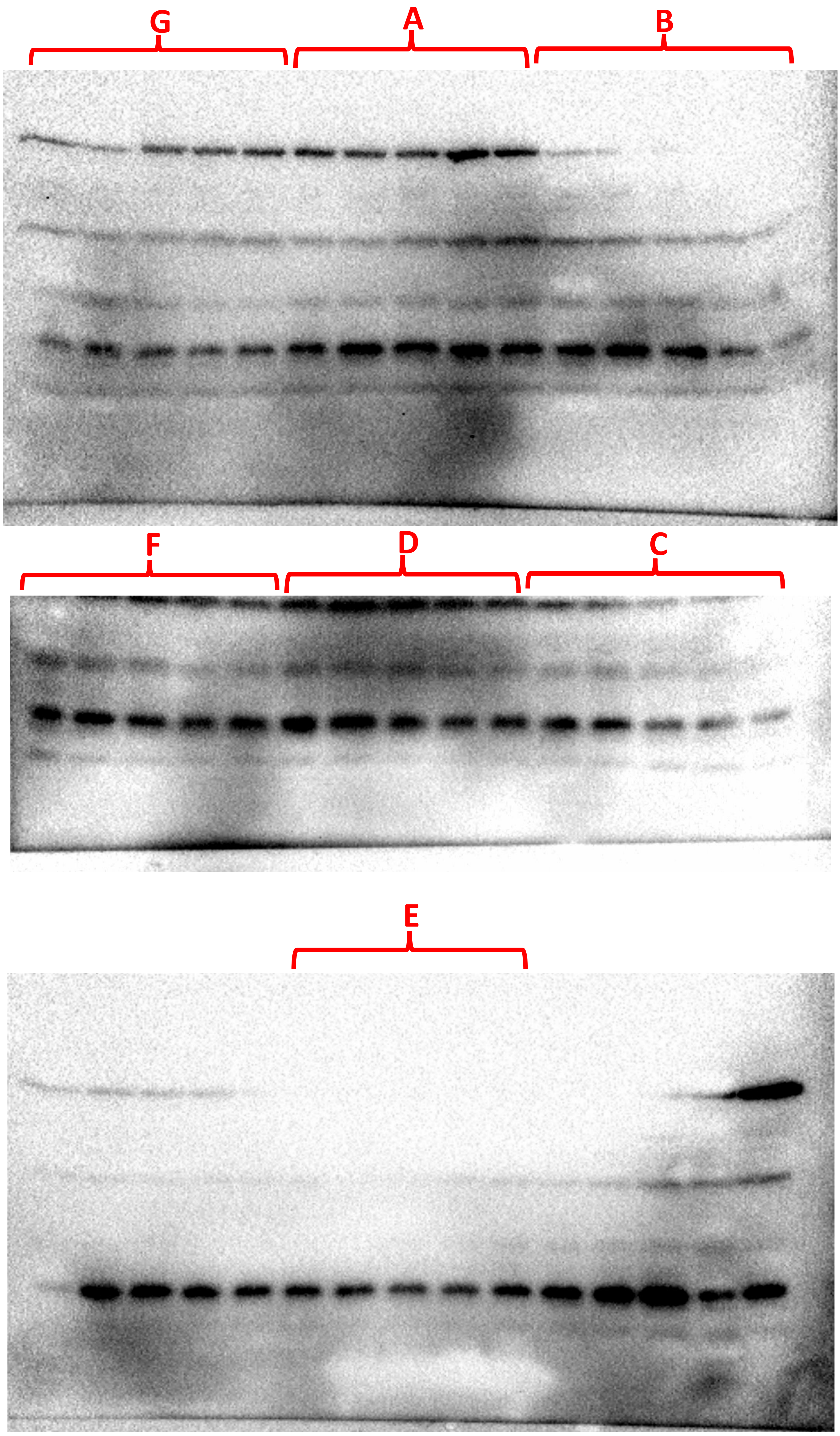

G
A
B
F
D
C
E

C

Figure 4

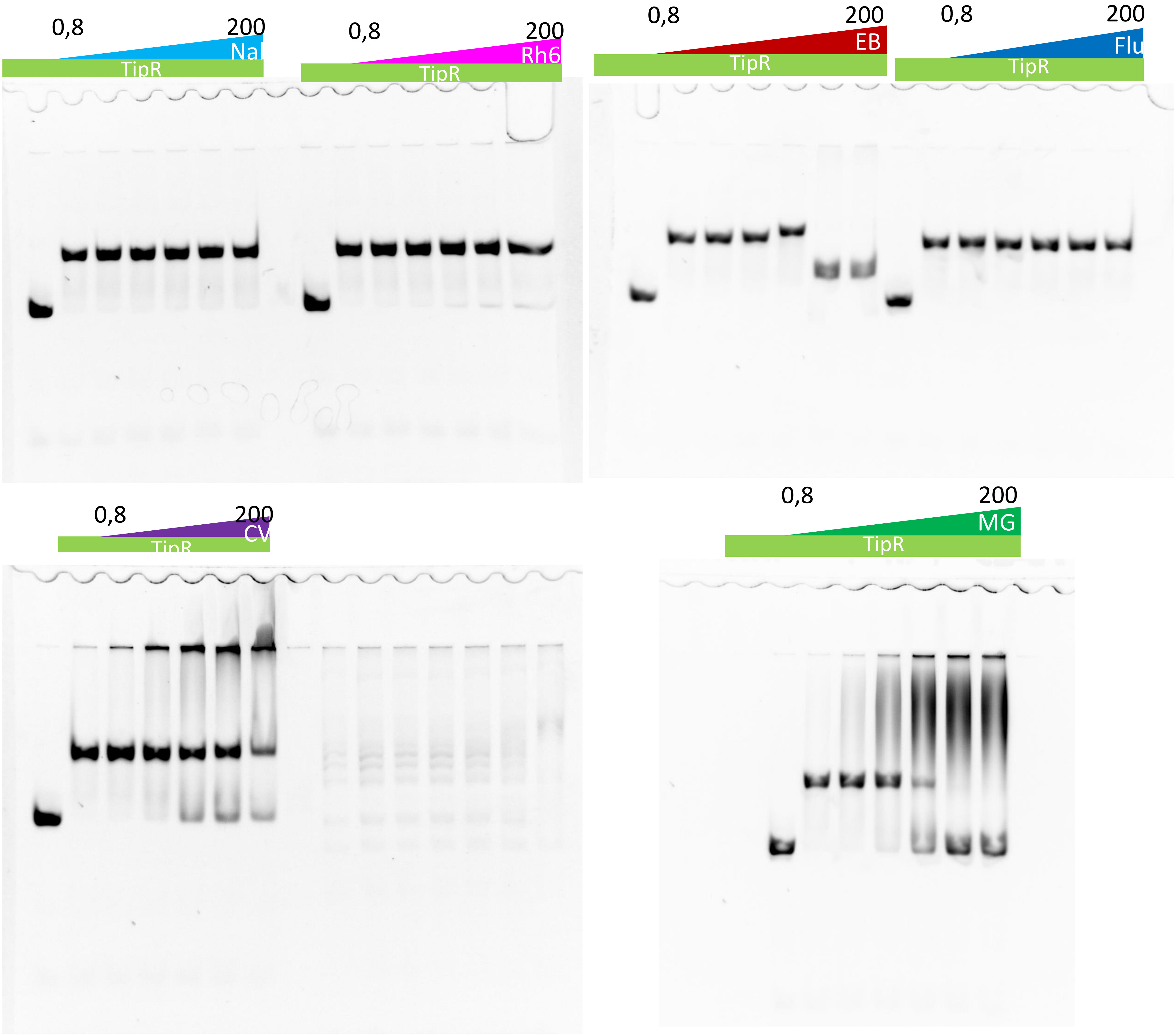
0,8
200
NaI
TipR
0,8
200
Rh6
TipR
0,8
200
EB
TipR
0,8
200
Flu
TipR
0,8
200
CV
TipR
0,8
200
MG
TipR

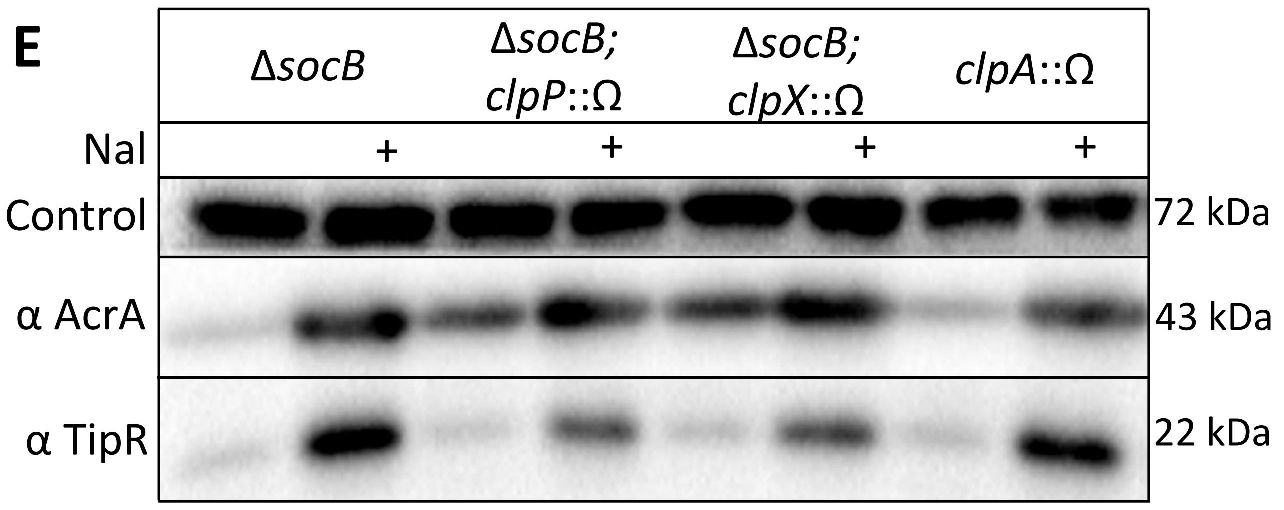
E
ΔsocB
ΔsocB; clpP::Ω
ΔsocB; clpX::Ω
clpA::Ω
Nal
+
+
+
+
Control
α AcrA
α TipR
72 kDa
43 kDa
22 kDa

Figure 4

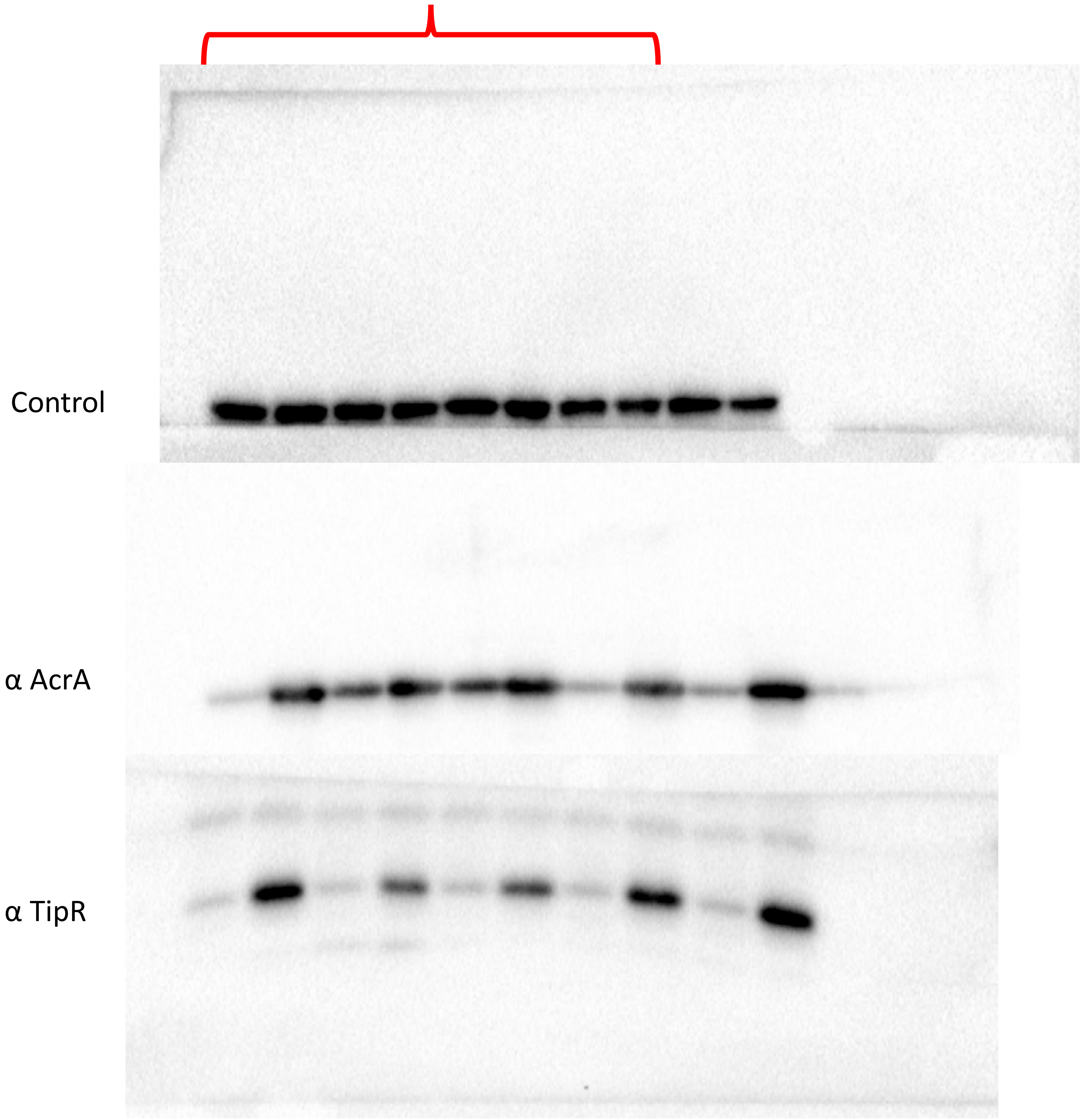
Control
α AcrA
α TipR

Figure 4

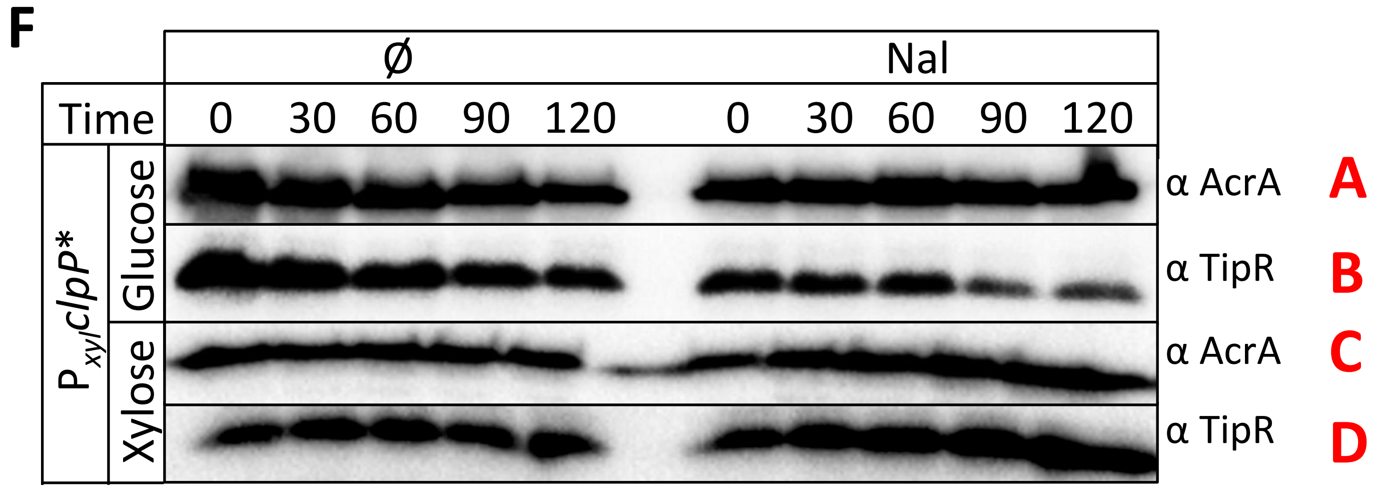
F
Ø
Nal
Time
0
30
60
90
120
0
30
60
90
120
$P_{xyl}clpP$*
Glucose
Xylose
α AcrA
A
α TipR
B
α AcrA
C
α TipR
D

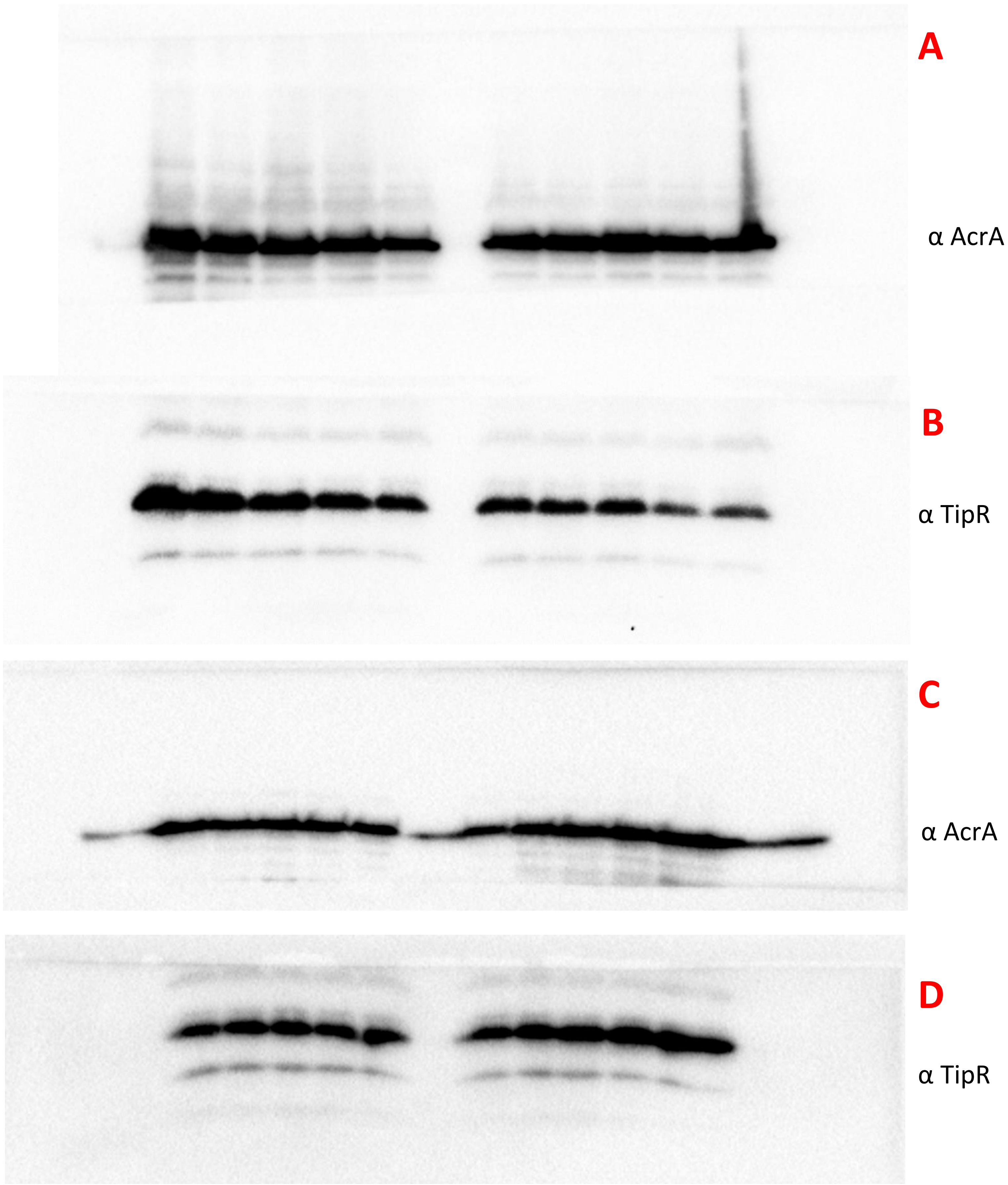
A
α AcrA
B
α TipR
C
α AcrA
D
α TipR

Figure 5

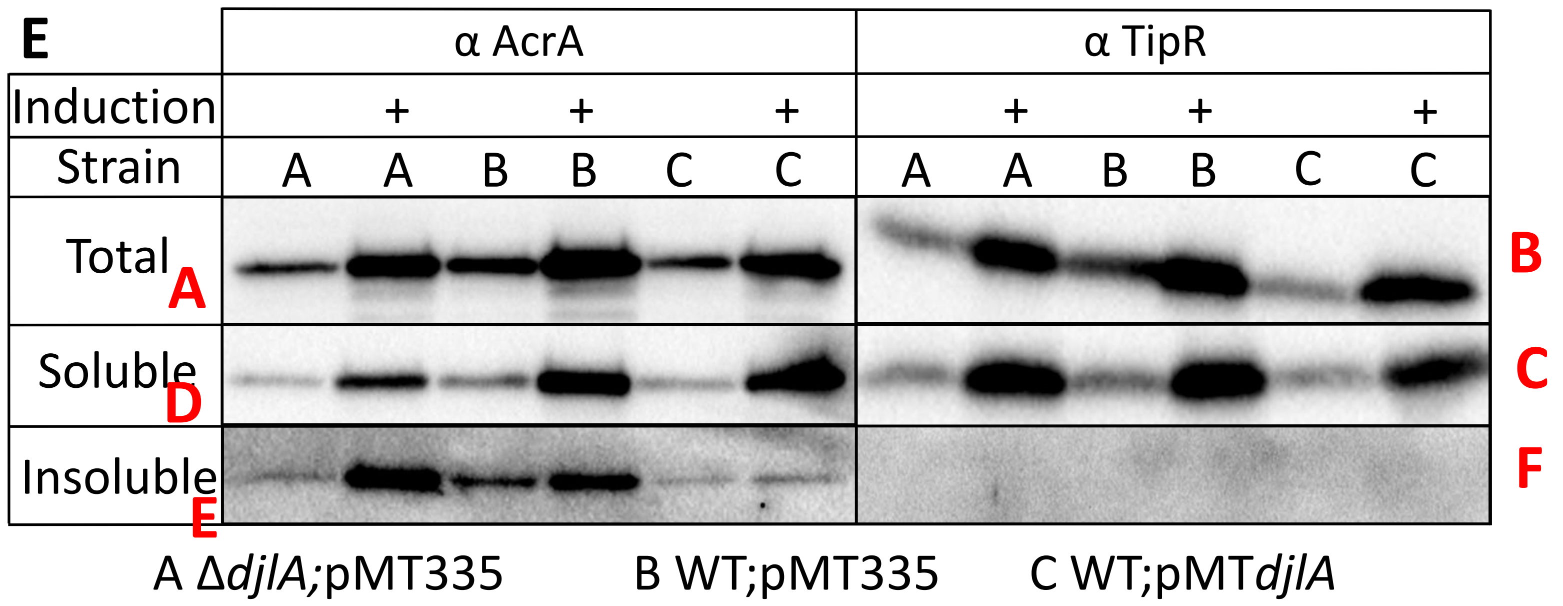
E
α AcrA
α TipR
Induction
+ + + + + +
Strain
A A B B C C A A B B C C
Total
Soluble
Insoluble
A
B
C
D
E
F
A Δ*djlA*;pMT335
B WT;pMT335
C WT;pMT*djlA*

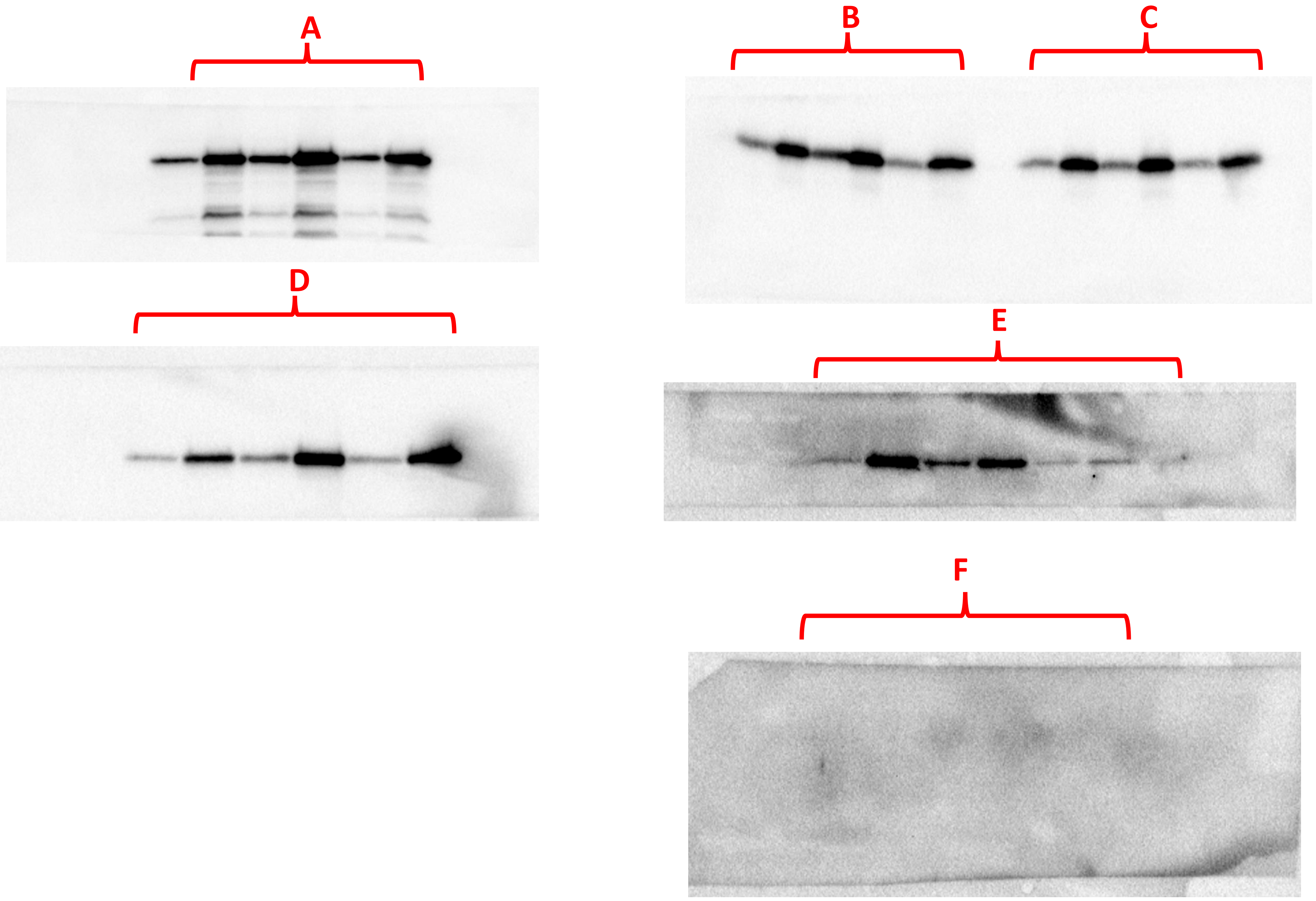
A
B
C
D
E
F

Figure S3

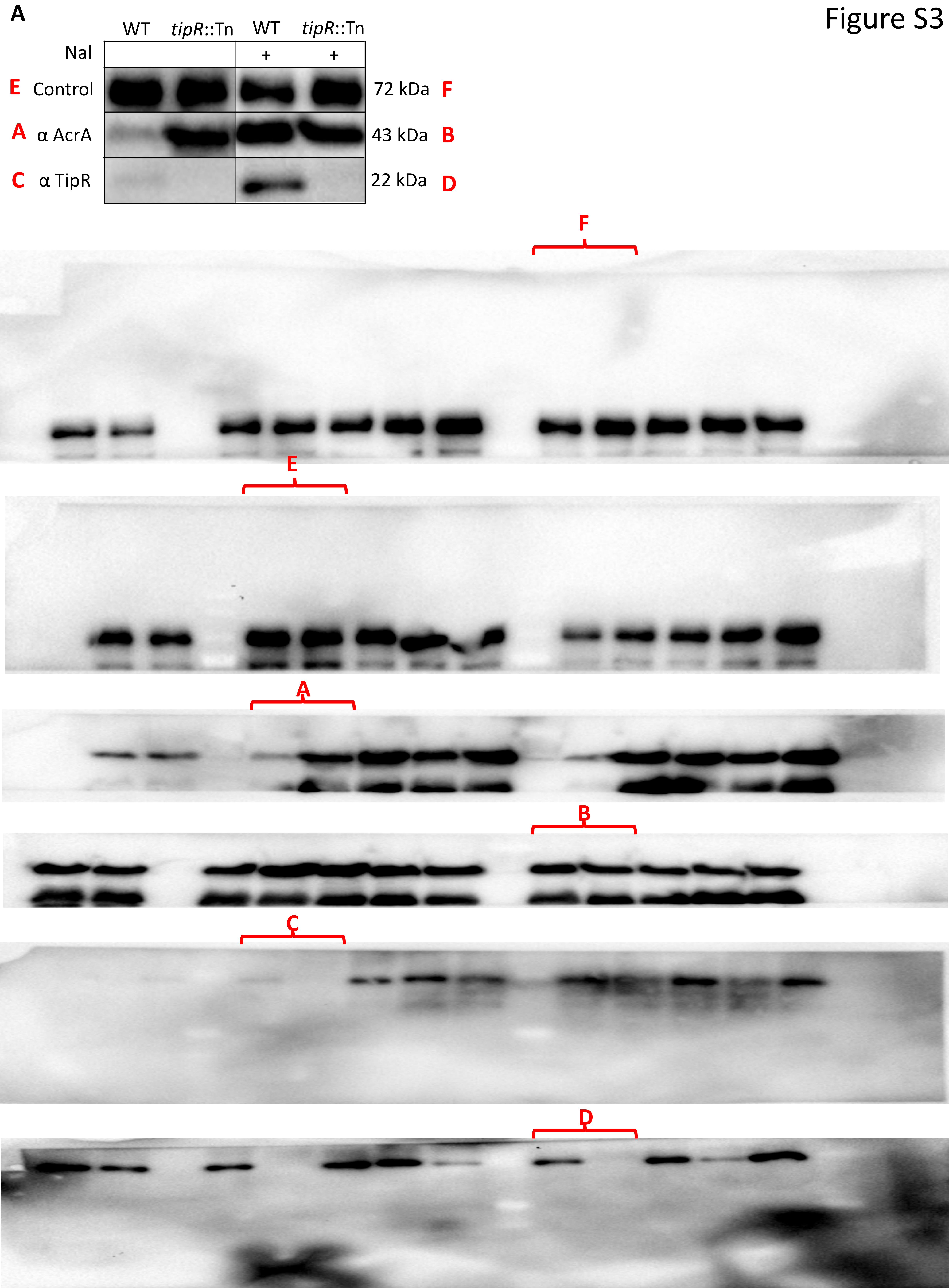
A
WT
tipR::Tn
WT
tipR::Tn
Nal
+
+
E
Control
72 kDa
F
A
α AcrA
43 kDa
B
C
α TipR
22 kDa
D
F
E
A
B
C
D

Figure S3

B

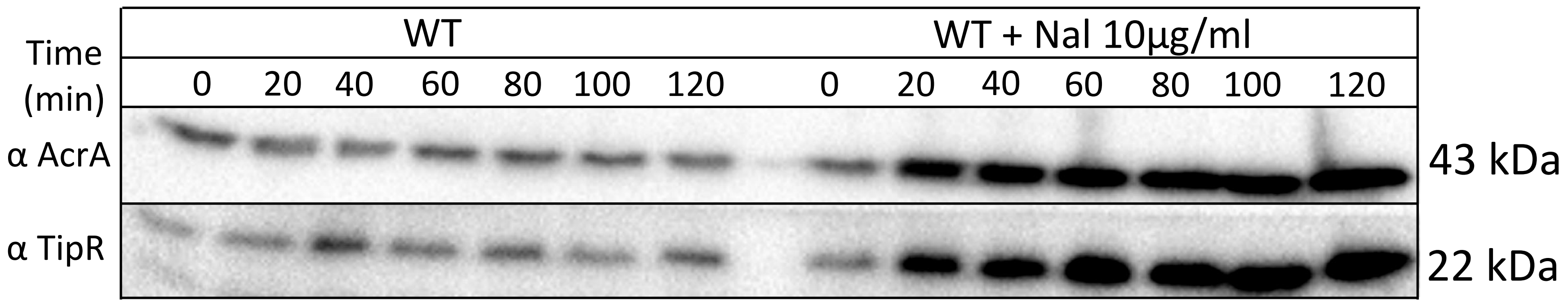

α AcrA

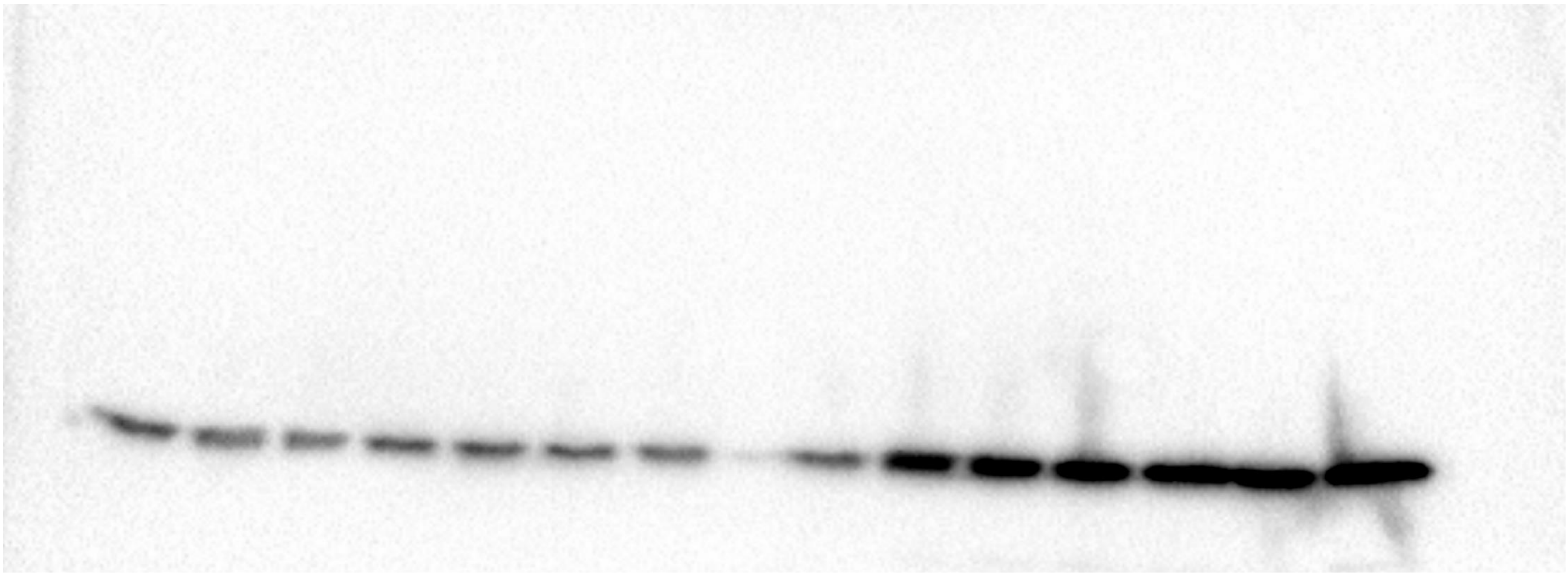

α TipR

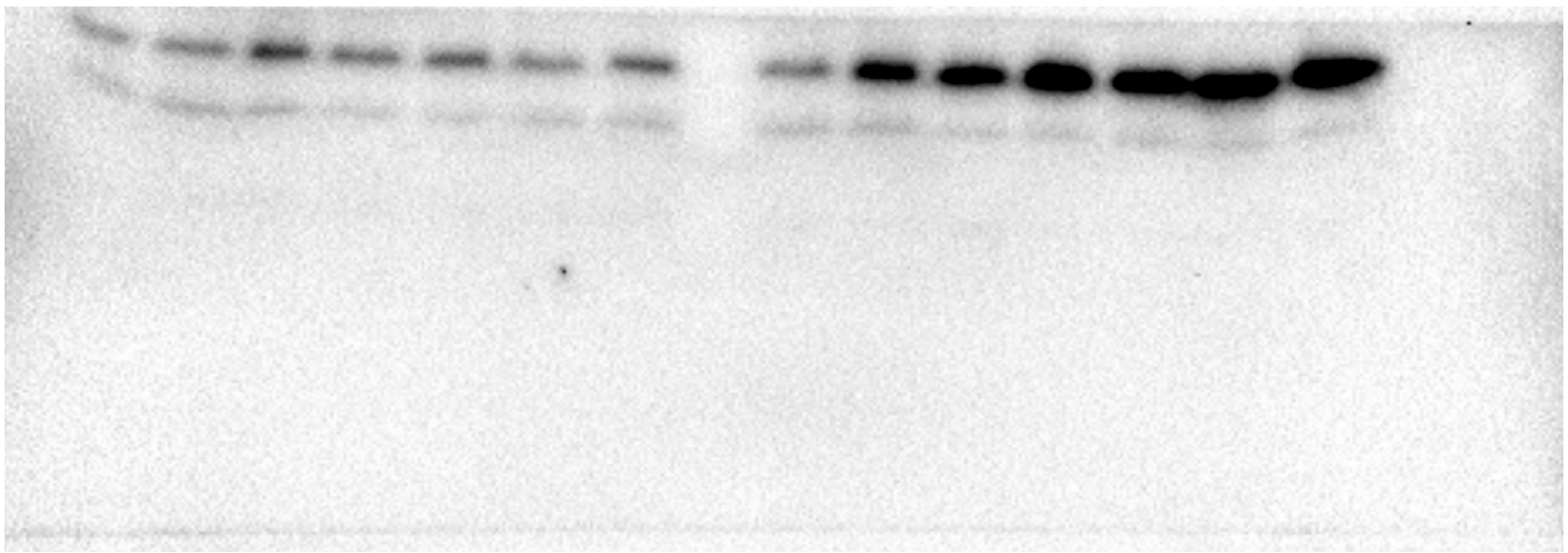

Figure S8

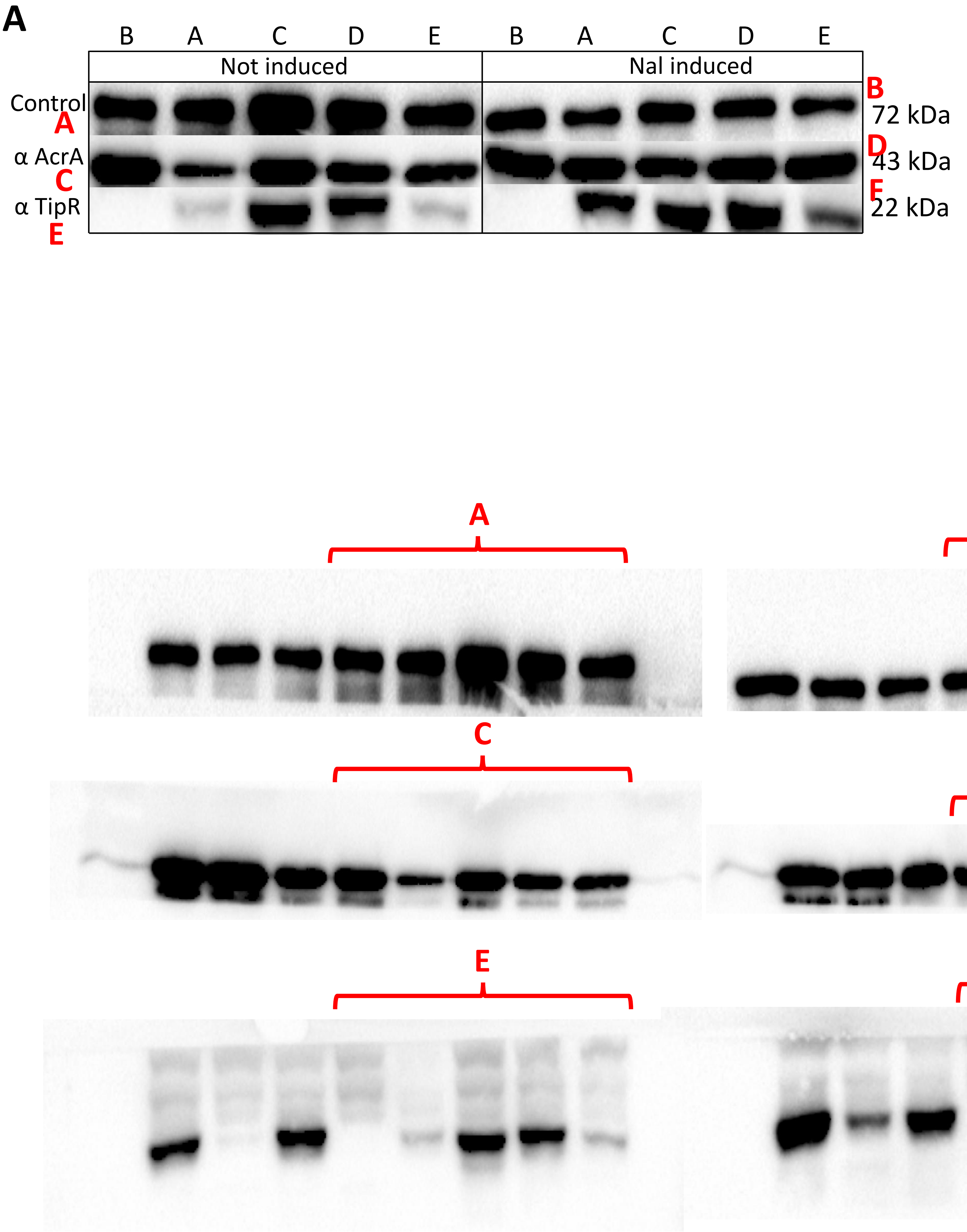

A Δ*bla*
B *tipR*::Tn
C Δ*bla*;*tipR*-E119V
D Δ*bla*;*tipR*-S53R
E Δ*bla*;*tipR*-L204Q
F Δ*bla*;P*acrA*-RIR
G Δ*bla*;P*acrA*-IR^{up}
H Δ*bla*;P*acrA*-MIR;pSRK*tipR*
I Δ*bla*
J Δ*bla*;P*acrA*-MIR;pSRK-Km
K Δ*bla*;pSRK-Km
L Δ*bla*;P*acrA*-2IR;pSRK-Km
M Δ*bla*;P*acrA*-IIR;pSRK-Km
N Δ*bla*;P*acrA*-IR^{dw};pSRK-Km

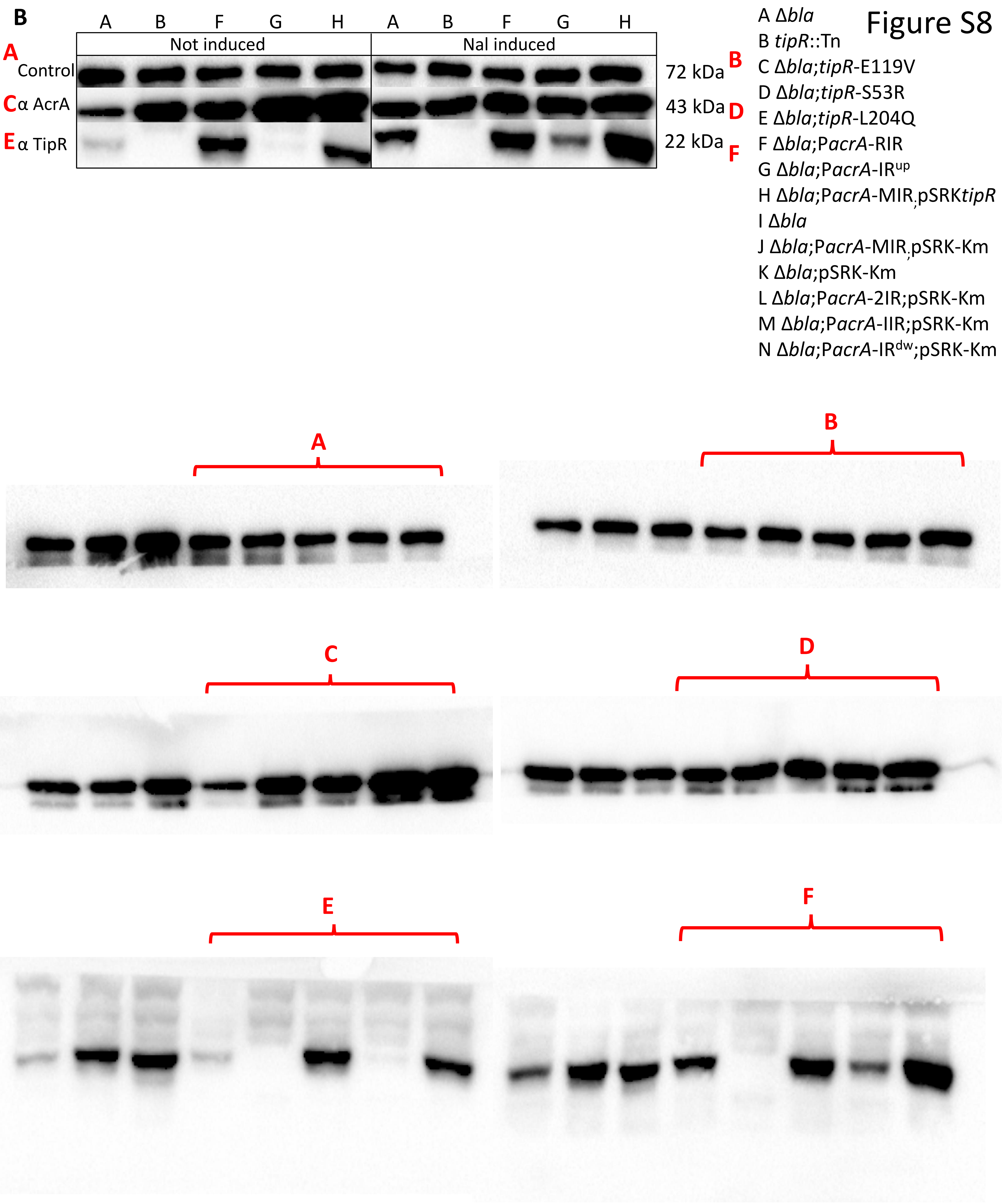
B
A
B
F
G
H
A
B
F
G
H
Not induced
Nal induced
A
Control
C α AcrA
E α TipR
72 kDa
43 kDa
22 kDa
B
D
F
A Δbla
B tipR::Tn
C Δbla;tipR-E119V
D Δbla;tipR-S53R
E Δbla;tipR-L204Q
F Δbla;PacrA-RIR
G Δbla;PacrA-IRup
H Δbla;PacrA-MIR;pSRKtipR
I Δbla
J Δbla;PacrA-MIR;pSRK-Km
K Δbla;pSRK-Km
L Δbla;PacrA-2IR;pSRK-Km
M Δbla;PacrA-IIR;pSRK-Km
N Δbla;PacrA-IRdw;pSRK-Km
A
B
C
D
E
F

Figure S8

Figure S8

c

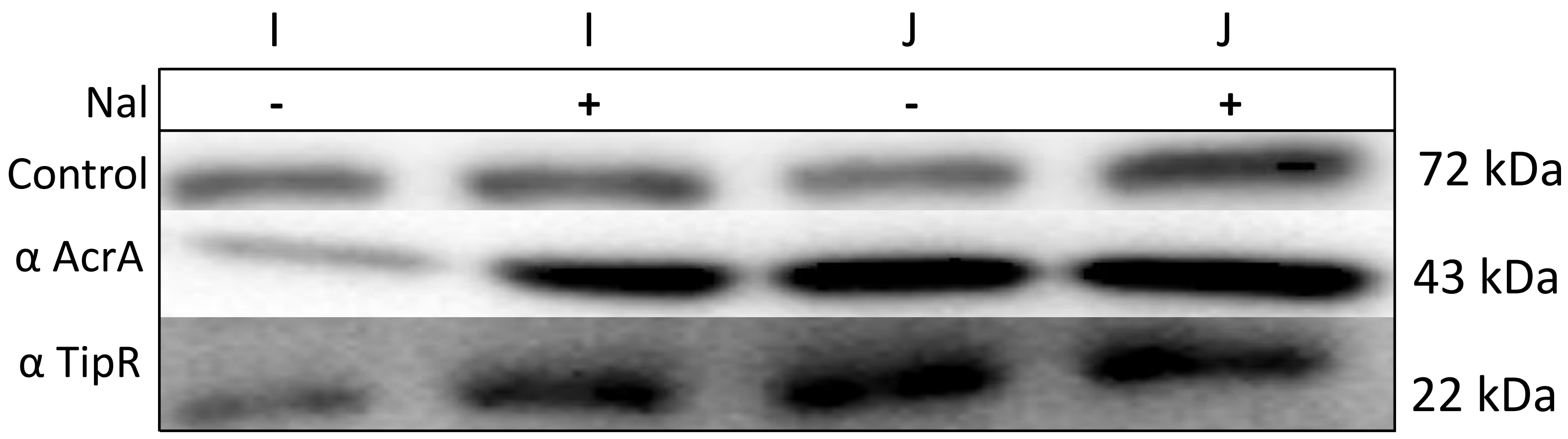

A Δ*bla*
B *tipR*::Tn
C Δ*bla*;*tipR*-E119V
D Δ*bla*;*tipR*-S53R
E Δ*bla*;*tipR*-L204Q
F Δ*bla*;*PacrA*-RIR
G Δ*bla*;*PacrA*-IR^{up}
H Δ*bla*;*PacrA*-MIR;pSRK*tipR*
I Δ*bla*
J Δ*bla*;*PacrA*-MIR;pSRK-Km
K Δ*bla*;pSRK-Km
L Δ*bla*;*PacrA*-2IR;pSRK-Km
M Δ*bla*;*PacrA*-IIR;pSRK-Km
N Δ*bla*;*PacrA*-IR^{dw};pSRK-Km

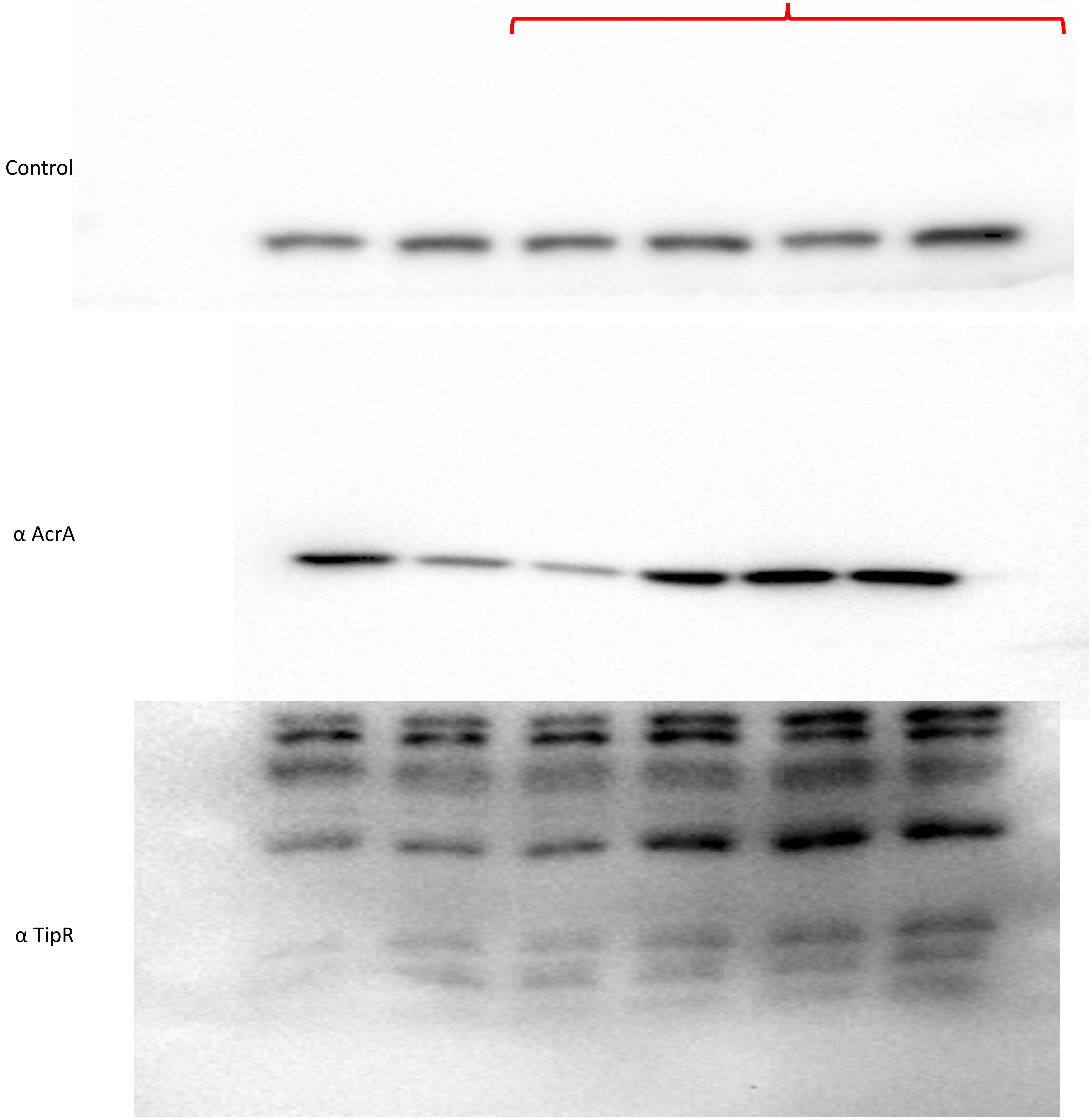

Figure S8

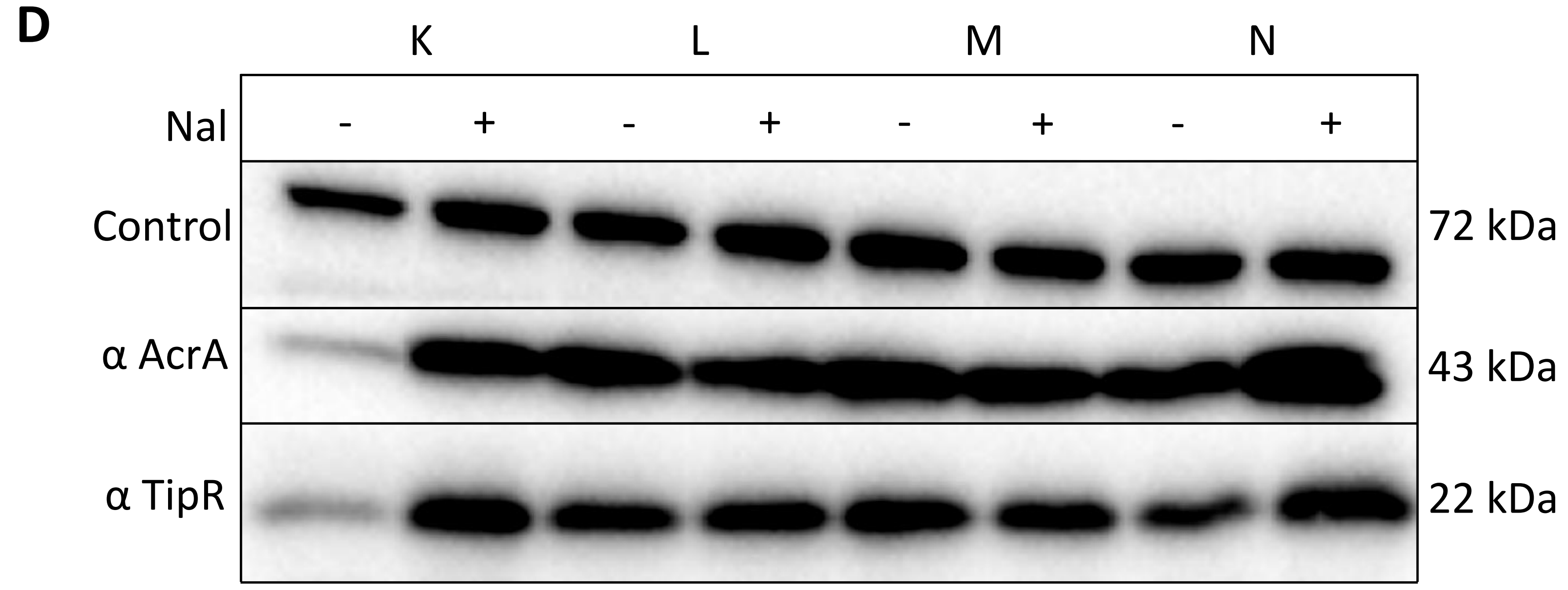

A Δ*bla*
B *tipR*::Tn
C Δ*bla*;*tipR*-E119V
D Δ*bla*;*tipR*-S53R
E Δ*bla*;*tipR*-L204Q
F Δ*bla*;P*acrA*-RIR
G Δ*bla*;P*acrA*-IR^{up}
H Δ*bla*;P*acrA*-MIR;pSRK*tipR*
I Δ*bla*
J Δ*bla*;P*acrA*-MIR;pSRK-Km
K Δ*bla*;pSRK-Km
L Δ*bla*;P*acrA*-2IR;pSRK-Km
M Δ*bla*;P*acrA*-IIR;pSRK-Km
N Δ*bla*;P*acrA*-IR^{dw};pSRK-Km

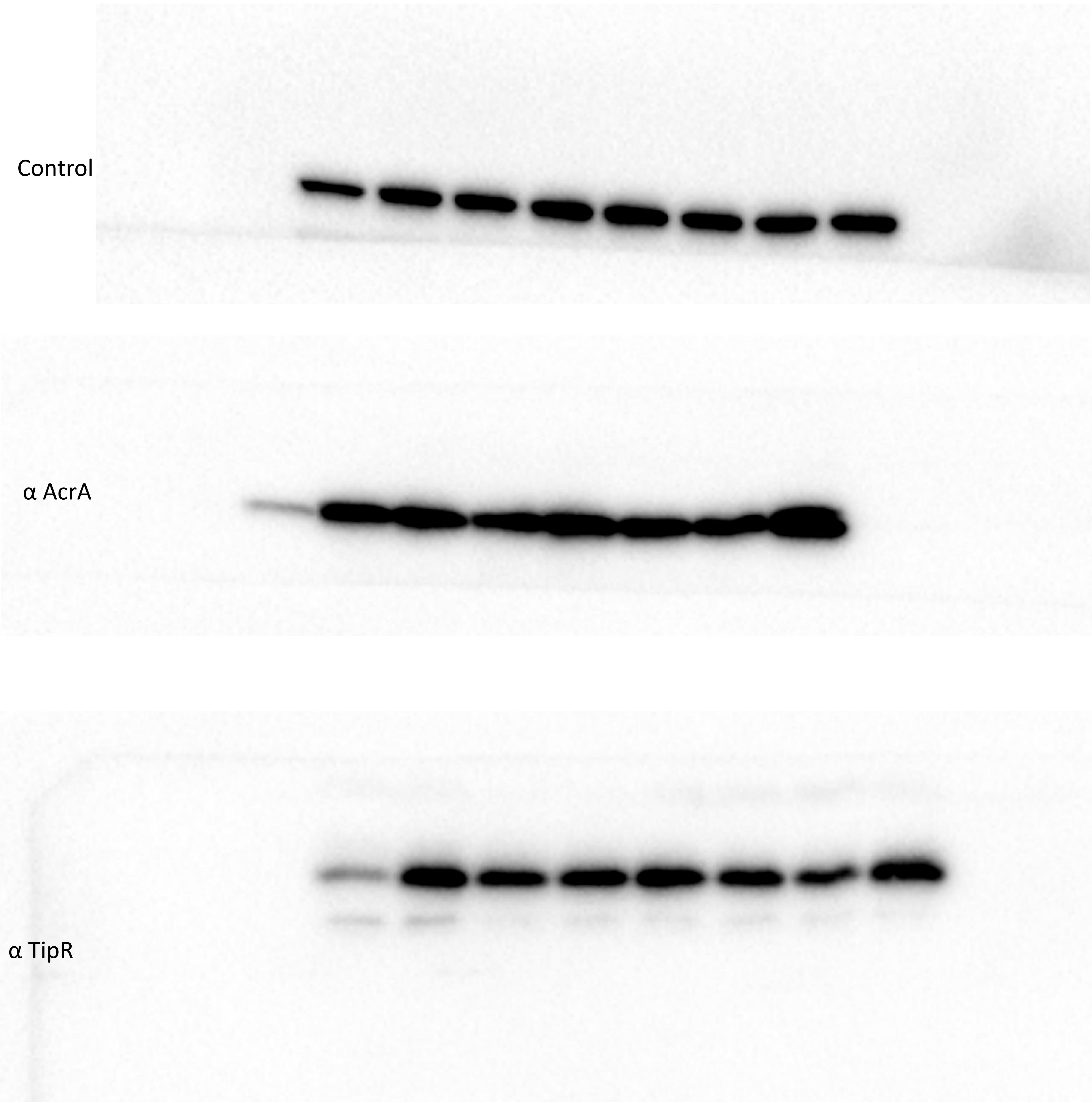

Figure S13

A

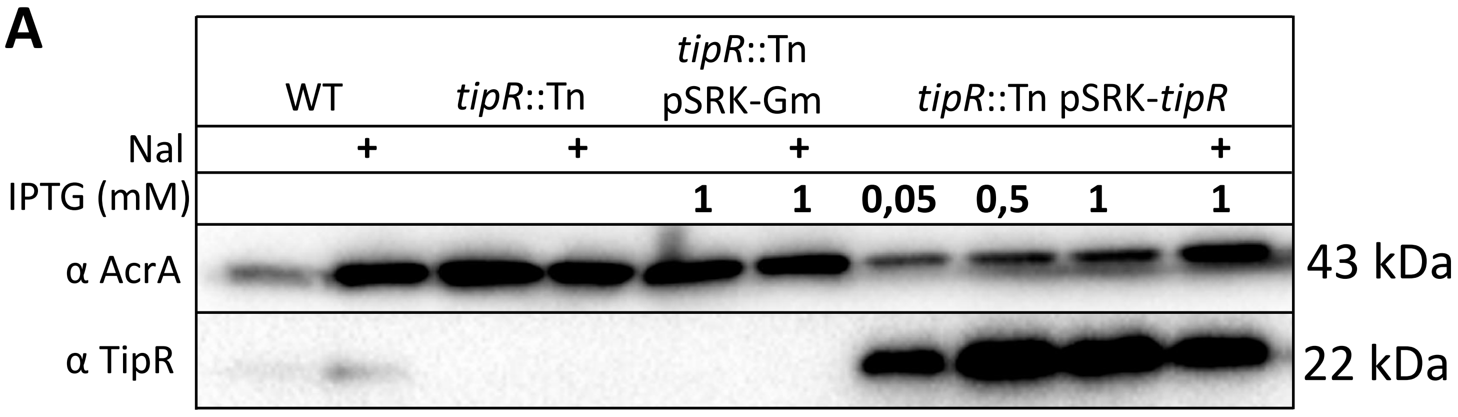

WT
tipR::Tn
tipR::Tn pSRK-Gm
tipR::Tn pSRK-tipR
Nal
+
+
+
+
IPTG (mM)
1
1
0,05
0,5
1
1
α AcrA
43 kDa
α TipR
22 kDa

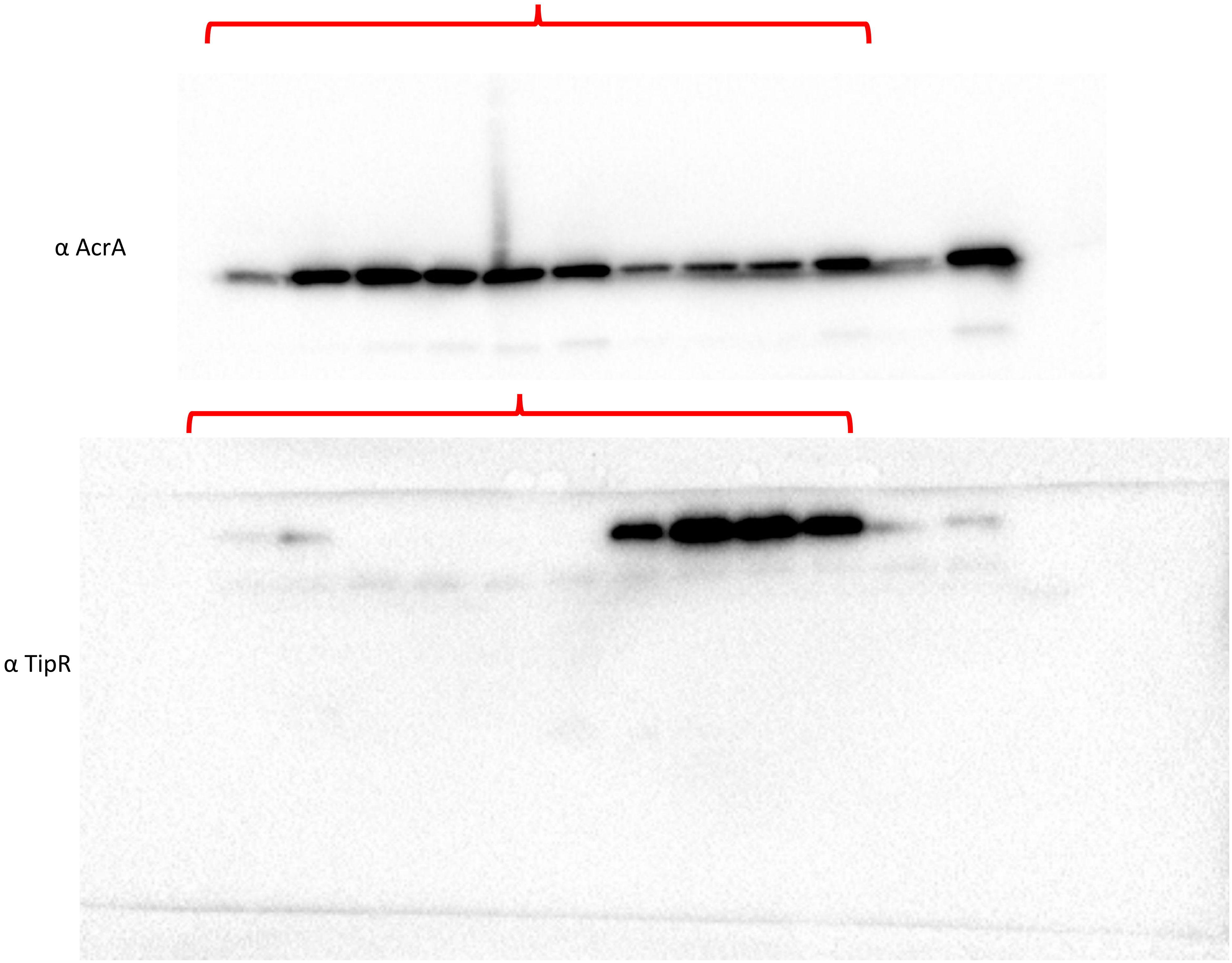

α AcrA
α TipR

Figure S13

B

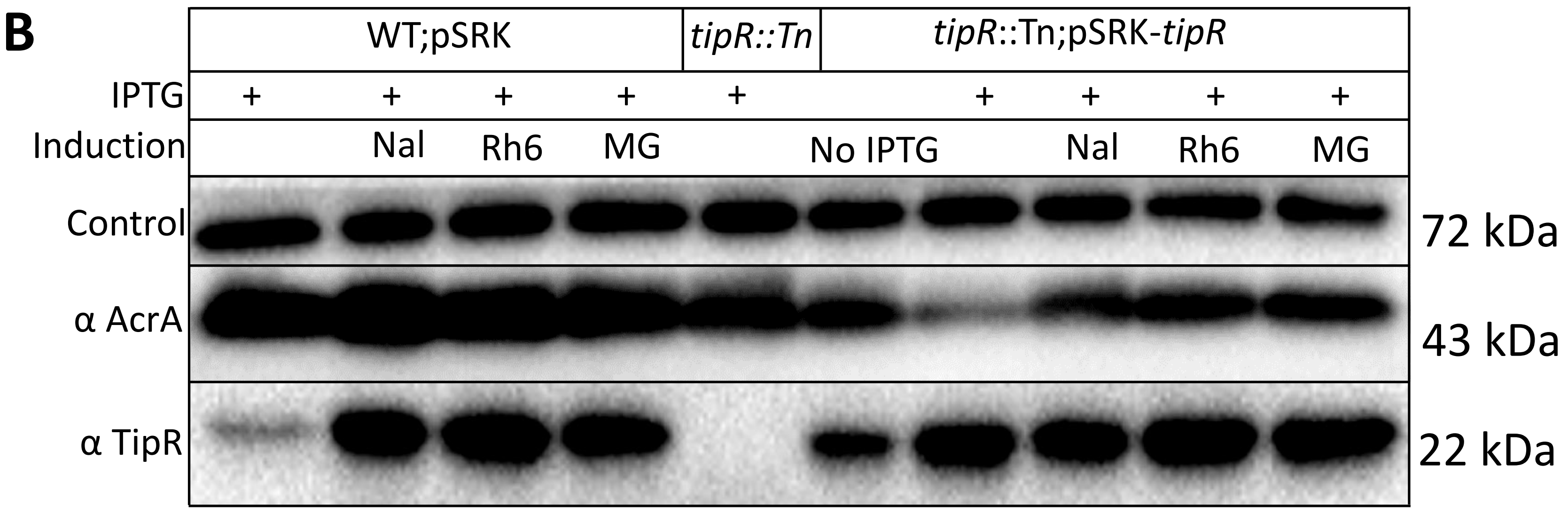

WT;pSRK
tipR::Tn
tipR::Tn;pSRK-tipR
IPTG
+
+
+
+
+
+
+
+
+
Induction
Nal
Rh6
MG
No IPTG
Nal
Rh6
MG
Control
α AcrA
α TipR
72 kDa
43 kDa
22 kDa

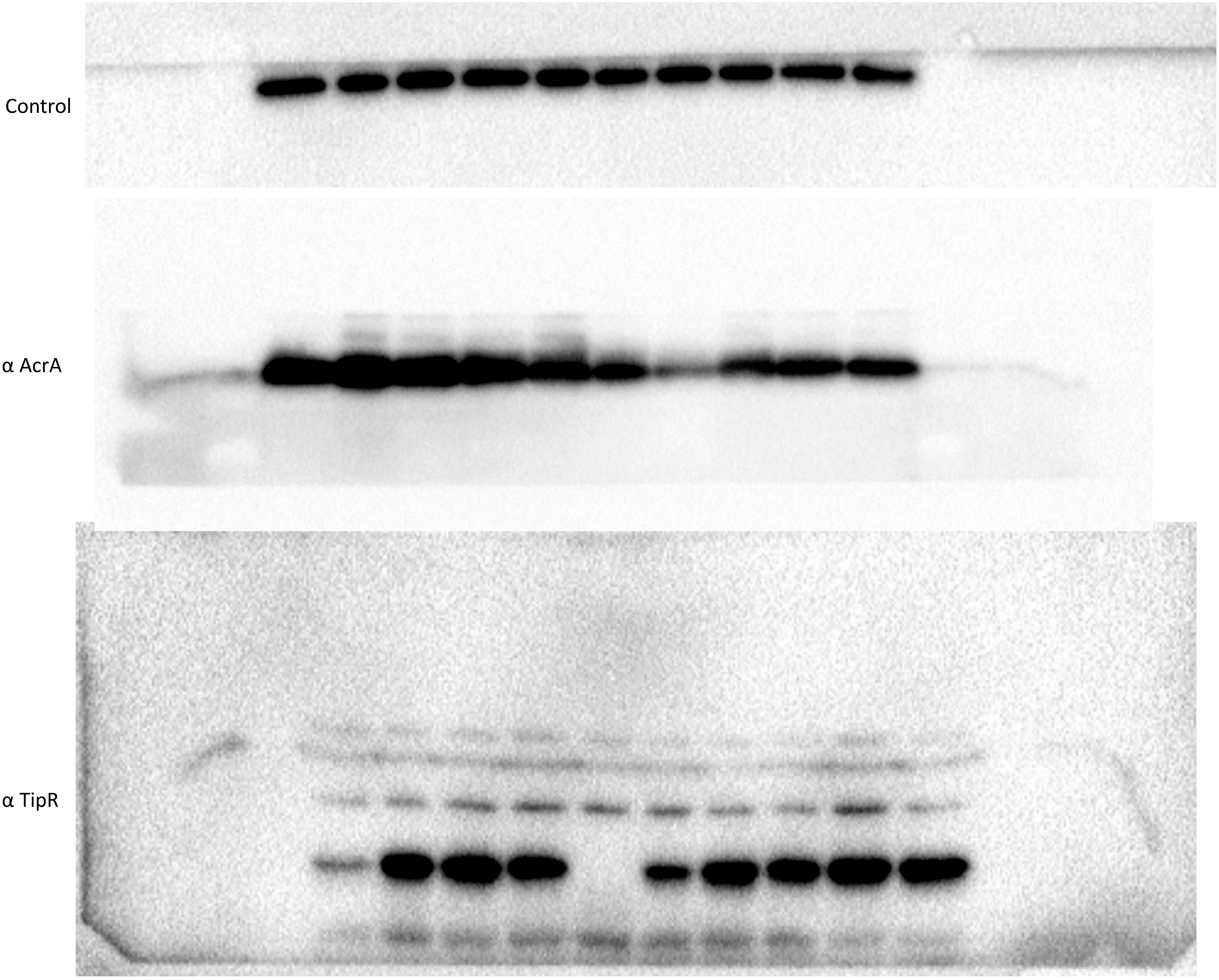

Control
α AcrA
α TipR

Figure S14

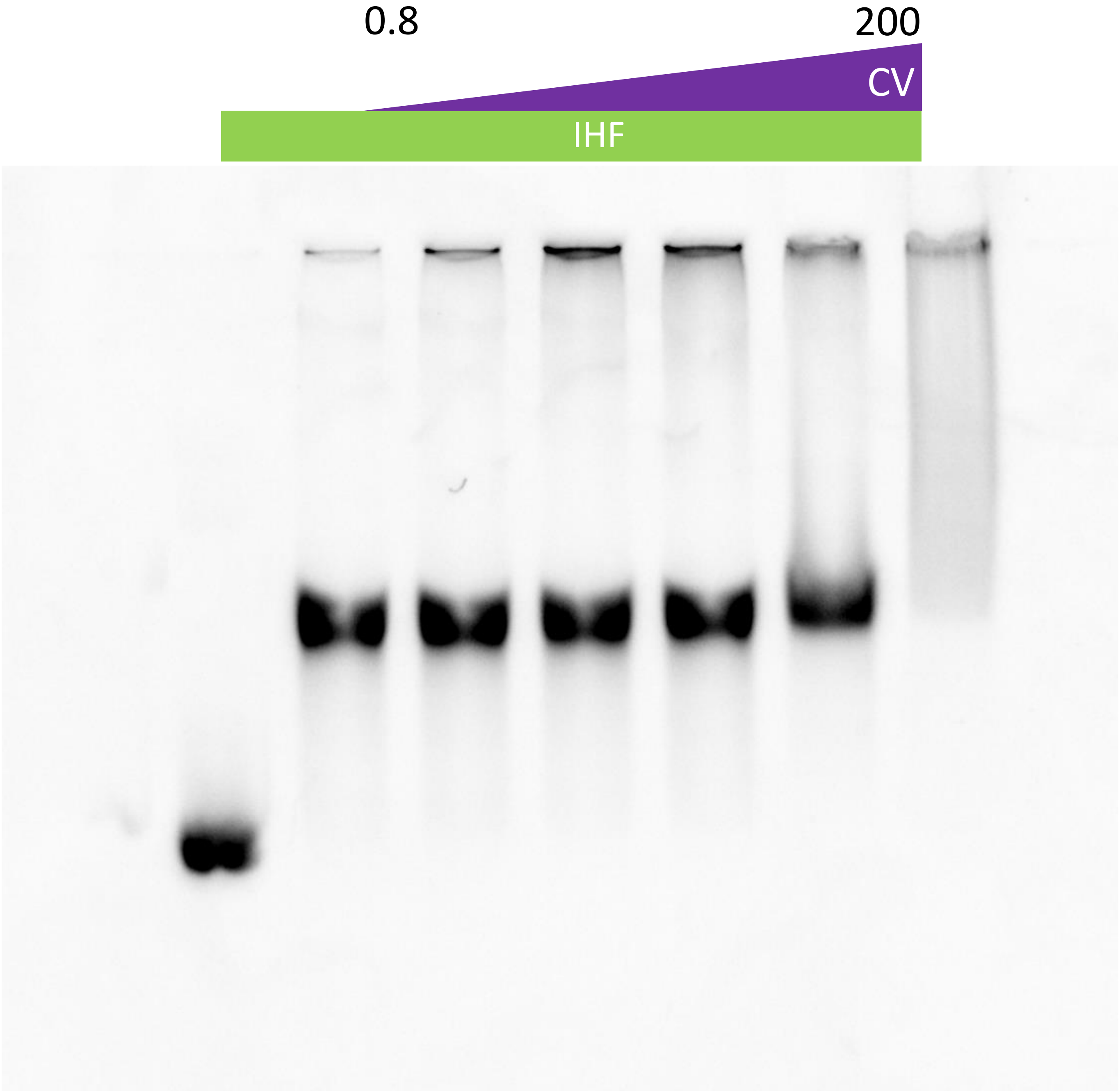

attR

Figure S16

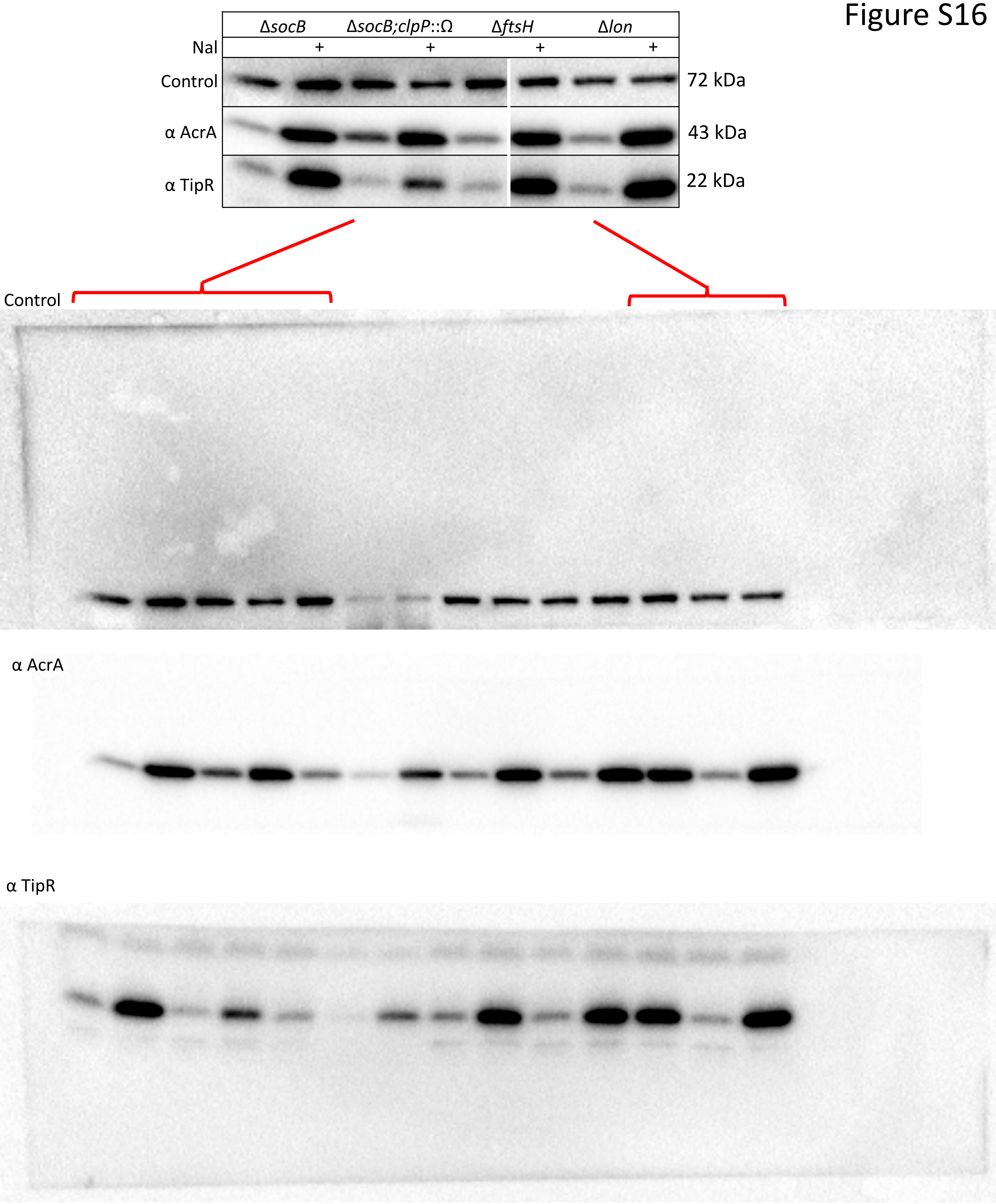
ΔsocB
ΔsocB;clpP::Ω
ΔftsH
Δlon
Nal
+
+
+
+
Control
α AcrA
α TipR
72 kDa
43 kDa
22 kDa
Control
α AcrA
α TipR

Figure S17

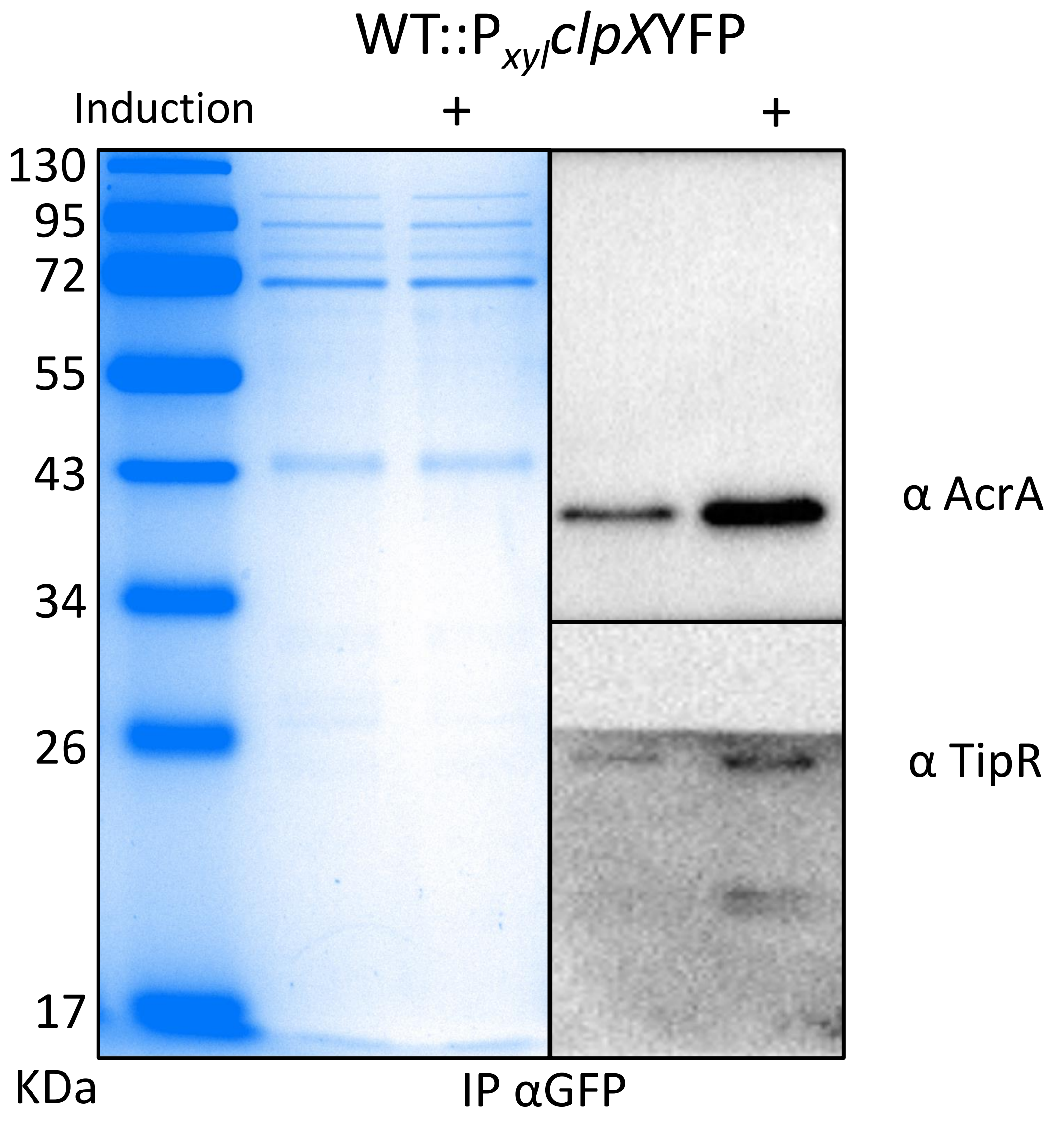
WT::P$_{xyl}$*clpX*YFP
Induction
+
+
130
95
72
55
43
34
26
17
KDa
IP αGFP
α AcrA
α TipR

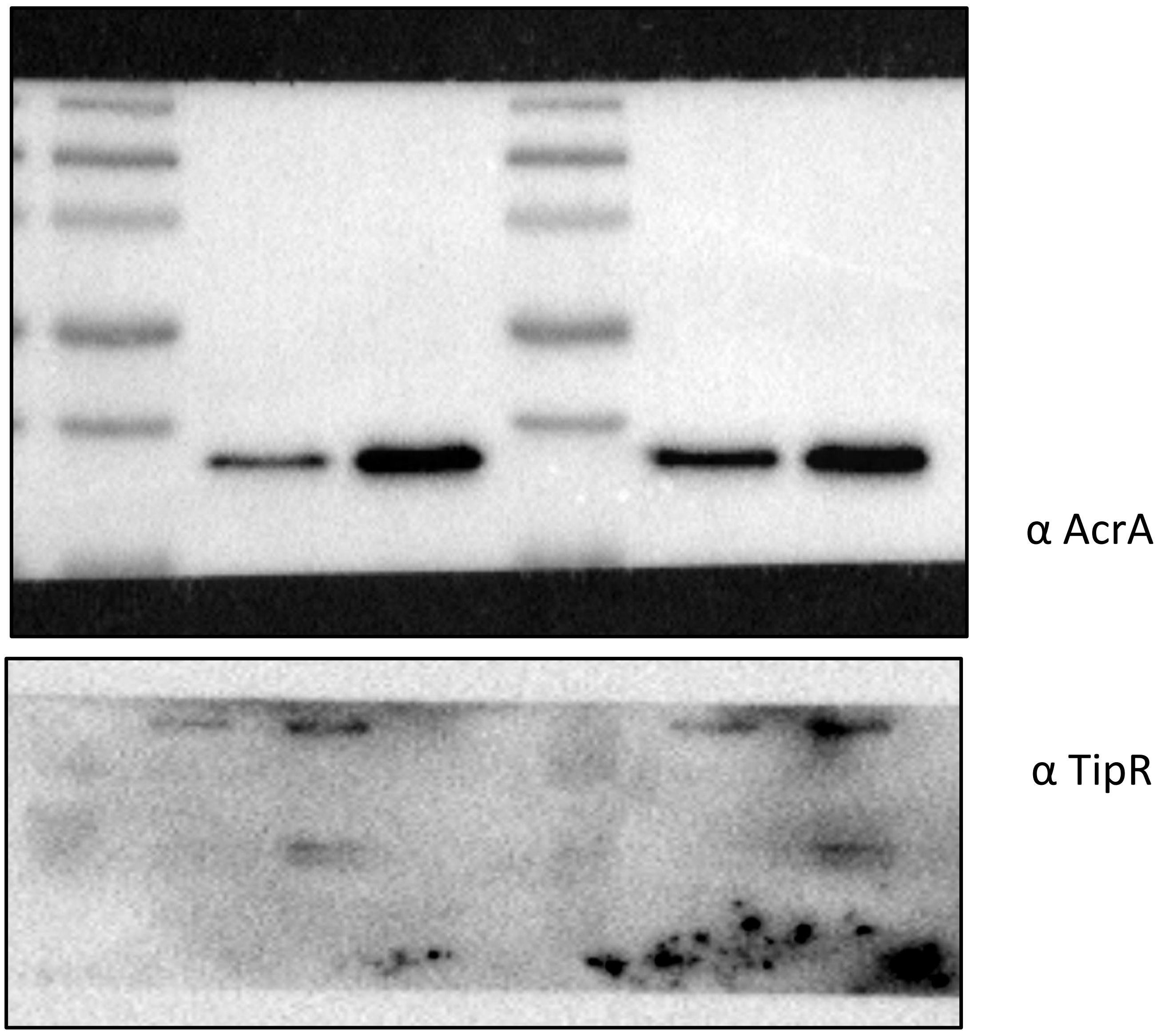
α AcrA
α TipR

Figure S18

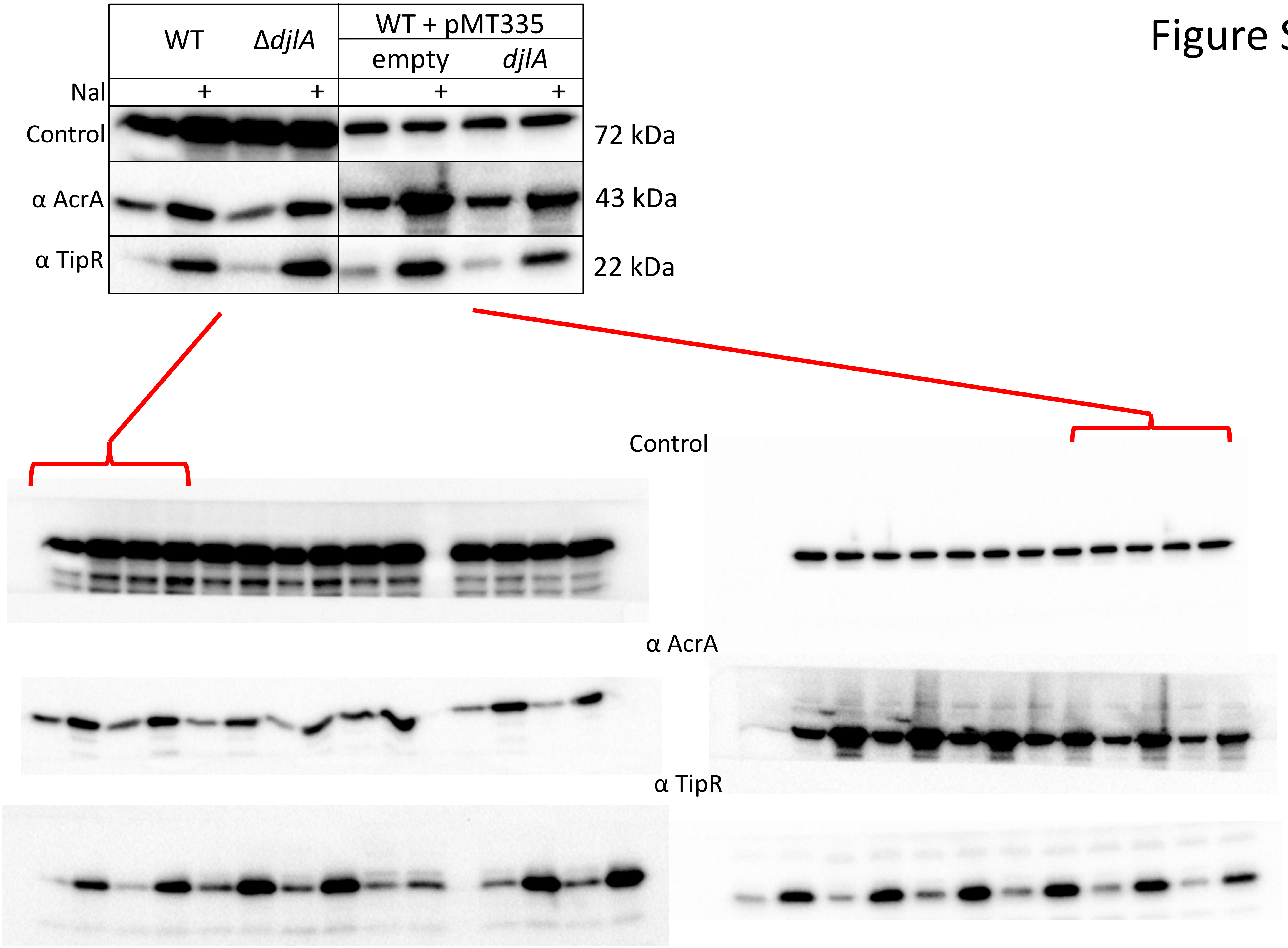
WT
ΔdjlA
WT + pMT335
empty
djlA
Nal
+
+
+
+
Control
α AcrA
α TipR
72 kDa
43 kDa
22 kDa
Control
α AcrA
α TipR

B

Figure S21

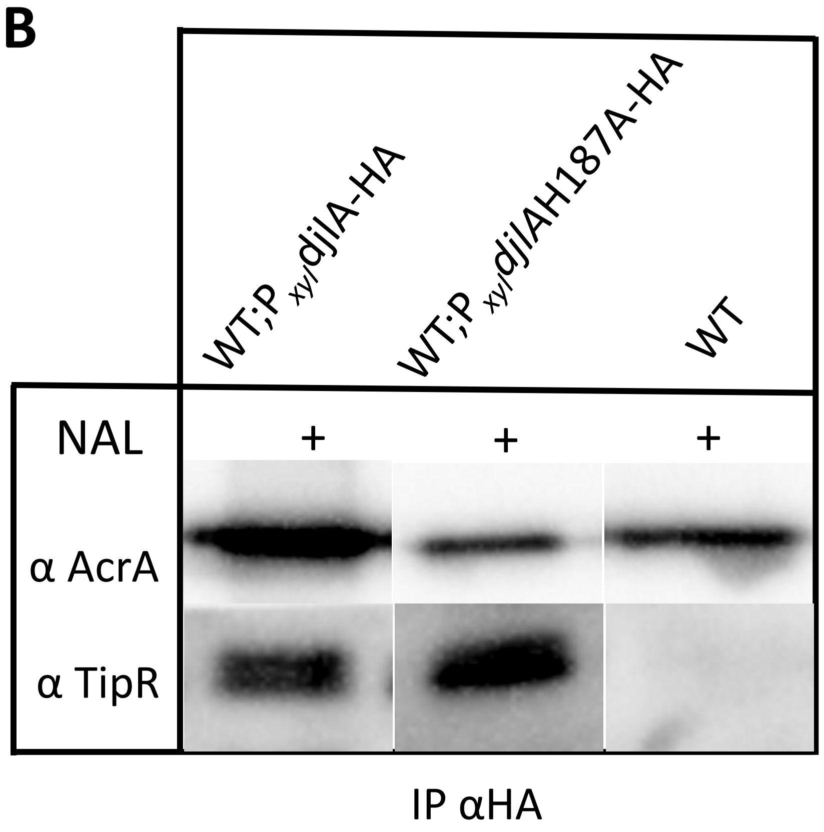
B
WT;P xyl djlA-HA
WT;P xyl djlAH187A-HA
WT
NAL
+
+
+
α AcrA
α TipR
IP αHA

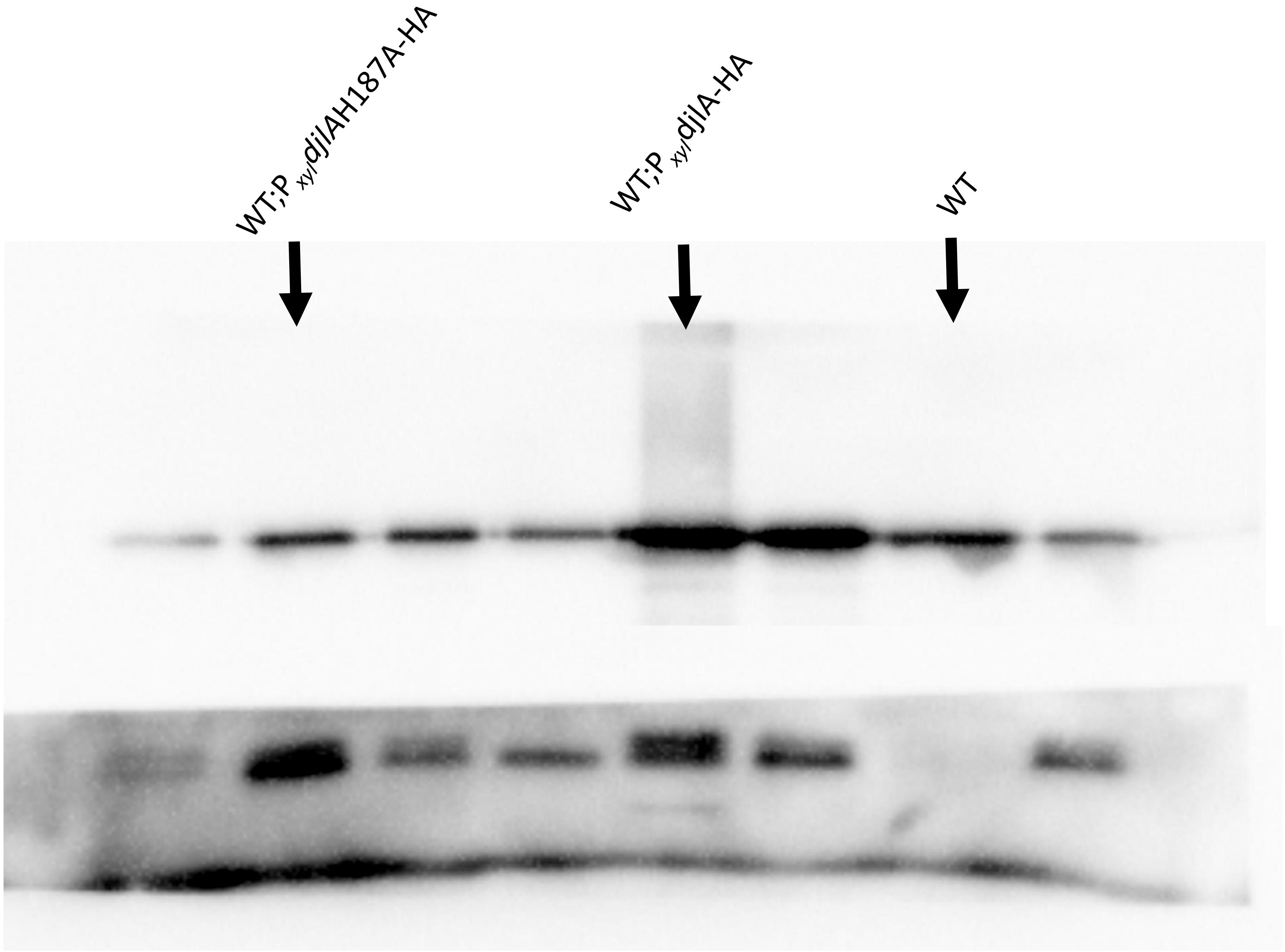
WT;P xyl djlAH187A-HA
WT;P xyl djlA-HA
WT

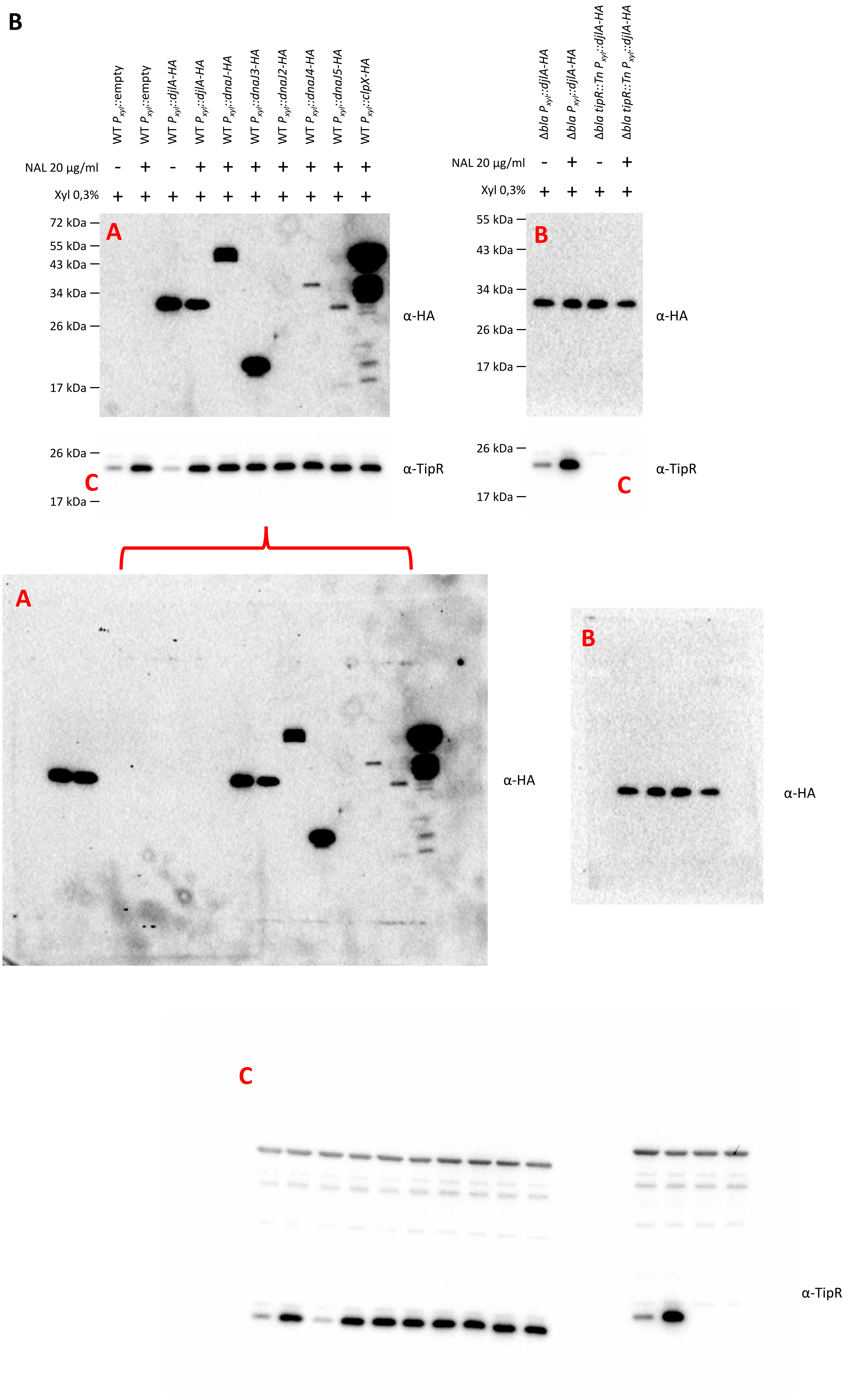
B
WT Pxyl::empty
WT Pxyl::empty
WT Pxyl::djlA-HA
WT Pxyl::djlA-HA
WT Pxyl::dnaJ-HA
WT Pxyl::dnaJ3-HA
WT Pxyl::dnaJ2-HA
WT Pxyl::dnaJ4-HA
WT Pxyl::dnaJ5-HA
WT Pxyl::clpX-HA
NAL 20 µg/ml - + - + + + + + + +
Xyl 0,3% + + + + + + + + + +
72 kDa
55 kDa
43 kDa
34 kDa
26 kDa
17 kDa
A
α-HA
26 kDa
17 kDa
C
α-TipR
Δbla Pxyl::djlA-HA
Δbla Pxyl::djlA-HA
Δbla tipR::Tn Pxyl::djlA-HA
Δbla tipR::Tn Pxyl::djlA-HA
NAL 20 µg/ml - + - +
Xyl 0,3% + + + +
55 kDa
43 kDa
34 kDa
26 kDa
17 kDa
B
α-HA
26 kDa
17 kDa
C
α-TipR
A
α-HA
B
α-HA
C
α-TipR

Figure S22
