# Supplementary material for: Adaptive β-lactam resistance from an inducible efflux pump that is post-translationally regulated by the DjlA co-chaperone
Source: PLoS Biol. 2023 Dec 5;21(12):e3002040. doi: 10.1371/journal.pbio.3002040 (PMC10754441; doi:10.1371/journal.pbio.3002040)
Supplement: Attachment — Submitted filename: response to reviewers.docx [file pbio.3002040.s032.docx]

**Reviewer #1:**

*This interesting work describes a curious phenomenon whereby nalidixic acid induces resistance to certain beta-lactams in Caulobacter. The activation involves the reversal of repression by a DNA binding transcription factor called TipR with the resulting expression of an efflux system. A particularly interesting detail is the further involvement of a chaperone protein. Overall I find the data to be convincing and the story sufficiently novel to merit serious consideration at PLoS Biology. There is significant room for improvement, however. As presented, the manuscript is not at all suitable for publication.*

The authors describe is a membrane blebbing phenomenon that accompanies activation. They concede at one point that they don't understand what this is all about and must speculate as to their content (lines 427-429). To me this is a distraction from the principal message of the paper and I would withdraw it.

We thank reviewer 1 for the constructive comments and the support.

The paragraph has been reduced and moved as to the supplementary data. We need to address the envelope perturbation caused by overexpression of AcrAB-NodT as it complements our results that the *tipR* mutant which overexpresses AcrAB-NodT not only exhibits elevated resistances but can also exhibit increased sensitivity to (some) large antibiotic molecules. We have also changed and compressed the section is describing these results and hope that this reorganization does not disturb the logical flow of the story.

Overall, we opted to keep the description of envelope defects, but to integrate these findings better into the flow of the manuscript. We consider this result important because we already published two cases where AcrAB-NodT induction by NAL is lethal in mutant cells (PMID: 33602809, 24726830) as this has not been mentioned before and because there is another study in the pipeline from our lab that further explores and builds on this phenotype with another envelope mutant which has not yet been reported. It is of course also possible that lengthy overexpression of other RND pumps, including in clinical isolates also causes envelope defects. This defect in *Caulobacter* also reconciles the vancomycin sensitivity arising from ectopic long-term AcrAB-NodT induction. Lastly, we feel that the phenotype and the supporting cryoET data fit very well with the observation that the massive AcrAB-NodT induction is genetically coordinated and augmented by the DjlA co-chaperone that we analysed in more detail in the revised submission (as detailed below).

We overhauled the text in the manuscript and streamlined the descriptions in several sections, in hopes of making it suitable for publication in PLOS BIOLOGY.

There is a tendency throughout to explain experimental results using adjectives rather than proper quantitative methods. There are many such instances however particularly egregious one is "pull-downs of DjlA-HA expressing cells grown in the absence of NAL, revealed an impressive number…". "Impressive" does not add anything - please give me a number.

Most of adjectives have been replaced by numeric values whenever possible.

I must add that the manuscript was a chore to read. There is a fair number of typos for example ("ptentially", "we also to obtain clones" etc…). A fair bit of the data is presented as a list of experiments lacking in context or hypothesis ("next, we…."). Finally, they employ an astoundingly excessive number of acronyms that leaves us with many passages that are virtually impossible to understand. NAL, NOV, IR, Rh6, WT, CV, EOP etc..etc… The result is unintelligible messes such as "In fact, the level of NAL-induction in cells expressing the NAL-sensitive forms of GyrA attains the level of induction when NAL and NOV are added jointly to WT cells, indicating that Gyrase inhibition can enhance AcrAB-NodT induction, but only after repression of PacrA by TipR has been relieved by the addition of NAL" and "Rh6 and CV affect TipR binding in the same manner, but less efficiently in vitro compared to EtBr or MG". A substantial amount of re-writing will be required before this
manuscript can go to press.
Thanks - the typos have been corrected (hopefully without introducing new ones), yet concerning the acronyms, we attempt to simplify some of the sentences, however there not many possibilities to reduce the number of acronyms, since our work describes many molecules and mutants.

**Reviewer #2:**

*Bacterial efflux pumps can confer adaptive resistance to a broad spectrum of antibiotics and are thus active players in the ongoing AMR burden. The manuscript by Costafrolaz and colleagues investigates in details the regulation and the impact of the AcrAB-NodT efflux pump of the Caulobacter crescentus model bacterium on multi-drugs resistance, highlighting a novel role for the DjlA/HSP40 cochaperone of HSP70 in vivo. The authors showed that induction of the AcrAB-NodT by nalidixic acid antibiotic confers protection against several β-lactams. In this case, they found that nalidixic acid induces the derepression of the Parc promoter of acrAB-nodT, which is under the control of the TipR protein, a TetR-like repressor. Control by TipR was then investigated in details, revealing key residues of TipR and important responsive regulatory elements within the promoters and the intergenic region of AcrAB-NodT and TipR. Such analysis revealed that TipR also controls
expression of the DjlA cochaperone. In addition, they found that the stress protease ClpXP participates both in the instability of TipR and the transcriptional induction of acrAB-nodT in the presence of nalidixic acid. Further genetic screens led to the identification of several chemicals that activate acrAB-nodT either by interfering with tipR DNA-binding or by affecting its stability. Remarkably, the authors also found that overexpression of the AcrAB-NodT efflux pump led to membrane defects, revealing intriguing interplay between an increased resistance related to pump overexpression and its consequence on the integrity of the outer membrane and the observed increased sensitivity to other type of drugs that would normally be harmless. Finally, the authors found that the TipR-regulated DjlA cochaperone interacts with the AcrAB-NodT pump and is capable of enhancing the resistance capacity to several B-lactams, thus revealing its impact on drug efflux.
The manuscript is well written and presents very interesting and novel data of general interest. Yet, additional controls need to be performed in order to strengthen the proposed role of the cochaperone DjlA*.

**Major comments:**
**1-** Data form Figure 6E suggest that DjlA overexpression increases the soluble fraction of AcrA and that ArcA is more soluble in the WT than in the djlA mutants. However, the current data from the western blots are not convincing, with differences hardly visible. The authors should quantify the bands obtained for several independent experiments. In addition, it seems that DjlA overexpression does not significantly increase the soluble fraction but prevents the accumulation of the insoluble form of ArcA. The robustness of these data is critical in order to demonstrate that DjlA is indeed contributing to the efficient assembly/activity in vivo.

We appreciate the reviewer’s suggestions and we have now quantified the relative proportion of soluble versus insoluble amount of AcrA in 4 replicates. We measured the aggregation of AcrA by determining the fraction of insoluble versus soluble AcrA and found that the former increases by 40% under Nal-inducing conditions in the absence of DjlA or, conversely, decreases when AcrA is overexpressed from plasmid pMT335. The increased quantity of AcrA in the soluble fraction as inferred previously was based on the incorrect assumption that the total amount of protein seemed similar and if the protein is not insoluble, it should be soluble, rather than directly determining the total amount, as well as the amount in soluble and insoluble fractions. The current analysis corrects for this misconception and is now presented in supplementary figure S23.

**2-**The role of the cochaperone is never presented within the context of its HSP70/DnaK partner. Providing evidences that we are indeed looking at a DnaK cochaperone activity and not at a DnaK-independent function is thus essential. Since DnaK is essential, the authors should test the impact of the canonical His to Gln loop substitution in the J-domain of DjlA in their drop test assay and/or in the fractionation experiments. Such a substitution in J-domain cochaperone is known to abolish the interaction with DnaK without perturbing membrane assembly or substrate interaction. This is a simple but powerful test to bring in the whole DnaK machine in enhancing pump activity, and thus confirm that the DnaK cochaperone activity is indeed responsible for the phenotypes observed.

We thank the reviewer for raising this important point. The additional experiments described in above were included in the revised version, bolstering our conclusions that DjlA interacts with TipR and AcrA in multiple ways.

First, we surveyed a panel of DnaJ orthologs (DnaJ, DnaJ2, DnaJ3, DnaJ4, DnaJ5, as well as ClpX) that harbour a C-terminal HA-tag and determined that only DjlA-HA occupies the *acrAB* promoter as determined by ChIP-Seq. Moreover, ChIP-Seq analysis conducted in *tipR*:.Tn cells expressing DjlA-HA no longer showed a statistically significant peak at the *acrAB* promoter, confirming that DjlA-HA depends on TipR for its recruitment to the *acrAB* promoter. These findings are shown in Figure S23.

We also tested these HA-tagged DnaJ derivatives for interaction with TipR by ChIP-Seq and found that only DjlA-HA was able to interact with TipR at PacrA (figure S22).

Finally, as requested by the reviewer, we engineered mutant versions of DjlA-HA in which the key histidine in the HPD domain was altered (DjlA-H187A and DjlA-H187Q) that would normally negatively impact proper ATPase activity of DnaK and, therefore, chaperone activity on the DnaJ/DjlA cargo. The results obtained after pulldown show that TipR is no longer associated with the two DjlA variants while AcrA still is. Neither variant was able to confer DjlA activity in vivo as determined by EOP assays.

In summary, these additional experiments demonstrate that DjlA is indeed recruited for TipR and AcrA functions and therefore the genetic co-ordination of this tripartite system at the level of gene expression makes sense.

**3-**The proposed model (Figure 6G) for TipR and AcrAB-NodT interaction with DjlA does not take into account the fact that DjlA is a membrane anchor protein. Would a membrane localization of DjlA fit in this model?  This should be discussed and the model adapted accordingly.
*Caulobacter* crescentus DjlA does not have a transmembrane anchor, unlike E. coli DjlA. To avoid confusion, we now mention this in the text and discussion.

**Minor comments:**
**1-**It is known that there is some overlap between J-domain cochaperones, especially in the presence of the stress-responsive DnaJ. The fact that endogenous DnaJ is present could explain the week impact of a single djlA mutation on pump activity. This should be discussed.

We thank the reviewer for pointing this out. Indeed, our survey of pulldowns conducted with other HA-tagged DnaJ paralogs revealed that DnaJ (CCNA_00011, aka DnaJ1), but not DnaJ2-5 bind AcrA. We have now included a reference to the overlapping activity in the discussion and a brief comment stating this possibility,

**2-**The previously described function of DjlA in the enhancement of recombinant membrane protein production and in the proper function of the Dot/Icm secretion complex should be discussed in light of the data presented in this work.

Done

**3-**The ChiP-Seq data from Figure 6D suggest that DjlA-HA binds PacrA via TipR. Do the authors have evidence suggesting that DjlA-HA does not bind PacrA in the absence of TipR? This should be discussed.

The ChIP-Seq experiment were done in absence of TipR (figure S22) and do not show anymore occupancy of the acrAB promoter by DjlA. Additionally, all DnaJ-like protein of Caulobacter were used as control in similar ChIP-Seq experiments and none of them other than DjlA occupy the *tipR/acrA* locus.
